# A regioselectivity switch in Pd-catalyzed hydroallylation of alkynes†
†Electronic supplementary information (ESI) available. See DOI: 10.1039/c9sc01527b


**DOI:** 10.1039/c9sc01527b

**Published:** 2019-05-16

**Authors:** Ding-Wei Ji, Yan-Cheng Hu, Hao Zheng, Chao-Yang Zhao, Qing-An Chen, Vy M. Dong

**Affiliations:** a Dalian Institute of Chemical Physics , Chinese Academy of Sciences , Dalian 116023 , P. R. China . Email: qachen@dicp.ac.cn; b University of Chinese Academy of Sciences , Beijing 100049 , P. R. China; c Department of Chemistry , University of California , Irvine , California 92697-2025 , USA

## Abstract

By exploiting the reactivity of a vinyl-Pd species, we control the regioselectivity in hydroallylation of alkynes under Pd-hydride catalysis. A monophosphine ligand and carboxylic acid combination promotes 1,5-dienes through a pathway involving isomerization of alkynes to allenes. In contrast, a bisphosphine ligand and copper cocatalyst favor 1,4-dienes *via* a mechanism that involves transmetalation. Our study highlights how to access different isomers by diverting a common organometallic intermediate.

## Abstract 2

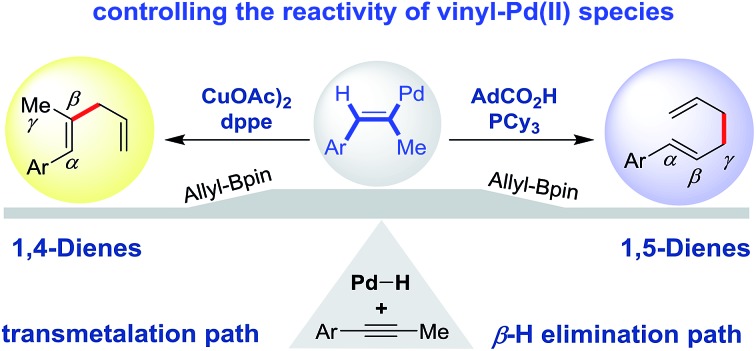
Through rational evaluation of ligands and promoters, the reactivity of a key Pd(ii) species towards transmetalation or β-H elimination is manipulated.

## Introduction

**Scheme 1:**
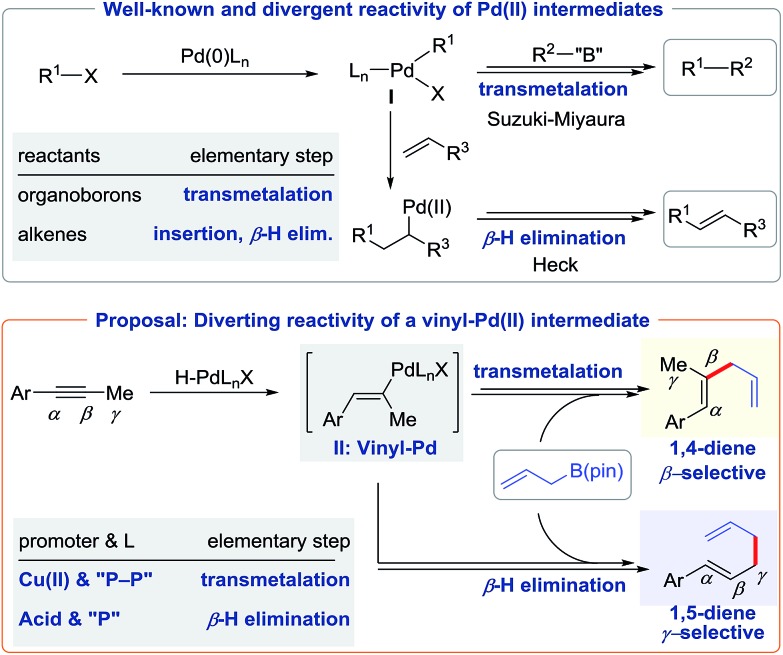
Divergent reactivity of Pd(ii) species.

Inventing novel cross-couplings has relied upon our ability to divert readily accessible and common organometallic intermediates.^1^ For example, Pd(ii) intermediates **I** (generated from organic halides or their analogues) can transmetallate with organoborons in a Suzuki–Miyaura cross-coupling^2^ or undergo insertion into olefins and subsequent β-hydride elimination in the Heck reaction (Scheme 1, top).^3^ Inspired by the power of this concept, we set out to divert the reactivity of a vinyl-Pd(ii) intermediate **II** (generated from alkynes, Scheme 1, bottom) to achieve useful skipped dienes.

The hydroallylation of alkynes has attracted attention due to the occurrence of skipped dienes in bioactive compounds and natural products.^4^ While various catalysts have been developed to generate the 1,4-diene motif,^5^ access to the 1,5-diene isomer *via* hydroallylation of alkynes has been elusive. In considering this challenge, we were inspired by the work of Trost,^6^ Yamamoto,^7^ and Breit^8^ who have used alkynes as redox-neutral allyl precursors.^9^–^12^ Early studies established that Pd–H can add to a alkyne to generate the vinyl-Pd species **II**. With this in mind, we set out to manipulate the reactivity of this Pd(ii) species **II** towards transmetalation or β-H elimination to enable selective access to 1,4 or 1,5-dienes, respectively (Scheme 1, bottom). Herein, we report the use of ligands and promoters to enable a regiodivergent synthesis of skipped dienes. Our study contributes to the art of diverging catalytic intermediates to access different constitutional isomers.^13^

## Results and discussion

**Chart 1:**
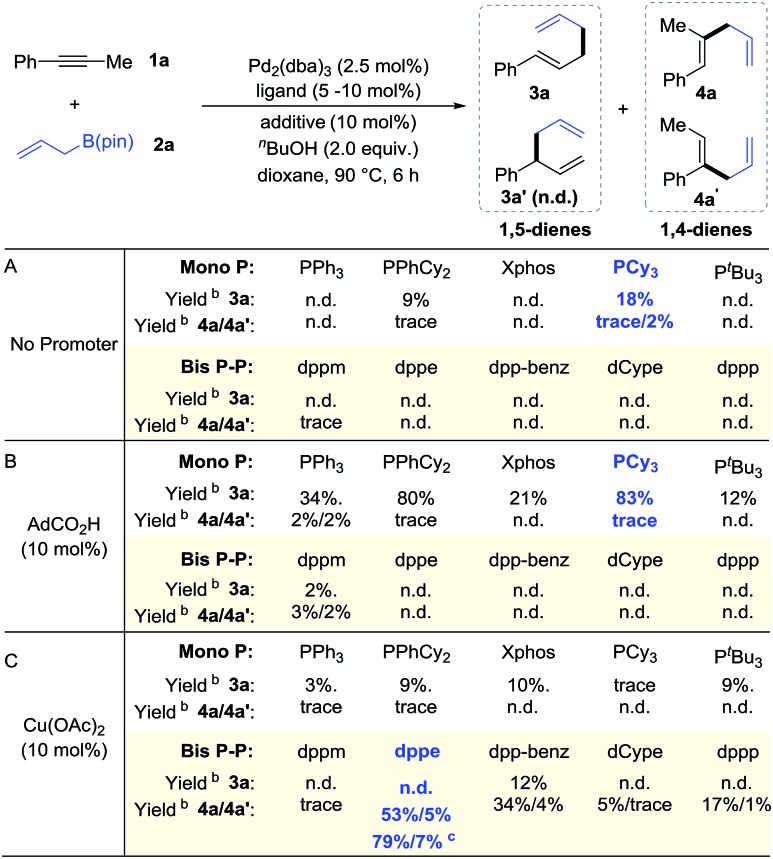
Ligand and promoter effects on hydroallylation. ^a^**1a** (0.20 mmol), **2a** (2.0 equiv.), Pd_2_(dba)_3_ (2.5 mol%), monophosphine (10 mol%) or bis-phosphine (5 mol%), promoter (10 mol%), ^*n*^BuOH (2.0 equiv.), dioxane (1.0 mL), 90 °C, and 6 h. ^b^Determined by ^1^H NMR or GC-FID with 1,3,5-trimethoxybenzene as the internal standard. ^c^MeOH (3.0 equiv.) instead of ^*n*^BuOH, dioxane (0.5 mL), 70 °C, and 24 h.

We began our study with 1-phenyl-1-propyne **1a** and allyl-B(pin) **2a** as the model substrates. After examining various combinations of ligands and additives, we obtained compelling results (Chart 1). In the presence of Pd_2_(dba)_3_ (2.5 mol%) and a proton source ^*n*^BuOH (2.0 equiv.), we found that monophosphine ligands such as PPhCy_2_ and PCy_3_ could give 1,5-diene (Chart 1A) as the major product. With the aid of PCy_3_, 1,5-diene **3a** could be obtained in 18% yield accompanied by trace amounts of 1,4-dienes **4a** and **4a′** mixture. To improve the yield of 1,5-diene **3a**, we chose Brønsted acid to facilitate the formation of an active Pd(ii)–H catalyst.^7^ By adding 10 mol% 1-adamantanecarboxylic acid, we obtained **3a** in 83% yield with excellent selectivity (Chart 1B). Bisphosphine ligands gave only trace amounts of 1,5-diene **3a** because these ligands occupy the otherwise vacant sites needed for β-hydride elimination.^1^,^14^

Next, we aimed to selectively prepare the 1,4-diene **4a**, which is the minor isomer that results from direct coupling of alkyne **1a** and allylboron **2a**. To alter regioselectivity, we pursued a co-catalyst that would accelerate transmetalation between the allyl species and the vinyl-Pd intermediate, and therefore enable the synthesis of 1,4-dienes. An evaluation of co-catalysts revealed that Cu(OAc)_2_ promoted the formation of **4a**/**4a′** (Chart 1C). Moreover, in contrast to the monophosphine ligands that promotes the formation of **3a**, bisphosphine ligands such as dppe and dpp-benz gave a higher yield of **4a**/**4a′**. When dppe was used as the ligand, **4a**/**4a′** was delivered in 58% combined yield as a 10 : 1 mixture of regioisomers. Using methanol instead of butanol as the proton source improved the yield of **4a** to 79%. In the absence of palladium, no products were detected, which indicates that this transformation is not catalyzed by copper alone.

As shown in Table 1A, we obtained various 1,5-dienes in moderate to good yields and high selectivities (>20 : 1, **3***vs.***4**). Substrates with electron-donating groups proceed smoothly to deliver 1,5-dienes **3b–3d**. Fluoro and chloro groups are also well tolerated, yielding products **3e** and **3f** in 74% and 78% yield. Aryl alkynes bearing electron-withdrawing substituents (CF_3_, Ph, Ac, and CO_2_Me) show slightly higher reactivities, providing linear products (**3g–3j**) in high yields (81–90%). High selectivities are still obtained for *meta*-substituted 1-arylalkyne **1k–1m**. The steric hindrance of alkyne influences the reactivity and offers **3n** with 52% yield. On replacing the phenyl group with 2-naphthalenyl groups, the substrate transforms into a mixture of linear product **3o** and branched product **3o′** in a 1 : 1.7 ratio. The branched product **3o′** probably originates from the formed η^3^-π-benzyl-palladium intermediate.^15^ No desired 1,5-diene product is obtained when alkyl-substituted alkyne **1p** or **1q** is subjected to standard conditions. Instead, the isomerization product 1-phenyl-1,3-butadiene is observed for the conversion of **1p**. Notably, substrates bearing pyridine rings, which were incompatible in previously reported palladium catalysis,^16^ also lead to **3r** and **3s** in moderate yields. Finally, the late-stage modification of the estrone derivative **1t** affords **3t** in 38% yield. This 1,5-diene synthesis complements known allyl–allyl couplings that require pre-functionalized allyl precursors such as allyl chlorides or carbonates.^16^,^17^

**Table 1:** Regioselective hydroallylation of alkynes^*a*^

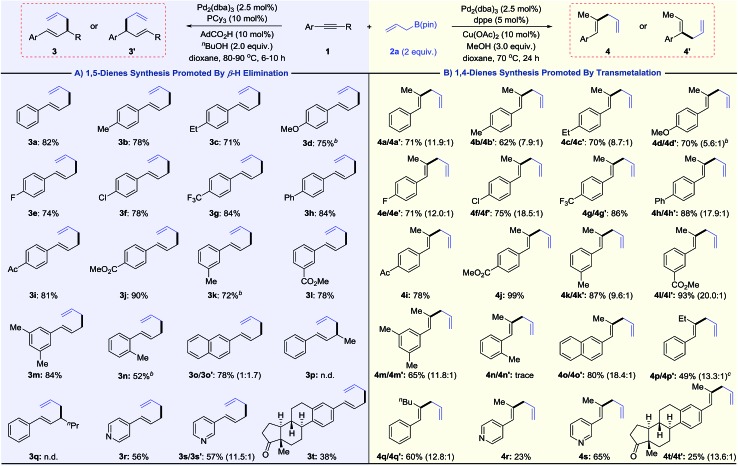

^*a*^Isolated yield of the all isomers. Unless otherwise noted, selectivity > 20 : 1.

^*b*^Accompanied by a small amount of inseparable alkyne **1**, and the yield of the product has been adjusted accordingly.

^*c*^Isolated product together with 1-phenyl-1,3-butadiene in a 4.0 : 1 ratio, and the yield of the product has been adjusted accordingly.

Then, we examined the substrate scope for the synthesis of 1,4-dienes (Table 1B). Various alkynes can be transformed into 1,4-dienes using the Pd/Cu catalyst combo. Although substrates bearing electron-donating groups lead to skipped dienes with moderate to good regioselectivities (**4b–4d**, **4k**, and **4m**), electron-withdrawing substrates perform well in terms of yields and selectivities (**4e–4j**, **4l**). Ortho-substituted alkyne **1n** exhibits no reactivity due to steric hindrance. It is noteworthy that **4p** and **4p′** are successfully acquired in 49% yield, accompanied by a small amount of isomerization side product (1-phenyl-1,3-butadiene). Comparatively, the cross-coupling between 1-phenyl-1-hexyne **1q** and **2a** provides 1,4-diene products **4q**/**4q′** without any 1,3-diene side product. Heterocyclic substituted alkynes (**1r** and **1s**) and estrone derivatives all successfully deliver the 1,4-diene products.

**Scheme 2:**
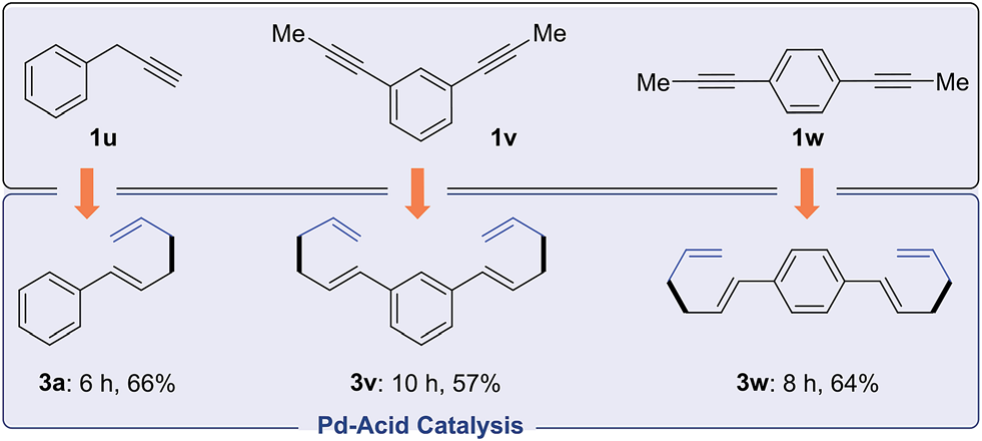
Hydroallylation of terminal and bis-alkynes.

Besides internal alkynes, terminal alkyne **1u** couples with allylB(pin) **2a** to yield 1,5-diene **3a** (Scheme 2). The bis-allylations of di(prop-1-yn-1-yl)benzene **1v** and **1w** proceed smoothly with high selectivities and moderate yields. These olefin products are potential monomers for polymerization.^18^

We also tested the scope of allylborons under our Pd-acid conditions (Table 2). Generally, substrates **2b–2d** were less reactive in allyl–allyl couplings. This agrees with previous work reported by Morken's group that substituted allylborons were comparatively reluctant in Pd-catalyzed allyl–allyl coupling reactions.17*g* To improve the reactivity, Cu(OAc)_2_ was employed as an additional promoter to facilitate transmetalation. The yields of **3x** and **3y** were successfully increased to 34% and 38%, respectively. It should be noted that these reactions all give interesting linear-branched coupling products, and this selectivity is rare in Suzuki-type allyl–allyl coupling reactions.^17^

**Table 2:** The scope of allylborons

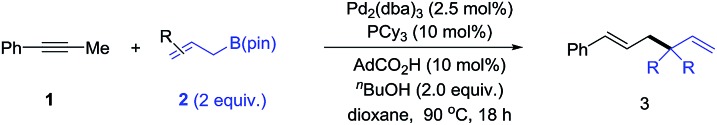			
**2**	Product	Yield (%)	
		w/o Cu(OAc)_2_	Cu(OAc)_2_
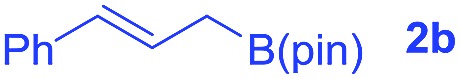	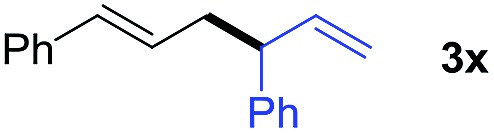	6	34^*a*^
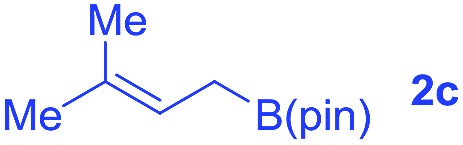	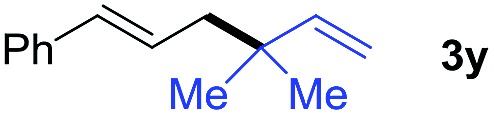	n.d.	<5^*a*^
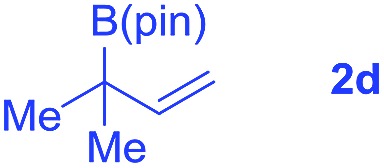		13	38^*a*^

^*a*^Cu(OAc)_2_ (10 mol%).

**Fig. 1:**
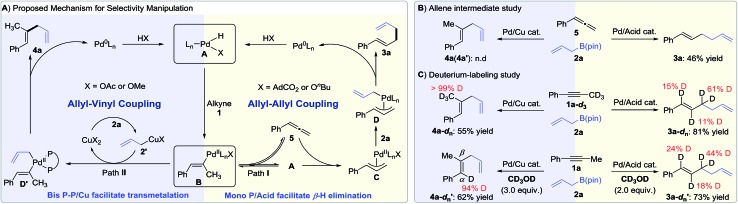
Divergent reactivity of Pd(ii) species.

While further studies are warranted, we propose the following mechanisms on the basis of literature^7^ and our own observations (Fig. 1A, right). First, the oxidative addition of the carboxylic acid with a Pd(0) precursor generates Pd(ii)-hydride species **A**. *Syn*-Migratory insertion of alkyne **1** into Pd(ii)–H **A** affords vinyl-Pd intermediate **B**. For the 1,5-diene pathway, a vacant coordination site of complex **B** is spared for β-hydride elimination in the presence of the monophosphine ligand. Allene **5** is subsequently produced and undergoes reinsertion into Pd(ii)–H forming the π-allyl-Pd intermediate **C**. Then, electrophilic intermediate **C** reacts with allylB(pin) **2** to deliver bis(allyl)Pd species **D**.^19^ Reductive elimination yields the allyl–allyl coupling product **3a** and turns over the Pd(0) catalyst (allyl–allyl coupling cycle).

In the presence of a Cu(ii) co-catalyst, we propose that transmetalation of allylboron **2a** to generate allylcopper species **2′** is favored.5i,17k In this protocol, vinyl-Pd species **B** is also generated by a *syn*-migratory of alkyne **1** into Pd(ii)–H **A** (Fig. 1A, left). However, when coordinated with a bidentate ligand, vinyl-Pd intermediate **B** prefers direct transmetalation with allylcopper species **2′** to form **D′** rather than undergoing β-hydride elimination to produce allene **5**. Reductive elimination from **D′** yields the product 1,4-diene **4a** (allyl–vinyl coupling cycle).

To probe the feasibility of an allene intermediate, phenylallene **5** was subjected to couple with allyl-B(pin) **2a** under two standard conditions (Fig. 1B, see the ESI for details†). The allene was transformed into 1,5-diene **3a** in 46% yield. No formation of **4a** or **4a′** under the Pd–Cu conditions supports that the 1,4-diene products do not arise from the addition of allylB(pin) **2a** to allene **5**. Hydroallylation was also performed with deuterated alkyne **1a**-*d*_3_ or methanol (Fig. 1C). Under Pd/acid catalysis, we found that the deuterium label was scrambled into the α-, β-, and γ-positions of 1,5-diene **3a**-*d*_*n*_ using **1a**-*d*_3_. Similar deuterium scrambling was observed when conducting reaction with deuterated methanol as a proton source. This observation supports a reversible hydrometallation of the internal π-system of the allene in the synthesis of 1,5-dienes. When experiments were carried out under Pd–Cu catalysis, the deuterium label remained intact in 1,4-diene **4a**-*d*_*n*_ with deuterated alkyne **1a**-*d*_3_. Only an α-deuterated product was achieved using deuterated methanol (Fig. 1C). This indicates that β-hydride elimination is not involved in Pd/Cu catalysis.

## Conclusions

Our work complements other alkyne hydroallylation methods for the synthesis of 1,4-dienes including those developed by Hilt, Hartwig, Lalic and Zhang.^5^ The key to the success of this method is the switchable reactivity of vinyl-Pd intermediates. Acid additive promotes the β-hydride elimination pathway for allyl–allyl coupling with the aid of a monophosphine ligand, whereas the Cu co-catalyst facilitates the direct transmetalation for vinyl-allyl coupling in the presence of a bisphosphine ligand. Transmetalation and β-hydride elimination are two elementary steps featured in many well-known organometallic mechanisms, including Suzuki–Miyaura and Heck cross-coupling. Insights from this study will guide the future development of related regiodivergent methods in catalysis.

## Conflicts of interest

There are no conflicts to declare.

## Supplementary Material

Supplementary information
