# Supplementary material for: A regioselectivity switch in Pd-catalyzed hydroallylation of alkynes
Source: Chem Sci. 2019 May 16;10(25):6311–5. doi: 10.1039/c9sc01527b (PMC6598511; doi:10.1039/c9sc01527b)
Supplement: Supplementary file 1 [file SC-010-C9SC01527B-s001.pdf]

Electronic Supplementary Material (ESI) for Chemical Science.
This journal is © The Royal Society of Chemistry 2019

Supporting Information For:

Regioselectivity Switch in Pd-Catalyzed Hydroallylation of Alkynes

Ding-Wei Ji[†,‡], Yan-Cheng Hu[†], Hao Zheng[†,‡], Chao-Yang Zhao[†,‡], Qing-An Chen[*,†] and Vy M. Dong[§]

[†]Dalian Institute of Chemical Physics, Chinese Academy of Sciences, Dalian 116023, China

[‡]University of Chinese Academy of Sciences, Beijing 100049, China

[§]University of California, Irvine, 4403 Natural Sciences 1, Irvine, CA 92697, United States

Table of Contents

1. General experimental details........S1
2. Typical procedure for the preparation of alkynes........S1
3. Screening of reaction conditions........S2-3
4. Typical procedure for palladium-catalyzed allyl-allyl coupling reaction........S4-8
5. Typical procedure for the synthesis of **3u** and **3w**........S9
6. Typical procedure for palladium-catalyzed allyl-alkenyl coupling reaction........S9-13
7. Control and deuterium labeling experiments........S14
8. References........S15
9. Copy of NMR for products........S16-62

1. General experimental details: $Pd_2(dba)_3$ was purchased from Adamas. Phenyl allene was prepared by following the literature report.[1] All other reagents were commercially available and were used without further purification unless otherwise stated. Solvents were treated prior to use according to the standard methods. ^1H NMR and ^{13}C NMR spectra were recorded at room temperature in $CDCl_3$ on 400 MHz instrument with tetramethylsilane (TMS) as internal standard. Data are reported as follows: chemical shift in ppm (δ), multiplicity (s = singlet, d = doublet, t = triplet, q = quartet, brs = broad singlet, m = multiplet), coupling constant (Hz), and integration. Flash column chromatography was performed on commercially available silica gel (200-300 mesh). All reactions were monitored by TLC, GC-FID or NMR analysis.

2. Typical procedure for the preparation of alkynes

Substrates **1a**, **1p**, **1q** and **1u** are commercially available, and **1b-1o** and **1t** was synthesized according to the literature report.[2] Substrates **1r-1s**, **1v-1w** and **1a-*d*$_3$** were synthesized according to the following procedures.

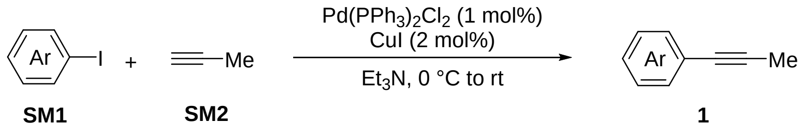

To a flame-dried round-bottom flask under N_2, substituted iodobenzene **SM1** (5 mmol, 1.0 equiv), $PdCl_2(PPh_3)_2$ (1 mol%) and CuI (2 mol%) were dissolved in 5 mL Et_3N and the reaction mixture was stirred under N_2 at 0 ^oC for 5 min. Propyne (6 mmol, 1.2 equiv, ca. 1.0 M in tetrahydrofuran, purchased from TCI) was added in one portion and the solution was stirred at room temperature for 5~10 h. After the starting material was consumed completely which was detected by TLC, the reaction mixture was diluted with 20 mL dichloromethane, then washed with saturated NaCl aqueous solution, dried over anhydrous Na_2SO_4. The mixture was concentrated in vacuo and purified by flash chromatography on silica gel with *n*-pentene to afford the alkynylation products **1r** and **1s**.

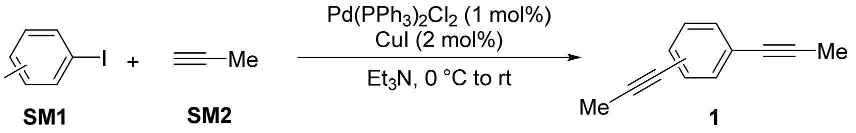

To a flame-dried round-bottom flask under N_2, substituted iodobenzene **SM1** (5 mmol, 1.0 equiv), $PdCl_2(PPh_3)_2$ (1 mol%) and CuI (2 mol%) were dissolved in 5 mL Et_3N and the reaction mixture was stirred under N_2 at 0 ^oC for 5 min. Propyne (12 mmol, 2.4 equiv, ca. 1.0 M in tetrahydrofuran, purchased from TCI) was added in one portion and the solution was stirred at room temperature for 5~10 h. After the starting material was consumed completely which was detected by TLC, the reaction mixture was diluted with 20 mL dichloromethane, then washed with saturated NaCl aqueous solution, dried over anhydrous Na_2SO_4. The mixture was concentrated in vacuo and purified by flash chromatography on silica gel with petroleum ether to afford the alkynylation products **1v** and **1w**.

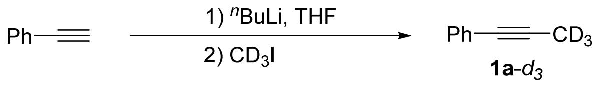

To a flame-dried round-bottom flask under N_2, phenylacetylene (9 mmol, 1.0 equiv) were dissolved in 20 mL THF and the flask cooled to -20 ^oC. ^{n}BuLi (18 mmol, 2.0 equiv) was added slowly and the reaction mixture was allowed to stir at room for 1 h. Then CD_3I (19 mmol, 2.1 equiv) was added at -20 ^oC and the reaction was allowed to stir at room temperature for another 1 h. After the starting material was consumed completely which was detected by TLC, the reaction was quenched with a saturated aqueous solution of ammonium chloride, extracted with dichloromethane, washed with saturated NaCl aqueous solution and dried over anhydrous Na_2SO_4. The mixture was concentrated in vacuo and purified by flash chromatography on silica gel with *n*-pentene to afford the product **1a**-*d*$_3$.

3. Screening of reaction conditions

Table S1. Screening of the conditions for 3a

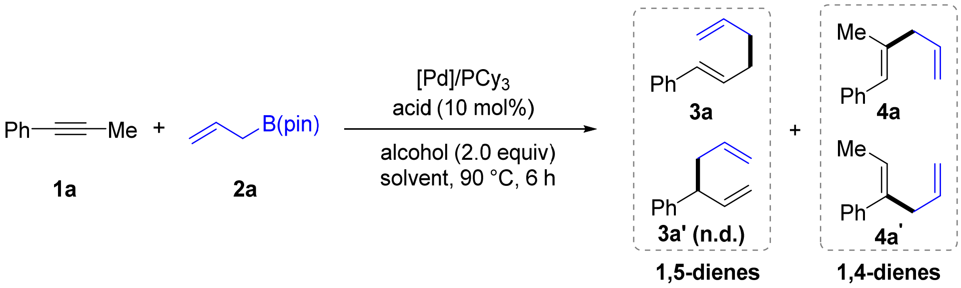

entry	"Pd"	acid	alcohol	solvent	yield (%)[b]	
					**3a**	**others**
1	$Pd_2(dba)_3$	$(PhO)_2PO_2H$	^{n}BuOH	dioxane	trace	trace
2	$Pd_2(dba)_3$	$PhSO_3H$	^{n}BuOH	dioxane	trace	trace
3	$Pd_2(dba)_3$	$PhCO_2H$	^{n}BuOH	dioxane	60	trace
4	$Pd_2(dba)_3$	AcOH	^{n}BuOH	dioxane	78	trace
5	$Pd_2(dba)_3$	$AdCO_2H$	^{n}BuOH	dioxane	83	trace
6	$Pd_2(dba)_3$	PivOH	^{n}BuOH	dioxane	76	trace
7	$Pd_2(dba)_3$	$AdCO_2H$	MeOH	dioxane	83	3
8	$Pd_2(dba)_3$	$AdCO_2H$	EtOH	dioxane	82	trace
9	$Pd_2(dba)_3$	$AdCO_2H$	^{t}BuOH	dioxane	68	trace
10[c]	$Pd_2(dba)_3$	$AdCO_2H$	^{n}BuOH	dioxane	54	trace
11[d]	$Pd_2(dba)_3$	$AdCO_2H$	^{n}BuOH	dioxane	36	trace
12	$Pd_2(dba)_3$	$AdCO_2H$	^{n}BuOH	THF	73	trace
13	$Pd_2(dba)_3$	$AdCO_2H$	^{n}BuOH	DCE	trace	trace
14	$Pd_2(dba)_3$	$AdCO_2H$	^{n}BuOH	toluene	57	trace
15	$Pd_2(dba)_3$	$AdCO_2H$	^{n}BuOH	MeCN	25	8
16	$Pd_2(dba)_3$	$AdCO_2H$	^{n}BuOH	EtOAc	44	trace
17	$Pd(OAc)_2$	$AdCO_2H$	^{n}BuOH	dioxane	40	trace
18	$PdCl_2$	$AdCO_2H$	^{n}BuOH	dioxane	trace	trace

[a]Reaction conditions: **1a** (0.20 mmol), **2a** (0.40 mmol), [Pd] (5 mol%), PCy_3 (10 mol%), acid (10 mol%), alcohol (2.0 equiv), solvent (1.0 mL), 90 ˚C, 6 h. [b]Determined by GC using 1,3,5-trimethoxy-benzene as internal standard. [c]110 ˚C. [d]70 ˚C.

Table S2. Screening of the conditions for 4a

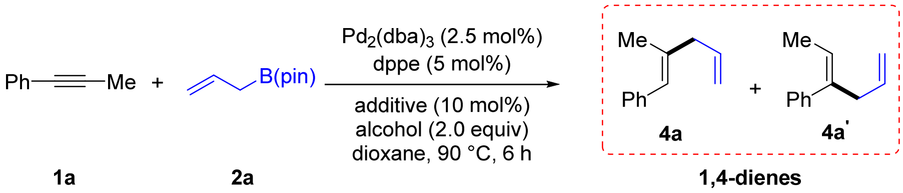

entry	additive	alcohol (x equiv)	yield (%)[b]	
			**4a**	**4a'**
1	$Co(OAc)_2$•$4H_2O$	^{n}BuOH (2.0)	trace	trace
2	$Zn(OAc)_2$•$2H_2O$	^{n}BuOH (2.0)	trace	trace
3	$Ni(OAc)_2$	^{n}BuOH (2.0)	trace	trace
4	$Fe(OAc)_2$	^{n}BuOH (2.0)	trace	trace
5	AgOAc	^{n}BuOH (2.0)	trace	trace
6	NaOAc	^{n}BuOH (2.0)	trace	trace
7	$Cu(OAc)_2$	^{n}BuOH (2.0)	53	5
8	$Cu(OAc)_2$	MeOH (2.0)	55	5
9	$Cu(OAc)_2$	MeOH (3.0)	57	6
10[c]	$Cu(OAc)_2$	MeOH (3.0)	66	6
11[c,d]	$Cu(OAc)_2$	MeOH (3.0)	76	7
12[c,d,e]	$Cu(OAc)_2$	MeOH (3.0)	79(**65**[g])	7(**6**[g])
13[c,d,e,f]	$Cu(OAc)_2$	MeOH (3.0)	n.d.	n.d.
14[c,d,e,f]	CuOAc	MeOH (3.0)	63	6

[a]Reaction conditions: **1a** (0.20 mmol), **2** (0.40 mmol), $Pd_2(dba)_3$ (2.5 mol%), dppe (5 mol%), additive (10 mol%), dioxane (1.0 mL), 90 ˚C, 6 h. [b]Determined by GC using 1,3,5-trimethoxy-benzene as internal standard. n.d. = no detected. Unless otherwise noted, regioselectivity > 20:1. [c] Dioxane (0.5 mL). [d]70 ˚C. [e]24 h. [f]In the absence of $Pd_2(dba)_3$. [g]Isolated yield.

4. Typical procedure for palladium-catalyzed allyl-allyl coupling reaction

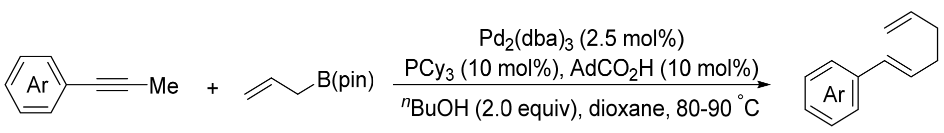

1 **2a** **3**

In glove box, a sealed tube was charged with alkynes **1** (0.2 mmol, 1.0 equiv), **2a** (0.4 mmol, 2.0 equiv), $Pd_2(dba)_3$ (0.005 mmol, 2.5 mol%), PCy_3 (0.02 mmol, 10 mol%), $AdCO_2H$ (0.02 mmol, 10 mol%) and ^{n}BuOH (0.4 mmol, 2.0 equiv) in dioxane (1.0 mL). The reaction tube was sealed with a Teflon screw cap, removed from the glove box. Then, the reaction mixture was stirred at 80-90 ^{o}C for 6-10 h. After cooling to room temperature, the reaction mixture was directly purified by column chromatography on silica gel using *n*-pentene to afford the corresponding product **3**.

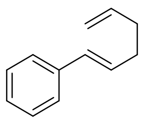

(*E*)-1-(hexa-1,5-dien-1-yl)-benzene (3a): Prepared according to the general procedure, 90 ^{o}C, 6 h, 82% yield, known compound,[3] colorless oil, R_f = 0.8 (Petroleum ether), **^{1}H NMR** (400 MHz, $CDCl_3$) δ 7.36-7.33 (m, 2H), 7.31-7.27 (m, 2H), 7.21-7.17 (m, 1H), 6.40 (d, *J* = 15.8, 1H), 6.23 (dt, *J* = 15.8, 6.6 Hz 1H), 5.87 (ddt, *J* = 17.0, 10.3, 6.4 Hz, 1H), 5.06 (dt, *J* = 17.1, 1.7 Hz, 1H), 5.00 (dt, *J* = 10.2, 1.6 Hz, 1H), 2.35-2.29 (m, 2H), 2.27-2.20 (m, 2H); **^{13}C NMR** (100 MHz, $CDCl_3$) δ 138.11, 137.77, 130.21,

130.13, 128.49, 126.90, 125.98, 114.94, 33.58, 32.45.

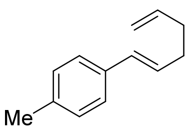

(*E*)-1-(hexa-1,5-dien-1-yl)-4-methylbenzene (3b): Prepared according to the general procedure, 90 ^oC, 6 h, 78% yield, known compound,[4] colorless oil, R_f = 0.8 (Petroleum ether). **^{1}H NMR** (400 MHz, $CDCl_3$) δ 7.23 (d, *J* = 8.1, 2H), 7.09 (d, *J* = 7.8, 2H), 6.36 (d, *J* = 15.8, 1H), 6.16 (dt, *J* = 15.8, 6.6 Hz, 1H), 5.86 (ddt, *J* = 17.0, 10.3, 6.3 Hz 1H), 5.07 (dq, *J* = 17.2, 1.6 Hz, 1H), 4.98 (ddt, *J* =10.2, 2.1, 1.2 Hz, 1H), 2.31 (s, 3H), 2.31-2.27 (m, 2H), 2.25-2.19 (m, 2H); **^{13}C NMR** (100 MHz, $CDCl_3$) δ 138.20, 136.59, 135.00, 130.03, 129.19, 129.08, 125.87, 114.87, 33.66, 32.46, 21.15.

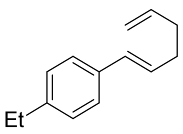

(*E*)-1-(hexa-1,5-dien-1-yl)-4-ethylbenzene (3c): Prepared according to the general procedure, 90 ^oC, 10 h, 71% yield, colorless oil, R_f = 0.8 (Petroleum ether), **^{1}H NMR** (400 MHz, $CDCl_3$) δ 7.26 (d, *J* = 8.2 Hz, 2H), 7.12 (d, *J* = 8.1 Hz, 2H), 6.38 (dt, *J* = 15.8, 1.4 Hz, 1H), 6.17 (dt, *J* = 15.8, 6.6 Hz, 1H), 5.86 (ddt, *J* = 17.0, 10.3, 6.4 Hz, 1H), 5.06 (dq, *J* = 17.2, 1.6 Hz, 1H), 4.98 (ddt, *J* = 10.2, 2.1, 1.2 Hz, 1H) 2.62 (q, *J* = 7.6 Hz, 2 H), 2.33-2.27 (m, 2H), 2.25-2.19 (m, 2H), 1.22 (t, *J* = 7.6 Hz, 3H); **^{13}C NMR** (100 MHz, $CDCl_3$) δ 143.05, 138.19, 135.26, 130.05, 129.16, 127.99, 125.94, 114.86, 33.66, 32.46, 28.58, 15.63. **HRMS** Calculated for $C_{14}H_{18}$ $[M+H]^+$ 187.1481, found 187.1489.

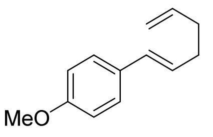

(*E*)-1-(hexa-1,5-dien-1-yl)-4-methoxybenzene (3d): Prepared according to the general procedure, 80 ^oC, 10 h, 75% yield, known compound,[3] pale yellow oil, R_f = 0.6 (petroleum ether). **^{1}H NMR** (400 MHz, $CDCl_3$) δ 7.26 (d, *J* = 8.7 Hz, 2H), 6.83 (d, *J* = 8.7 Hz, 2H), 6.34 (d, *J* = 15.8 Hz, 1H), 6.07 (dt, *J* = 15.8, 6.6 Hz, 1H), 5.86 (ddt, *J* = 16.9, 10.1, 6.3 Hz, 1H), 5.05 (dq, *J* = 17.1, 1.7 Hz, 1H), 4.98 (dd, *J* = 10.2, 1.9 Hz, 1H); **^{13}C NMR** (100 MHz, $CDCl_3$) δ 158.73, 138.22, 130.64, 129.55, 127.95, 127.04, 114.83, 113.93, 55.28, 33.72, 32.43.

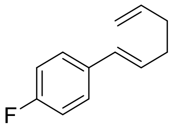

(*E*)-1-(hexa-1,5-dien-1-yl)-4-fluorobenzene (3e): Prepared according to the general procedure, 90 ^oC, 6 h, 74% yield, colorless oil, R_f = 0.7 (Petroleum ether), **^{1}H NMR** (400 MHz, $CDCl_3$) δ 7.31-7.26 (m, 2H), 7.00-6.94 (m, 2H), 6.36 (d, *J* = 15.9 Hz, 1H), 6.13 (dt, *J* = 15.8, 6.6 Hz, 1H), 5.86 (ddt, *J* = 17.0, 10.1, 6.3 Hz, 1H), 5.06 (dq, *J* = 17.1, 1.7 Hz, 1H), 5.00 (dq, *J* = 10.1, 1.6 Hz, 1H), 2.33-2.27 (m, 2H), 2.25-2.20 (m, 2H); **^{13}C NMR** (100 MHz, $CDCl_3$) δ 161.93 (d, *J* = 244.2 Hz), 138.02, 133.91 (d, *J* = 3.2 Hz), 129.84 (d, *J* = 2.2 Hz), 129.03, 127.4 (d, *J* = 7.7 Hz), 115.20 (d, *J* = 21.4 Hz), 114.97, 33.52, 32.34; **^{19}F NMR** (376 MHz, $CDCl_3$) δ -115.75. **HRMS** Calculated for $C_{12}H_{13}F$ 176.1001, found 176.1010.

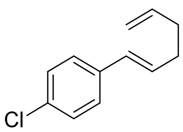

(*E*)-1-(hexa-1,5-dien-1-yl)-4-chlorobenzene (3f): Prepared according to the general procedure, 90 ^oC, 6 h, 78% yield, known compound,[4] colorless oil, R_f = 0.7 (Petroleum ether), **^{1}H NMR** (400 MHz, $CDCl_3$) δ 7.25-7.24 (m, 4H), 6.34 (d, *J* = 15.8 Hz, 1H), 6.19 (dt, *J* = 15.8, 6.5 Hz, 1H), 5.85 (ddt, *J* = 17.0, 10.4, 6.4 Hz, 1H), 5.08-5.03 (m, 1H), 5.02-4.98 (m, 1H), 2.33-2.27 (m, 2H), 2.25-2.19 (m, 2H); **^{13}C NMR** (100 MHz, $CDCl_3$) δ 137.94, 136.25, 132.43, 130.86, 129.06, 128.60, 127.17, 115.06, 33.44, 32.38.

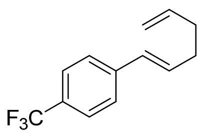

(*E*)-1-(hexa-1,5-dien-1-yl)-4-(trifluoromethyl)benzene (3g): Prepared according to the general procedure, 90 °C, 6 h, 84% yield, known compound,[3] colorless oil, R_f = 0.4 (Petroleum ether), **^{1}H NMR** (400 MHz, $CDCl_3$) δ 7.53 (d, J = 8.2 Hz, 2H), 7.41 (d, J = 8.1 Hz, 2H), 6.43 (d, J = 15.9 Hz, 1H), 6.33 (dt, J = 15.9, 6.3 Hz, 1H), 5.85 (ddt, J = 17.0, 10.3, 6.4 Hz, 1H), 5.07 (dq, J = 17.1, 1.7 Hz, 1H), 5.01 (ddt, J = 10.2, 1.9, 1.3 Hz, 1H), 2.37-2.31 (m, 2H), 2.28-2.22 (m, 2H); **^{13}C NMR** (100 MHz, $CDCl_3$) δ 141.22, 137.77, 132.97, 129.06, 128.71 (q, J = 32.2 Hz), 126.07, 125.42 (q, J = 3.8 Hz), 124.30 (q, J = 269.9 Hz), 115.17, 33.28, 32.39; **^{19}F NMR** (376 MHz, $CDCl_3$) δ -62.41.

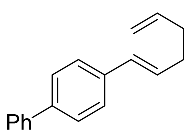

(*E*)-4-(hexa-1,5-dien-1-yl)-1,1'-biphenyl (3h): Prepared according to the general procedure, 90 °C, 6 h, 84% yield, known compound,[4] colorless solid, R_f = 0.6 (Petroleum ether), **^{1}H NMR** (400 MHz, $CDCl_3$) δ 7.59-7.57 (m, 2H), 7.54-7.51 (m, 2H), 7.44-7.39 (m, 4H), 7.34-7.30 (m, 1H), 6.43 (d, J = 15.9 Hz, 1H), 6.27 (dt, J =15.8, 6.6 Hz, 1H), 5.87 (ddt, J =17.0, 10.2, 6.4, 1H), 5.07 (dq, J = 17.1, 1.6 Hz, 1H), 5.00 (ddt, J = 10.2, 2.0, 1.2 Hz, 1H), 2.36-2.30 (m, 2H), 2.28-2.21 (m, 2H); **^{13}C NMR** (100 MHz, $CDCl_3$) δ 141.18, 139.98, 138.41, 137.15, 130.64, 130.10, 129.08, 127.52, 127.49, 127.22, 126.71, 115.30, 33.90, 32.84.

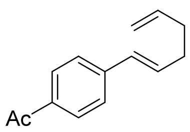

(*E*)-1-(hexa-1,5-dien-1-yl)-4-acetylbenzene (3i): Prepared according to the general procedure, 90 °C, 6 h, 81% yield, pale yellow oil, R_f = 0.4 (Petroleum ether/ethyl acetate 50/1), **^{1}H NMR** (400 MHz, $CDCl_3$) δ 7.90-7.87 (m, 2H), 7.42-7.39 (m, 2H), 6.44 (d, J = 16.1 Hz, 1H), 6.37 (dt, J = 15.8, 6.1 Hz, 1H), 5.86 (ddt, J = 17.0, 10.2, 6.5 Hz, 1H), 5.07 (dq, J = 17.1, 1.6 Hz, 1H), 5.01 (ddt, J = 10.2, 2.1, 1.2 Hz, 1H), 2.58 (s, 3H), 2.38-2.32 (m, 2H), 2.28-2.22 (m, 2H); **^{13}C NMR** (100 MHz, $CDCl_3$) δ 197.58, 142.46, 137.77, 135.51, 133.41, 129.42, 128.74, 125.98, 115.17, 33.26, 32.50, 26.55. **HRMS** Calculated for $C_{14}H_{17}O$ $[M+H]^+$ 201.1274, found 201.1271.

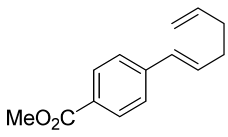

Methyl (*E*)-4-(hexa-1,5-dien-1-yl)benzoate (3j): Prepared according to the general procedure, 90 °C, 6 h, 90% yield, colorless oil, R_f = 0.6 (Petroleum ether/ethyl acetate 20/1), **^{1}H NMR** (400 MHz, $CDCl_3$) δ 7.95 (d, J = 8.4 Hz, 2H), 7.38 (d, J = 8.3 Hz, 2H), 6.44 (d, J = 15.9 Hz, 1H), 6.35 (dt, J = 15.8, 6.2 Hz, 1H), 5.86 (ddt, J = 16.9, 10.2, 6.5 Hz, 1H), 5.07 (dq, J = 17.1, 1.6, 1H), 5.01 (ddt, J = 10.2, 2.2, 1.2 Hz, 1H), 3.90 (s, 3H), 3.37-2.31 (m, 2H), 2.28-2.21 (m, 2H); **^{13}C NMR** (100 MHz, $CDCl_3$) δ 167.29, 142.58, 138.13, 133.40, 130.20, 129.82, 128.69, 126.13, 115.46, 52.31, 33.60, 32.80. **HRMS** Calculated for $C_{14}H_{17}O_2$ $[M+H]^+$ 217.1223, found 217.1225.

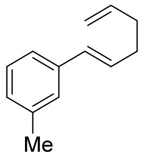

(*E*)-1-(hexa-1,5-dien-1-yl)-3-methylbenzene (3k): Prepared according to the general procedure, 90 °C, 10 h, 72% yield, known compound,[4] colorless oil, R_f = 0.7 (petroleum ether). **^{1}H NMR** (400 MHz, $CDCl_3$) δ 7.20-7.12 (m, 3H), 7.00 (dt, J = 7.2, 1.6 Hz, 1H), 6.37 (dt, J = 15.8, 1.4 Hz, 1H), 6.21 (dt, J = 15.8, 6.6 Hz, 1H), 5.86 (ddt, J = 17.0, 10.2, 6.4 Hz 1H), 5.06 (dq, J = 17.1, 1.6 Hz, 1H), 4.99 (ddt, J = 10.2, 2.2, 1.2, 1H), 2.33 (s, 3H), 2.32-2.27 (m, 2H), 2.25-2.19 (m, 2H); **^{13}C NMR** (100 MHz, $CDCl_3$) δ 138.15, 138.00, 137.71, 130.27, 129.91, 128.39, 127.69, 126.70, 123.12, 114.90, 33.60, 32.47, 21.42.

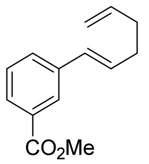

Methyl (*E*)-3-(hexa-1,5-dien-1-yl)benzoate (3l): Prepared according to the general procedure, 90 ^{o}C, 8 h, 78% yield, colorless oil, R_f = 0.6 (petroleum ether/EtOAc 20/1). **^{1}H NMR** (400 MHz, $CDCl_3$) δ 8.01 (s, 1H), 7.86 (dt, *J* = 7.7, 1.5 Hz, 1H), 7.51 (dt, *J* = 7.7, 1.5 Hz, 1H), 7.35 (t, *J* = 7.7, 1H), 6.43 (d, *J* = 15.8 Hz, 1H), 6.31 (dt, *J* = 17.0, 6.6 Hz, 1H); 5.86 (ddt, *J* = 16.6, 10.2, 6.4 Hz, 1H), 5.07 (dq, *J* = 17.1, 1.6 Hz, 1H), 5.00 (ddt, *J* = 10.3, 2.2, 1.2 Hz, 1H), 3.91 (s, 3H), 2.36-2.30 (m, 2H), 2.27-2.21 (m, 2H) ; **^{13}C NMR** (100 MHz, $CDCl_3$) δ 167.14, 138.07, 137.91, 131.51, 130.38, 130.34, 129.30, 128.51, 127.89, 127.03, 115.06, 52.11, 33.38, 32.39. **HRMS** Calculated for $C_{14}H_{17}O_2$ $[M+H]^+$ 217.1223, found 217.1225.

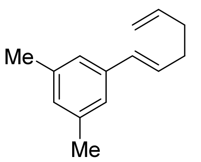

(*E*)-1-(hexa-1,5-dien-1-yl)-3,5-dimethylbenzene (3m): Prepared according to the general procedure, 90 ^{o}C, 10 h, 84% yield, colorless oil, R_f = 0.7 (petroleum ether). **^{1}H NMR** (400 MHz, $CDCl_3$) δ 6.96-6.95 (m, 2H), 6.84-6.83 (m, 2H), 6.33 (dt, *J* = 15.7, 1.4 Hz, 1H), 6.19 (dt, *J* = 15.8, 6.5 Hz, 1H), 5.86 (ddt, *J* = 17.0, 10.1, 6.3 Hz, 1H), 5.05 (dq, *J* = 17.1, 1.7 Hz, 1H), 4.99 (ddt, *J* = 10.2, 2.2, 1.2 Hz, 1H), 2.32-2.26 (m, 2H), 2.29 (s, 6H), 2.25-2.18 (m, 2H); **^{13}C NMR** (100 MHz, $CDCl_3$) δ 138.19, 137.91, 137.69, 130.33, 129.72, 128.64, 123.89, 114.87, 33.64, 32.50, 21.29. **HRMS** Calculated for $C_{14}H_{18}$ 186.1409, found 186.1410.

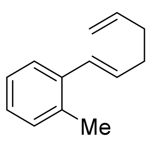

(*E*)-1-(hexa-1,5-dien-1-yl)-2-methylbenzene (3n): Prepared according to the general procedure, 90 ^{o}C, 6 h, 52% yield, known compound,[4] colorless oil, R_f = 0.6 (petroleum ether). **^{1}H NMR** (400 MHz, $CDCl_3$) δ 7.41-7.39 (m, 1H), 7.16-7.10 (m, 3H), 6.59 (dt, *J* = 15.6, 1.5 Hz, 1H), 6.09 (dt, *J* = 15.7, 6.7 Hz, 1H), 5.87 (ddt, *J* = 17.0, 10.2, 6.4 Hz, 1H), 5.09-5.04 (m, 1H), 5.00 (ddd, *J* = 10.2, 2.1, 1.0 Hz, 1H), 2.36-2.31 (m, 2H), 2.33 (s, 3H), 2.27-2.22 (m, 2H); **^{13}C NMR** (100 MHz, $CDCl_3$) δ 138.14, 136.92, 134.94, 131.47, 130.14, 128.10, 126.84, 126.00, 125.49, 114.92, 33.67, 32.70, 19.85.

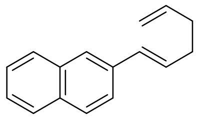

1,4-di((E)-hexa-1,5-dien-1-yl)benzene (3o): Prepared according to the general procedure, 90 ^{o}C, 6 h, 78% total yield, the ratio of **3n**/**3n'** is 1:1.7, known compound,[4] colorless oil, R_f = 0.6 (petroleum ether). **^{1}H NMR** (400 MHz, $CDCl_3$) δ 7.82-7.78 (m, 3H), 7.69 (s, 1H), 7.60-7.58 (m, 1H), 7.47-7.41 (m, 2H), 6.58 (d, *J* = 15.9 Hz, 1H), 6.38 (dt, *J* = 15.8, 6.7 Hz, 1H), 5.91 (ddt, *J* = 17.0, 10.2, 6.5 Hz, 1H), 5.13-5.04 (m, 2H), 2.42-2.36 (m, 2H), 2.33-2.26 (m, 2H).

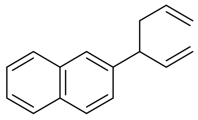

2-(hexa-1,5-dien-3-yl)naphthalene (3o'): Prepared according to the general procedure, 90 ^{o}C, 6 h, 78% total yield, the ratio of **3n**/**3n'** is 1:1.7, known compound,[5] colorless oil, R_f = 0.6 (petroleum ether). **^{1}H NMR** (400 MHz, $CDCl_3$) δ 7.83-7.76 (m, 3H), 7.65 (s, 1H), 7.50-7.40 (m, 2H), 7.36 (dd, *J* = 8.5, 1.8 Hz, 1H), 6.08 (ddd, *J* = 17.3, 10.4, 7.2 Hz, 1H), 5.77 (ddt, *J* = 17.1, 10.1, 6.9 Hz, 1H), 5.12-4.95 (m, 4H), 3.55 (q, *J* = 7.4 Hz, 1H), 2.62-2.59 (m, 2H).

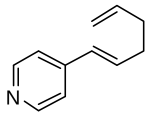

(*E*)-4-(hexa-1,5-dien-1-yl)pyridine (3r): Prepared according to the general procedure, 90 ^{o}C, 6 h, 56% yield, yellow oil, R_f = 0.3 (petroleum ether/EtOAc 5/1). **^{1}H NMR** (400 MHz, $CDCl_3$) δ 8.53-8.47 (m, 2H), 7.23-7.16 (m, 2H), 6.47 (dt, *J* =

15.9, 6.6 Hz, 1H), 6.34 (d, J = 15.9 Hz, 1H), 5.85 (ddt, J = 16.8, 10.1, 6.5 Hz, 1H), 5.07 (dq, J = 17.1, 1.6 Hz, 1H), 5.04-5.00 (m, 1H), 2.38-2.32 (m, 2H), 2.28-2.22 (m, 2H); ^{13}**C NMR** (100 MHz, $CDCl_3$) δ 150.02, 144.98, 137.56, 135.26, 128.18, 120.59, 115.31, 33.03, 32.33. **HRMS** Calculated for $C_{11}H_{14}N$ $[M+H]^+$ 160.1121, found 160.1138.

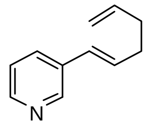

(*E*)-3-(hexa-1,5-dien-1-yl)pyridine (3s): Prepared according to the general procedure, 90 °C, 6 h, 57% total yield, the ratio of **3s**/**3s'** is 11.5:1, known compound,[6] colorless oil, R_f = 0.3 (petroleum ether/EtOAc 5/1). ^{1}**H NMR** (400 MHz, $CDCl_3$) δ 8.56 (d, J = 2.2 Hz, 1H), 8.43 (dd, J = 4.8, 1.6 Hz, 1H), 7.65 (dt, J = 7.9, 2.0 Hz, 1H), 7.21 (ddd, J = 8.0, 4.8, 0.8 Hz, 1H), 6.39 (d, J = 15.9 Hz, 1H), 6.30 (dt, J = 15.9, 6.3 Hz, 1H), 5.86 (ddt, J = 16.8, 10.2, 6.5 Hz, 1H), 5.07 (dq, J = 17.1, 1.7 Hz, 1H), 5.01 (ddt, J = 10.2, 2.1, 1.2 Hz, 1H), 2.37-2.31 (m, 2H), 2.28-2.22 (m, 2H). ^{13}**C NMR** (100 MHz, $CDCl_3$) δ 148.01, 148.00, 137.75, 133.25, 132.59, 132.41, 126.73, 123.34, 115.18, 33.28, 32.45.

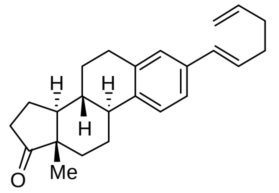

(8R,9S,13S,14S)-3-((*E*)-hexa-1,5-dien-1-yl)-13-methyl-6,7,8,9,11,12,13,14,15,16-decahydro-17H-cyclopenta[a]phenanthren-17-one (3t): Prepared according to the general procedure, 90 °C, 6 h, 56% yield, yellow solid, colorless solid, m.p. 80-82 °C, R_f = 0.3 (petroleum ether/EtOAc 20/1). ^{1}**H NMR** (400 MHz, $CDCl_3$) δ 7.22 (d, J = 8.2 Hz, 1H), 7.14 (dd, J = 8.1, 1.9 Hz, 1H), 7.07 (s, 1H), 6.35 (d, J = 15.9 Hz, 1H), 6.18 (dt, J = 15.8, 6.6 Hz, 1H), 5.86 (ddt, J = 16.9, 10.2, 6.4 Hz, 1H), 5.05 (dq, J = 17.0, 1.6 Hz, 1H), 4.98 (ddt, J = 10.2, 2.2, 1.2 Hz, 1H), 2.90 (dd, J = 9.0, 4.3 Hz, 2H), 2.54-2.46 (m, 1H), 2.44-2.38 (m, 1H), 2.33-2.18 (m, 5H), 2.16-2.07 (m, 1H), 2.09-1.93 (m, 3H), 1.68-1.40 (m, 6H), 0.90 (s, 3H). ^{13}**C NMR** (100 MHz, $CDCl_3$) δ 220.89, 138.54, 138.14, 136.49, 135.41, 129.85, 129.55, 126.57, 125.50, 123.42, 114.88, 50.51, 48.01, 44.42, 38.22, 35.87, 33.62, 32.46, 31.61, 29.41, 26.54, 25.75, 21.60, 13.86. **HRMS** Calculated for $C_{24}H_{31}O$ $[M+H]^+$ 335.2369, found 335.2361.

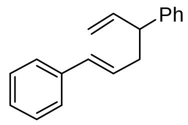

(*E*)-hexa-1,5-diene-1,4-diyldibenzene (3x): Prepared according to the general procedure, 90 °C, 18 h, 6% yield [with Cu(OAc) (10 mol%), 34% yield], known compound,[4] colorless oil, R_f = 0.5 (petroleum ether). ^{1}**H NMR** (400 MHz, $CDCl_3$) δ 7.36-7.15 (m, 10H), 6.38 (dt, J = 15.8, 1.4 Hz, 1H), 6.13 (dt, J = 15.8, 7.1 Hz, 1H), 6.03 (ddd, J = 16.6, 10.7, 7.4 Hz, 1H), 5.11-5.06 (m, 1H), 5.05 (dt, J = 8.4, 1.4 Hz, 1H), 3.43 (q, J = 7.4 Hz, 1H), 2.64 (tdd, J = 7.2, 2.9, 1.4 Hz, 2H). ^{13}**C NMR** (100 MHz, $CDCl_3$) δ 143.73, 141.48, 137.67, 131.41, 128.50, 128.45, 127.69, 126.96, 126.35, 126.04, 114.65, 50.02, 39.06.

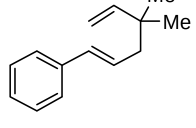

(*E*)-(4,4-dimethylhexa-1,5-dien-1-yl)benzene (3y): Prepared according to the general procedure, 90 °C, 18 h, 13% yield [with Cu(OAc) (10 mol%), 38% yield], known compound,[7] colorless oil, R_f = 0.7 (petroleum ether). ^{1}**H NMR** (400 MHz, $CDCl_3$) δ 7.36-7.32 (m, 2H), 7.31-7.26 (m, 2H), 7.21-7.17 (m, 1H), 6.36 (dt, J = 15.8, 1.3 Hz, 1H), 6.19 (dt, J = 15.7, 7.4 Hz, 1H), 5.86 (dd, J = 17.7, 10.4 Hz, 1H), 4.95 (dd, J = 10.4, 1.4 Hz, 1H), 4.94 (dd, J = 17.8, 1.4 Hz, 1H), 2.19 (dd, J = 7.5, 1.3 Hz, 2H). 1.55 (s, 6H). ^{13}**C NMR** (100 MHz, $CDCl_3$) δ 148.09, 137.83, 132.03, 128.46, 127.57, 126.87, 126.02, 110.58, 46.14, 37.22, 26.67.

5. Typical procedure for the synthesis of 3v and 3w

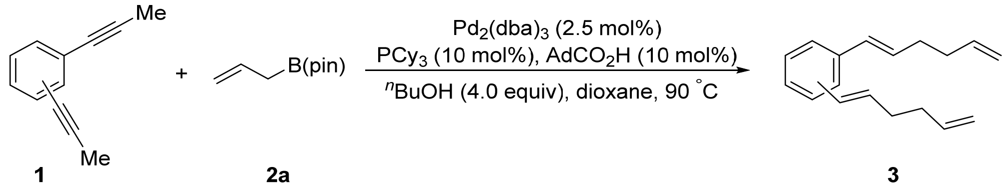

In glove box, a sealed tube was charged with alkynes **1** (0.2 mmol, 1.0 equiv), **2a** (0.8 mmol, 4.0 equiv), $Pd_2(dba)_3$ (0.005mmol, 2.5 mol%), PCy_3 (0.02 mmol, 10 mol%), $AdCO_2H$ (0.02 mmol, 10 mol%) and ^{n}BuOH (0.8 mmol, 4.0 equiv) in dioxane (1.0 mL). The reaction tube was sealed with a Teflon screw cap, removed from the glove box. Then, the reaction mixture was stirred at 90 °C for 8-10 h. After cooling to room temperature, the reaction mixture was directly purified by column chromatography on silica gel using *n*-pentene to afford the corresponding product **3**.

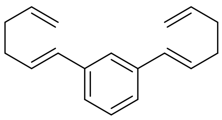

1,3-di((E)-hexa-1,5-dien-1-yl)benzene (3v): Prepared according to the general procedure, 10 h, 57% yield, colorless oil, R_f = 0.6 (petroleum ether). **^{1}H NMR** (400 MHz, $CDCl_3$) δ 7.29 (s, 1H), 7.24-7.16 (m, 3H), 6.39 (d, J = 15.9 Hz, 2H), 6.22 (dt, J = 15.9, 6.5 Hz, 2H), 5.92-5.82 (m, 2H), 5.09-4.98 (m, 4H), 2.34-2.28 (m, 4H), 2.26-2.20 (m, 4H); **^{13}C NMR** (100 MHz, $CDCl_3$) δ 138.11, 137.92, 130.18, 130.17, 128.61, 124.57, 123.75, 114.93, 33.56, 32.46. **HRMS** Calculated for $C_{18}H_{23}$ $[M+H]^+$ 239.1794, found 239.1789.

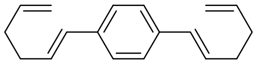

1,4-di((E)-hexa-1,5-dien-1-yl)benzene (3w): Prepared according to the general procedure, 8 h, 64% yield, colorless oil, R_f = 0.7 (petroleum ether). **^{1}H NMR** (400 MHz, $CDCl_3$) δ 7.26 (s, 4H), 6.37 (dt, J = 15.8, 1.4 Hz, 2H), 6.20 (dt, J = 15.8, 6.6, 2H), 5.86 (ddt, J = 16.7, 10.1, 6.4, 2H), 5.06 (dq, J = 17.2, 1.6 Hz, 2H), 4.99 (ddt, J = 10.2, 2.1, 1.2 Hz, 2H), 2.33-2.28 (m, 4H), 2.26-2.18 (m, 4H); **^{13}C NMR** (100 MHz, $CDCl_3$) δ 138.13, 136.44, 129.94, 129.68, 126.10, 114.91, 33.59, 32.49. **HRMS** Calculated for $C_{18}H_{23}$ $[M+H]^+$ 239.1794, found 239.1789.

6. Typical procedure for palladium-catalyzed allyl-alkenyl coupling reaction

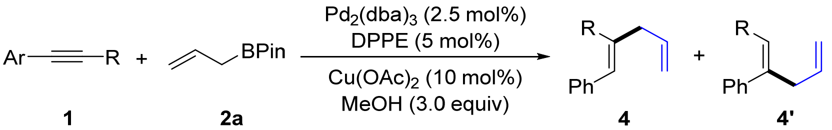

In glove box, a sealed tube was charged with alkynes **1** (0.2 mmol, 1.0 equiv), **2a** (0.4 mmol, 2.0 equiv), $Pd_2(dba)_3$ (0.005 mmol, 2.5 mol%), dppe (0.01 mmol, 5.0 mol%), $Cu(OAc)_2$ (0.02 mmol, 10 mol%) and MeOH (0.6 mmol, 3.0 equiv) in dioxane (0.5 mL). The reaction tube was sealed with a Teflon screw cap, removed from the glove box. Then, the reaction mixture was stirred at 70 °C for 24 h. After cooling to room temperature, the reaction mixture was directly purified by column chromatography on silica gel using *n*-pentene and ethyl acetate to afford the corresponding product **4**.

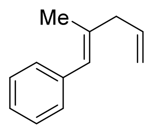

(*E*)-(2-methylpenta-1,4-dien-1-yl)benzene (4a): Prepared according to the general procedure, 71% total yield, the ratio of **4a**/**4a'** is 11.9:1, known compound,[8] colorless oil, R_f = 0.8 (petroleum ether). **^{1}H NMR** (400 MHz, $CDCl_3$) *δ* 7.33-7.29 (m, 2H), 7.25-7.23 (m, 2H), 7.20-7.16 (m, 1H), 6.30 (s, 1H), 5.89 (ddt, *J* = 17.0, 10.0, 6.8 Hz, 1H), 5.15-5.07 (m, 2H), 2.90 (dd, *J* = 6.8, 1.4 Hz, 2H), 1.85 (d, *J* = 1.4 Hz, 3H); **^{13}C NMR** (100 MHz, $CDCl_3$) *δ* 138.45, 137.29, 136.42, 128.82, 128.04, 125.98, 125.76, 116.35, 44.99, 17.86.

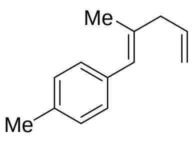

(*E*)-1-methyl-4-(2-methylpenta-1,4-dien-1-yl)benzene (4b): Prepared according to the general procedure, 62% total yield, the ratio of **4b**/**4b'** is 7.9:1, colorless oil, R_f = 0.8 (petroleum ether). **^{1}H NMR** (400 MHz, $CDCl_3$) *δ* 7.15-7.10 (m, 4H); 6.26 (s, 1H); 5.88 (ddt, *J* = 17.0, 10.0, 6.8 Hz, 1H), 5.14-5.06 (m, 2H), 2.89 (dd, *J* = 6.8, 1.5 Hz, 2H), 2.32 (s, 3H) 1.84 (d, *J* = 1.5 Hz, 3H); **^{13}C NMR** (100 MHz, $CDCl_3$) *δ* 136.55, 136.53, 135.56, 135.55, 128.75, 128.72, 125.62, 116.24, 45.04, 21.15, 17.86. **HRMS** Calculated for $C_{13}H_{16}$ 172.1252, found 172.1260.

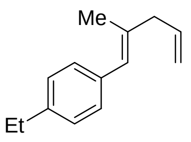

(*E*)-1-ethyl-4-(2-methylpenta-1,4-dien-1-yl)benzene (4c): Prepared according to the general procedure, 70% total yield, the ratio of **4c**/**4c'** is 8.7:1, colorless oil, R_f = 0.8 (petroleum ether). **^{1}H NMR** (400 MHz, $CDCl_3$) *δ* 7.18-7.13 (m, 4H), 6.27 (s, 1H), 5.88 (ddt, *J* = 17.0, 10.0, 6.8 Hz, 1H), 5.14-5.06 (m, 2H), 2.89 (dd, *J* = 6.9, 1.4 Hz, 2H), 2.63 (q, *J* = 7.6 Hz, 2H), 1.85 (d, *J* = 1.4 Hz, 3H), 1.23 (t, *J* = 7.6 Hz, 3H); **^{13}C NMR** (100 MHz, $CDCl_3$) *δ* 141.96, 136.56, 136.53, 135.80, 128.78, 127.53, 125.64, 116.22, 45.06, 28.56, 17.87, 15.58. **HRMS** Calculated for $C_{14}H_{18}$ 186.1409, found 186.1412.

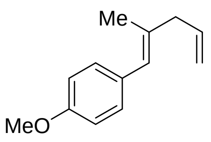

(*E*)-1-methoxy-4-(2-methylpenta-1,4-dien-1-yl)benzene (4d): Prepared according to the general procedure, 70% total yield, the ratio of **4d**/**4d'** is 5.6:1, colorless oil, R_f = 0.6 (petroleum ether). **^{1}H NMR** (400 MHz, $CDCl_3$) *δ* 7.20-7.16 (m, 2H), 6.87-6.84 (m, 2H), 6.23 (s, 1H), 5.88 (ddt, *J* = 16.9, 10.0, 6.9 Hz, 1H), 5.14-5.06 (m, 2H), 3.80 (s, 3H), 2.88 (dd, *J* = 6.9, 1.3 Hz, 2H), 1.84 (d, *J* = 1.4 Hz, 3H); **^{13}C NMR** (100 MHz, $CDCl_3$) *δ* 157.80, 136.60, 132.80, 129.91 125.17, 116.18, 113.83, 113.48, 55.24, 45.03, 17.80. **HRMS** Calculated for $C_{13}H_{17}O$ $[M+H]^+$ 189.1274, found 189.1266.

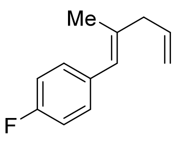

(*E*)-1-fluoro-4-(2-methylpenta-1,4-dien-1-yl)benzene (4e): Prepared according to the general procedure, 71% total yield, the ratio of **4e**/**4e'** is 12.0:1, colorless oil, R_f = 0.8 (petroleum ether). **^{1}H NMR** (400 MHz, $CDCl_3$) *δ* 7.19 (dd, *J* = 8.4, 5.5 Hz, 2H), 6.99 (t, *J* = 8.5 Hz, 2H), 6.25 (s, 1H), 5.88 (ddt, *J* = 17.0, 10.2, 6.9 Hz, 1H), 5.17-5.05 (m, 2H), 2.89 (d, *J* = 6.8 Hz, 2H), 1.82 (s, 3H); **^{13}C NMR** (100 MHz, $CDCl_3$) *δ* ^{13}C NMR (100 MHz, $CDCl_3$) *δ* 161.14 (d, *J* = 243.69 Hz), 137.23, 136.26, 134.40 (d, *J* = 3.3 Hz), 130.26 (d, *J* = 7.6 Hz), 124.63, 116.42, 114.85 (d, *J* = 21.1 Hz), 44.84, 17.74. **^{19}F NMR** (376 MHz, $CDCl_3$) *δ* -116.62. **HRMS** Calculated for $C_{12}H_{13}F$ 176.1001, found 176.1006.

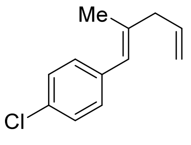

(*E*)-1-chloro-4-(2-methylpenta-1,4-dien-1-yl)benzene (4f): Prepared according to the general procedure, 75% total yield, the ratio of **4f**/**4f'** is 18.5:1, colorless oil, R_f = 0.8 (petroleum ether). **^{1}H NMR** (400 MHz, $CDCl_3$) *δ* 7.27 (d,

J = 8.2 Hz, 2H), 7.16 (d, *J* = 8.2 Hz, 2H), 6.23 (s, 1H), 5.87 (ddt, *J* = 16.9, 10.0, 6.8 Hz, 1H), 5.15 – 5.07 (m, 2H), 2.89 (d, *J* = 6.8 Hz, 2H), 1.83 (s, 3H); **^{13}C NMR** (100 MHz, $CDCl_3$) δ ^{13}C NMR (100 MHz, $CDCl_3$) δ 138.13, 136.85, 136.11, 131.63, 130.08, 128.16, 124.59, 116.56, 44.90, 17.85. **HRMS** Calculated for $C_{12}H_{13}Cl$ 192.0706, found 192.0710.

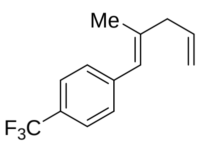

(*E*)-1-trifluoromethyl-4-(2-methylpenta-1,4-dien-1-yl)benzene (4g): Prepared according to the general procedure, 86% total yield, the ratio of **4g**/**4g'** is > 20.0:1, colorless oil, R_f = 0.4 (petroleum ether). **^{1}H NMR** (400 MHz, $CDCl_3$) δ 7.56 (d, *J* = 8.0 Hz, 2H), 7.32 (d, *J* = 8.0 Hz, 2H), 6.31 (s, 1H), 5.88 (ddt, *J* = 17.0, 10.2, 6.9 Hz, 1H), 5.17-5.09 (m, 2H), 2.92 (d, *J* = 6.9 Hz, 2H), 1.85 (s, 3H).; **^{13}C NMR** (100 MHz, $CDCl_3$) δ ^{13}C NMR (100 MHz, $CDCl_3$) δ 142.05, 139.76, 135.85, 128.96, 127.95 (q, *J* = 32.2 Hz), 124.95 (q, *J* = 3.8 Hz), 124.59, 124.35 (q, *J* = 270.1 Hz), 116.77, 44.89, 17.93. **^{19}F NMR** (376 MHz, $CDCl_3$) δ -62.37. **HRMS** Calculated for $C_{12}H_{13}F$ 226.0969, found 226.0979.

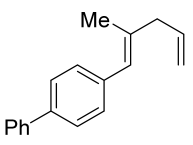

(*E*)-4-(2-methylpenta-1,4-dien-1-yl)-1,1'-biphenyl (4h): Prepared according to the general procedure, 88% total yield, the ratio of **4h**/**4h'** is 17.9:1, colorless oil, R_f = 0.7 (petroleum ether). **^{1}H NMR** (400 MHz, $CDCl_3$) δ 7.65-7.58 (m, 2H), 7.56-7.54 (m, 2H), 7.44-7.40 (m, 2H), 7.33-7.31 (m, 3H), 6.32 (s, 1H), 5.90 (ddt, *J* = 16.9, 10.0, 6.8 Hz, 1H), 5.16-5.08 (m, 2H), 2.92 (dd, *J* = 6.8, 1.4 Hz, 2H), 1.90 (d, *J* = 1.4 Hz, 3H); **^{13}C NMR** (100 MHz, $CDCl_3$) δ 140.97, 138.74, 137.64, 137.53, 136.41, 129.28, 128.79, 127.16, 126.99, 126.78, 125.42, 116.46, 45.16, 18.07. **HRMS** Calculated for $C_{18}H_{18}$ 234.1409, found 234.1412.

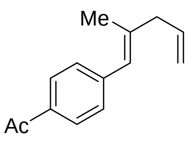

(*E*)-1-acetyl-(4-(2-methylpenta-1,4-dien-1-yl)benzene (4i): Prepared according to the general procedure, 78% total yield, the ratio of **4i**/**4i'** is > 20.0:1, colorless oil, R_f = 0.6 (petroleum ether/EtOAc 20/1). **^{1}H NMR** (400 MHz, $CDCl_3$) δ 7.91 (d, *J* = 8.4 Hz, 2H), 7.33 (d, *J* = 8.3 Hz, 2H), 6.32 (s, 1H), 5.88 (ddt, *J* = 17.0, 10.1, 6.9 Hz, 1H), 5.17-5.10 (m, 2H), 2.93 (dd, *J* = 6.7, 1.5 Hz, 2H), 2.59 (s, 3H), 1.89 (d, *J* = 1.3 Hz, 3H); **^{13}C NMR** (100 MHz, $CDCl_3$) δ 197.69, 143.45, 140.11, 135.85, 134.69, 128.87, 128.23, 125.00, 116.81, 45.07, 26.55, 18.16. **HRMS** Calculated for $C_{14}H_{17}O$ $[M+H]^+$ 201.1274, found 201.1270.

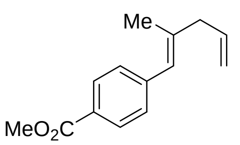

Methyl (*E*)-4-(2-methylpenta-1,4-dien-1-yl)benzoate (4j): Prepared according to the general procedure, 99% total yield, the ratio of **4j**/**4j'** is > 20.0:1, colorless oil, R_f = 0.5 (petroleum ether/EtOAc 10/1). **^{1}H NMR** (400 MHz, $CDCl_3$) δ 7.98 (d, *J* = 8.4 Hz, 2H), 7.30 (d, *J* = 8.3 Hz, 2H), 6.32 (s, 1H), 5.88 (ddt, *J* = 17.0, 10.1, 6.9 Hz, 1H), 5.20-5.07 (m, 2H), 3.90 (s, 3H), 2.92 (dd, *J* = 6.9, 1.4 Hz, 2H), 1.87 (d, *J* = 1.3 Hz, 3H); **^{13}C NMR** (100 MHz, $CDCl_3$) δ 167.04, 143.21, 139.81, 135.90, 129.39, 128.69, 127.54, 125.07, 116.75, 51.99, 45.04, 18.10 **HRMS** Calculated for $C_{14}H_{17}O_2$ $[M+H]^+$ 217.1223, found 217.1226.

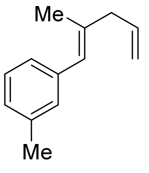

(*E*)-1-methyl-3-(2-methylpenta-1,4-dien-1-yl)benzene (4k): Prepared according to the general procedure, 87% total yield, the ratio of **4k**/**4k'** is 9.6:1, colorless oil,

R_f = 0.7 (petroleum ether). **^{1}H NMR** (400 MHz, $CDCl_3$) *δ* 7.23-7.18 (m, 1H), 7.06-7.00 (m, 3H), 6.27 (s, 1H), 5.88 (ddt, *J* = 16.9, 10.0, 6.8 Hz, 1H), 5.14-5.06 (m, 2H), 2.89 (dd, *J* = 6.8, 1.4 Hz, 2H), 2.34 (s, 3H), 1.85 (d, *J* = 1.4 Hz, 3H) ; **^{13}C NMR** (100 MHz, $CDCl_3$) *δ* 138.40, 137.53, 137.09, 136.49, 129.59, 127.94, 126.76, 125.87, 125.83, 116.30, 45.01, 21.49, 17.89. **HRMS** Calculated for $C_{13}H_{16}$ 172.1252, found 172.1259.

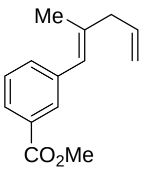

Methyl (*E*)-3-(2-methylpenta-1,4-dien-1-yl)benzoate (4l): Prepared according to the general procedure, 93% total yield, the ratio of **4l**/**4l'** is 20.0:1, colorless oil, R_f = 0.6 (petroleum ether/EtOAc 10/1). **^{1}H NMR** (400 MHz, $CDCl_3$) *δ* 7.92 (t, *J* = 1.8 Hz, 1H), 7.86 (dt, *J* = 7.4, 1.6 Hz, 1H), 7.46-7.34 (m, 2H), 6.31 (s, 1H), 5.88 (ddt, *J* = 17.0, 10.1, 6.9 Hz, 1H), 5.38-5.04 (m, 2H), 3.91 (s, 3H), 2.91 (dd, *J* = 6.9, 1.4 Hz, 2H), 1.85 (d, *J* = 1.4 Hz, 3H); **^{13}C NMR** (100 MHz, $CDCl_3$) *δ* 167.21, 138.69, 138.62, 136.09, 133.23, 129.96, 129.92, 128.09, 127.12, 124.81, 116.60, 52.09, 44.85, 17.85. **HRMS** Calculated for $C_{14}H_{17}O_2$ $[M+H]^+$ 217.1223, found 217.1224.

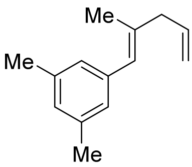

(*E*)-1,3-dimethyl-5-(2-methylpenta-1,4-dien-1-yl)benzene (4m): Prepared according to the general procedure, 65% total yield, the ratio of **4m**/**4m'** is 11.8:1, colorless oil, R_f = 0.7 (petroleum ether). **^{1}H NMR** (400 MHz, $CDCl_3$) *δ* 6.87 (s, 2H), 6.83 (s, 1H), 6.24 (s, 1H), 5.88 (ddt, *J* = 16.9, 10.0, 6.8 Hz, 1H), 5.18-5.04 (m, 2H), 2.88 (dd, *J* = 6.8, 1.4 Hz, 2H), 2.30 (s, 6H), 1.85 (d, *J* = 1.4 Hz, 3Hz); **^{13}C NMR** (100 MHz, $CDCl_3$) *δ* 138.36, 137.44, 136.89, 136.54, 127.68, 126.64, 125.86, 116.23, 45.01, 21.36, 17.92. **HRMS** Calculated for $C_{14}H_{18}$ 186.1409, found 186.1410.

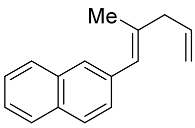

(*E*)-2-(2-methylpenta-1,4-dien-1-yl)naphthalene (4o): Prepared according to the general procedure, 80% total yield, the ratio of **4n**/**4n'** is 18.4:1, colorless oil, R_f = 0.6 (petroleum ether). **^{1}H NMR** (400 MHz, $CDCl_3$) *δ* 7.80-7.76 (m, 3H), 7.68 (s, 1H), 7.46-7.37 (m, 3H), 6.44 (s, 1H), 5.93 (ddt, *J* = 17.0, 10.0, 6.9 Hz, 1H), 5.18-5.10 (m, 2H), 2.95 (dd, *J* = 6.8, 1.4 Hz, 2H), 1.93 (d, *J* = 1.3 Hz, 1H); **^{13}C NMR** (100 MHz, $CDCl_3$) *δ* 137.90, 136.40, 136.00, 133.41, 131.93, 127.83, 127.58, 127.56, 127.47, 127.27, 125.96, 125.80, 125.46, 116.48, 45.08, 18.05. **HRMS** Calculated for $C_{16}H_{16}$ 208.1252, found 208.1260.

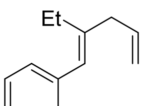

(*E*)-(2-ethylpenta-1,4-dien-1-yl)benzene (4p): Prepared according to the general procedure, 49% total yield, the ratio of **4o**/**4o'** is 13.3:1, colorless oil, R_f = 0.8 (petroleum ether). **^{1}H NMR** (400 MHz, $CDCl_3$) *δ* 7.32-7.29 (m, 2H), 7.24-7.19 (m, 3H), 6.26 (s, 1H), 5.89 (ddt, *J* = 16.9, 10.0, 6.9 Hz, 1H), 5.16-5.07 (m, 2H), 2.92 (dd, *J* = 6.8, 1.3 Hz, 2 H), 2.26 (qd, *J* = 7.5, 0.7 Hz, 2H), 1.08 (t , *J* = 7.6 Hz, 3H); **^{13}C NMR** (100 MHz, $CDCl_3$) *δ* 143.12, 138.38, 136.68, 128.56, 128.09, 126.03, 125.51, 116.29, 41.27, 23.87, 12.93. **HRMS** Calculated for $C_{13}H_{16}$ 172.1252, found 172.1256.

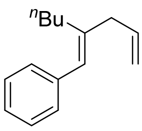

(*E*)-(2-allylhex-1-en-1-yl)benzene (4q): Prepared according to the general procedure, 60% total yield, the ratio of **4p**/**4p'** is 12.8:1, colorless oil, R_f = 0.8 (petroleum ether). **^{1}H NMR** (400 MHz, $CDCl_3$) *δ* 7.32-7.28 (m, 2H); 7.22-7.14 (m, 3H), 6.28 (s, 1H); 5.88 (ddt, *J* = 16.9, 10.0, 6.9 Hz, 1H), 5.20-5.03 (m, 2H), 2.91

(dd, J = 6.9, 1.4 Hz, 2H), 2.23 (t, J = 8.0 Hz, 2H), 1.55-1.41 (m, 2H), 1.31 (h, J = 7.2 Hz, 2H), 0.88 (t, J = 7.3 Hz, 3H); **^{13}C NMR** (100 MHz, $CDCl_3$) δ 141.93, 138.46, 136.74, 128.61, 128.06, 125.98, 125.91, 116.25, 41.77, 30.63, 30.40, 22.85, 13.97. **HRMS** Calculated for $C_{15}H_{20}$ 200.1565, found 200.1569.

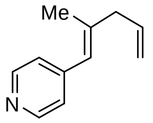

(*E*)-4-(2-methylpenta-1,4-dien-1-yl)pyridine (4r): Prepared according to the general procedure, 23% yield, the ratio of **4r**/**4r** is > 20.0:1 yellow oil, R_f = 0.3 (petroleum ether/EtOAc 5/1). **^{1}H NMR** (400 MHz, $CDCl_3$) δ 8.53 (d, J = 5.1 Hz, 2H), 7.14-7.13 (m, 2H), 6.22 (s, 1H), 5.87 (ddt, J = 17.0, 10.2, 6.9 Hz, 1H), 5.17 – 5.14 (m, 1H), 5.12 (t, J = 1.3 Hz, 1H), 2.93 (dd, J = 6.9, 1.4 Hz, 2H), 1.90 (d, J = 1.4 Hz, 3H). **^{13}C NMR** (100 MHz, $CDCl_3$) δ 149.50, 146.01, 142.12, 135.47, 123.58, 123.51, 117.09, 45.00, 18.18. **HRMS** Calculated for $C_{11}H_{14}N$ $[M+H]^+$ 160.1121, found 160.1142.

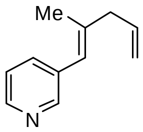

(*E*)-3-(2-methylpenta-1,4-dien-1-yl)pyridine (4s): Prepared according to the general procedure, 57% total yield, the ratio of **4s**/**4s'** is > 20.0:1, known compound,[6] colorless oil, R_f = 0.3 (petroleum ether/EtOAc 5/1). **^{1}H NMR** (400 MHz, $CDCl_3$) δ 8.50 (d, J = 2.2 Hz, 1H), 8.42 (dd, J = 4.8, 1.6 Hz, 1H), 7.54 (dt, J = 7.9, 2.0 Hz, 1H), 7.24 (ddd, J = 7.9, 4.9, 0.9 Hz, 1H), 6.24 (s, 1H), 5.88 (ddt, J = 17.0, 10.1, 6.9 Hz, 1H), 5.17-5.12 (m, 1H), 5.12-5.10 (m, 1H), 2.93 (dd, J = 6.8, 1.4 Hz, 2H), 1.86 (d, J = 1.3 Hz, 3H). **^{13}C NMR** (100 MHz, $CDCl_3$) δ 150.08, 147.04, 140.06, 135.80, 135.70, 134.02, 122.94, 122.09, 116.82, 44.84, 17.90. **HRMS** Calculated for $C_{11}H_{14}N$ $[M+H]^+$ 160.1121, found 160.1141.

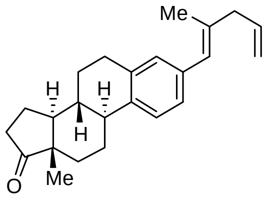

(8R,9S,13S,14S)-13-methyl-3-((*E*)-2-methylpenta-1,4-dien-1-yl)-6,7,8,9,11,12,13,14,15,16-decahydro-17H-cyclopenta[a]phenanthren-17-one (4t): Prepared according to the general procedure, 25% total yield, the ratio of **4t**/**4t'** is 13.6:1, colorless solid, m.p. 44-46 °C, R_f = 0.3 (petroleum ether/EtOAc 20/1). **^{1}H NMR** (400 MHz, $CDCl_3$) δ 7.24 (d, J = 7.9 Hz, 1H), 7.06 (dd, J = 8.2, 1.9 Hz, 1H), 6.99 (d, J = 1.9 Hz, 1H), 6.24 (s, 1H), 5.88 (ddt, J = 16.8, 10.0, 6.7 Hz, 1H), 5.14-5.06 (m, 2H), 2.93-2.87 (m, 4H), 2.55-2.46 (m, 1H), 2.46-2.40 (m, 1H), 2.33-2.27(m, 1H), 2.19-2.11 (m, 1H), 2.10-1.94 (m, 3H), 1.86 (d, J = 1.3 Hz, 3H), 1.68-1.39 (m, 6H); 0.91 (s, 3H); **^{13}C NMR** (100 MHz, $CDCl_3$) δ 220.91, 137.49, 136.82, 136.50, 136.04, 135.99, 129.39, 126.27, 125.41, 125.02, 116.24, 50.54, 48.02, 45.03, 44.40, 38.22, 35.88, 31.63, 29.45, 26.59, 25.72, 21.61, 17.92, 13.87. **HRMS** Calculated for $C_{24}H_{31}O$ $[M+H]^+$ 335.2369, found 335.2375.

7. Control and deuterium labeling experiments

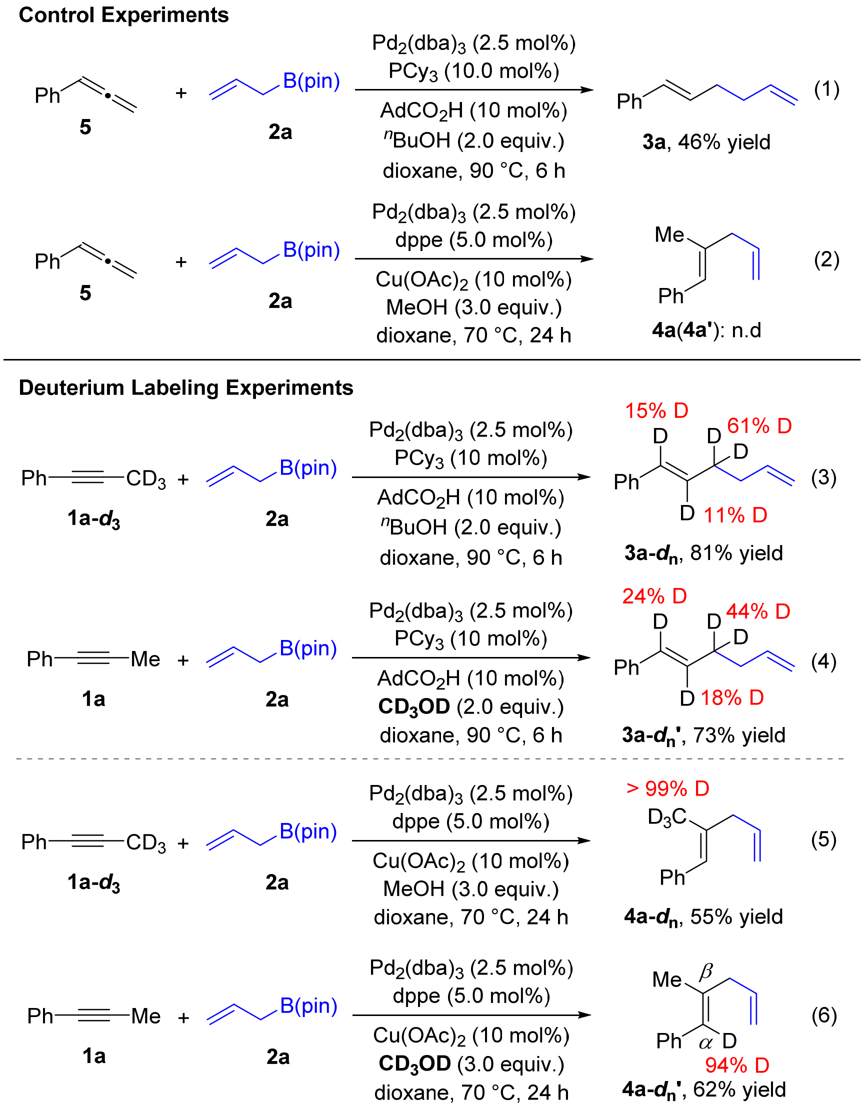

Scheme S1: Control and deuterium labeling experiments

To better interpret the results of deuterium-labeling studies, we provided more details about potential pathways for the Pd-acid catalyzed reaction using deuterated alkyne. As we illustrated in Scheme S2, if the hydrometallation of the allene's internal π-system is irreversible (Pathway A), the reaction should give 100% deuteration at the γ-position of 1,5-diene. This is contrary to the observations in the deuterium labeling experiments. Instead, when hydrometallation of the allene's internal π-system is reversible (Pathway B), deuterium label could be scrambled into the α-, β-, and γ-positions of 1,5-diene. The above deuterium-labeling studies indeed support the conclusion that hydrometallation of the allene's internal π-system is reversible under Pd-acid conditions. It should be noted that Pd-hydride could be generated from whether oxidative addition of Pd(0) with X-H or β-hydride elimination from deuterated vinyl-Pd species. Therefore, the Pd-hydride species were partially labelled.

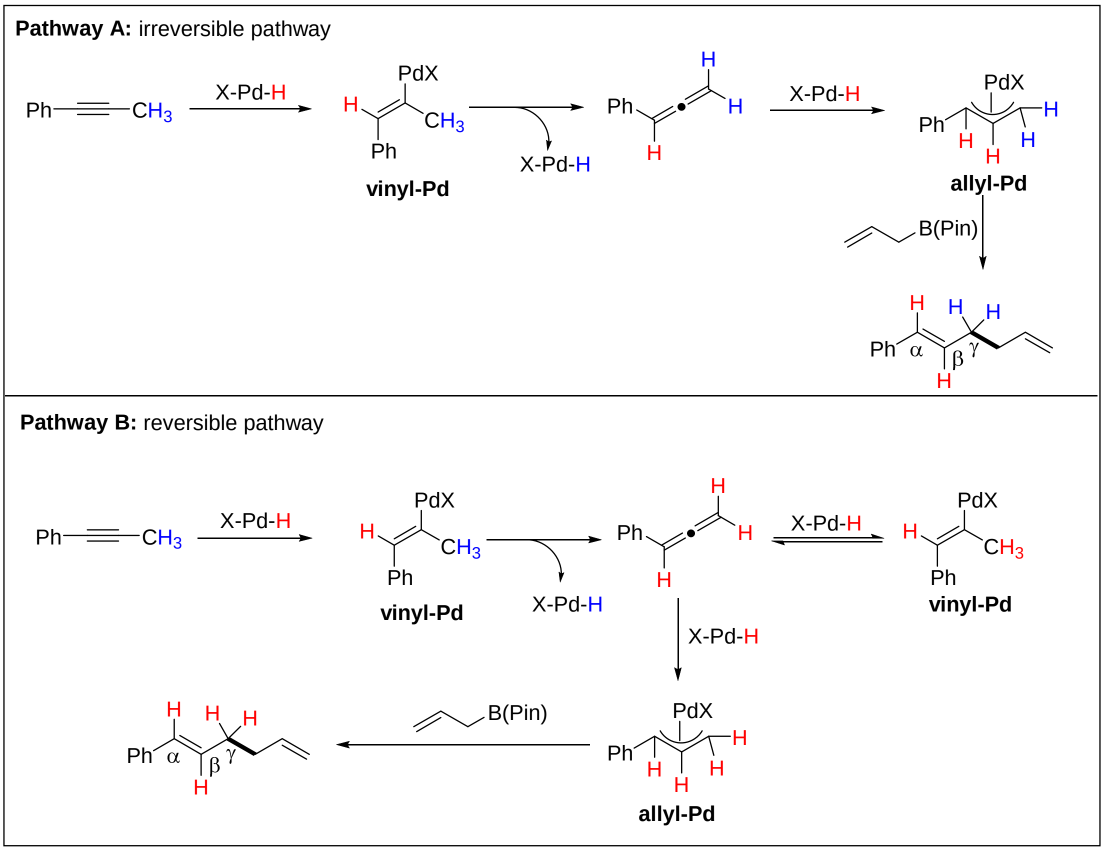

H: non-labelled hydrogen. H: totally labelled hydrogen. H: partially labelled hydrogen.

X-Pd-H: partially labelled Pd-hydride, generated from whether the oxidative addition of Pd(0) with X-H or β-hydride elimination from vinyl-Pd species

Scheme S2

8. References

1. Kippo, T.; Fukuyama, T.; Ryu, I. *Org. Lett.* **2011**, *13*, 3864.
2. Gao, S.; Liu, H.; Wu, Z.; Yao, H.; Lin, A. *Green Chem.* **2017**, *19*, 1861
3. Jiménez-Aquino, A.; Flegeau, E. F.; Schneider, U.; Kobayashi, S. *Chem. Commun.* **2011**, *47*, 9456.
4. Ding, F.; William, R.; Wang, F.; Liu, X.-W. *Chem. Commun.* **2012**, *48*, 8709.
5. Hamilton, J. Y.; Hauser, N.; Sarlah, D.; Carreira, E. M. *Angew. Chem. Int. Ed.* **2014**, *53*, 10759.
6. Flegeau, E. F.; Schneider, U.; Kobayashi, S. *Chem. Eur. J.* **2009**, *15*, 12247.
7. Araki, S.; Jin, S.-J.; Butsugan, Y. *J. Chem. Soc., Perkin Trans. 1*, **1995**, *0*, 549.
8. Matsubara, R.; Jamison, T. F. *J. Am. Chem. Soc.* **2010**, *132*, 6880.

9. Copy of NMR for products

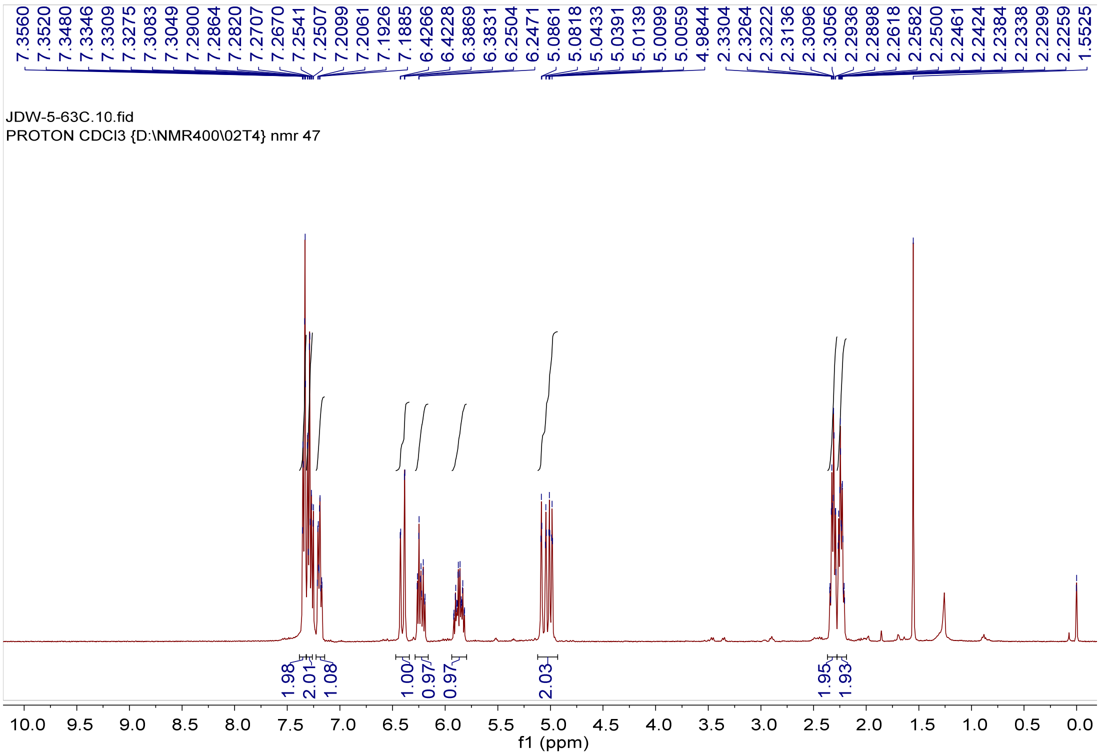

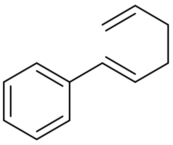

3a ^{1}H NMR (400 MHz, $CDCl_3$)
^{13}C NMR (100 MHz, $CDCl_3$)

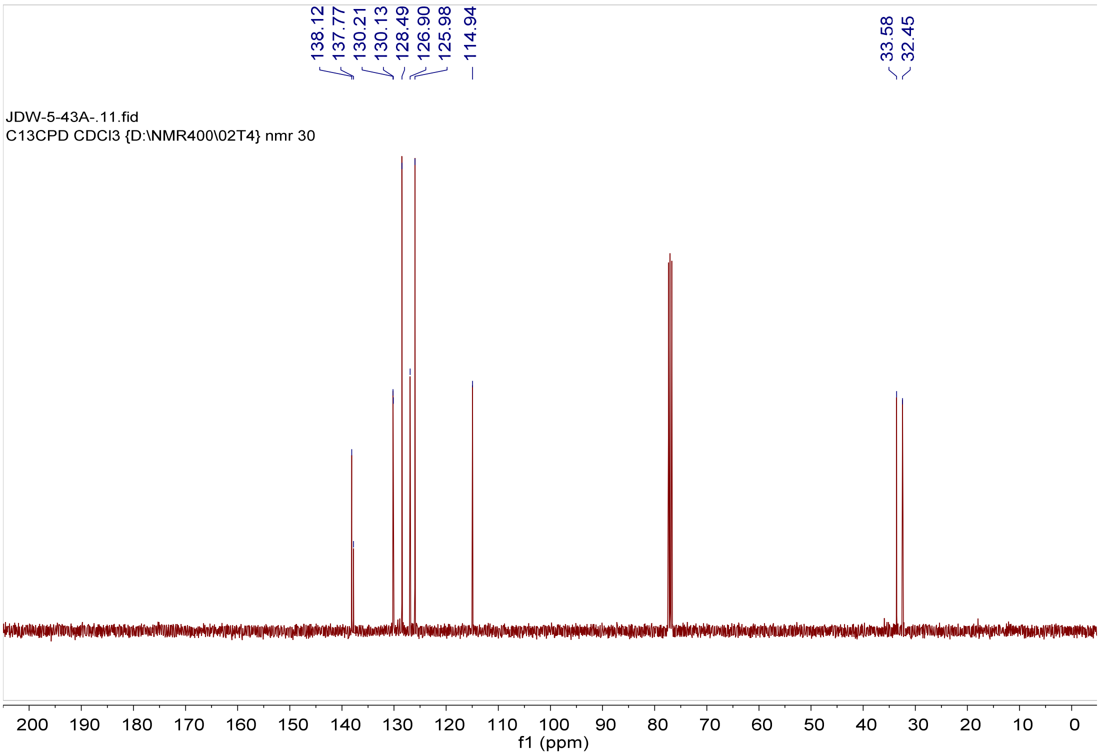

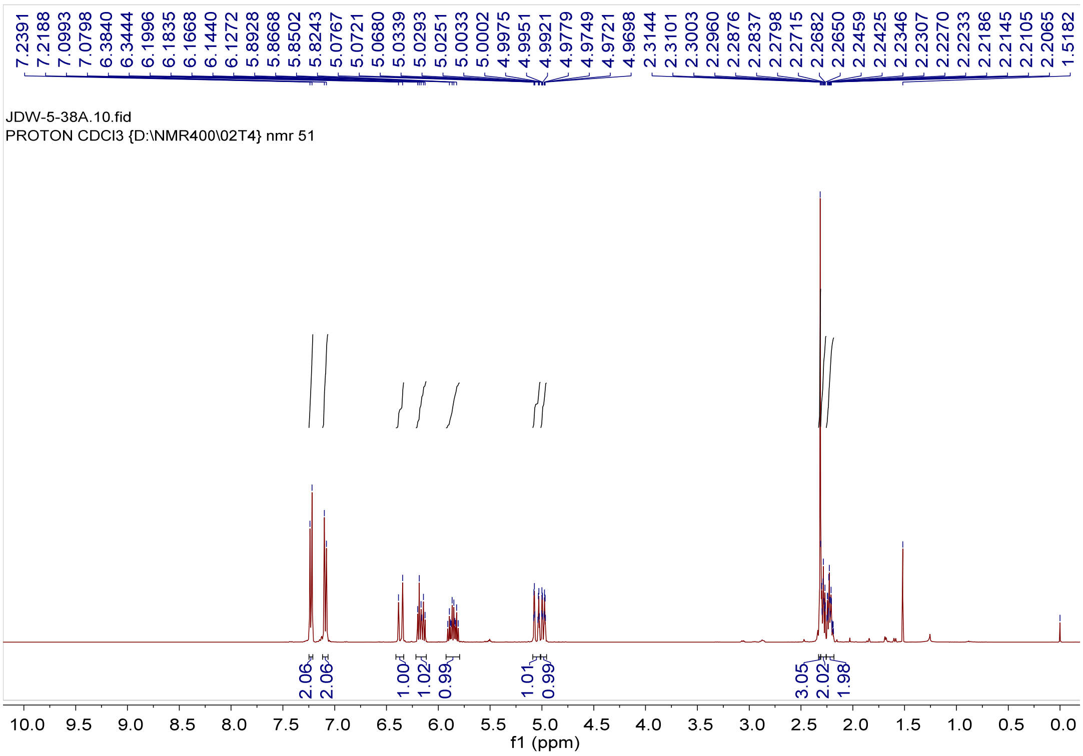

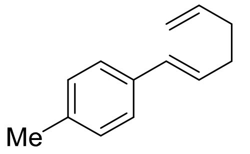

3b ^{1}H NMR (400 MHz, $CDCl_3$)

^{13}C NMR (100 MHz, $CDCl_3$)

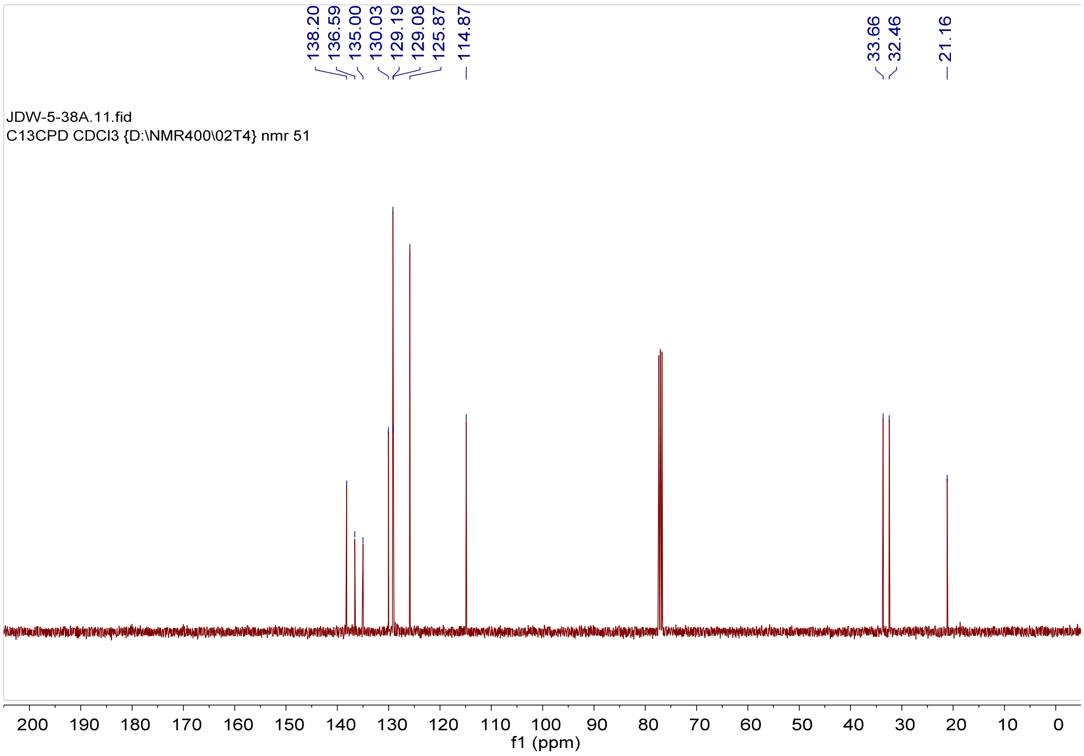

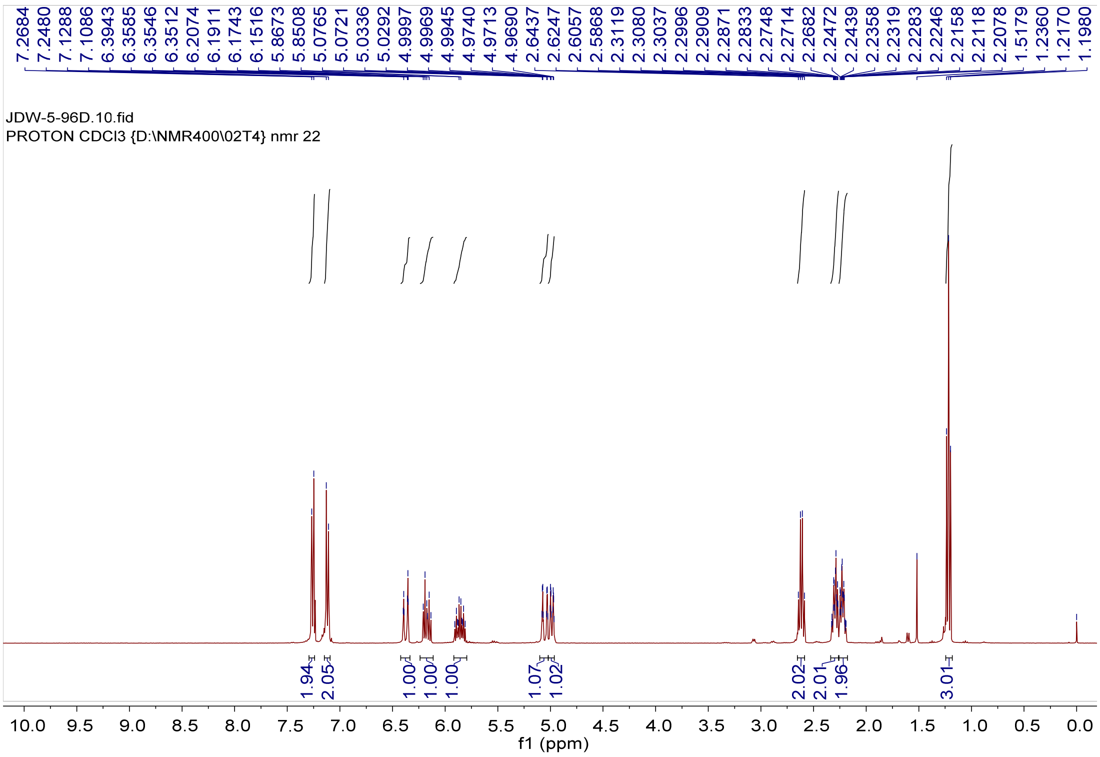

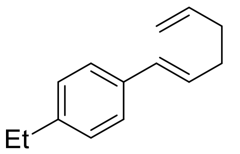

3c ^{1}H NMR (400 MHz, $CDCl_3$)
^{13}C NMR (100 MHz, $CDCl_3$)

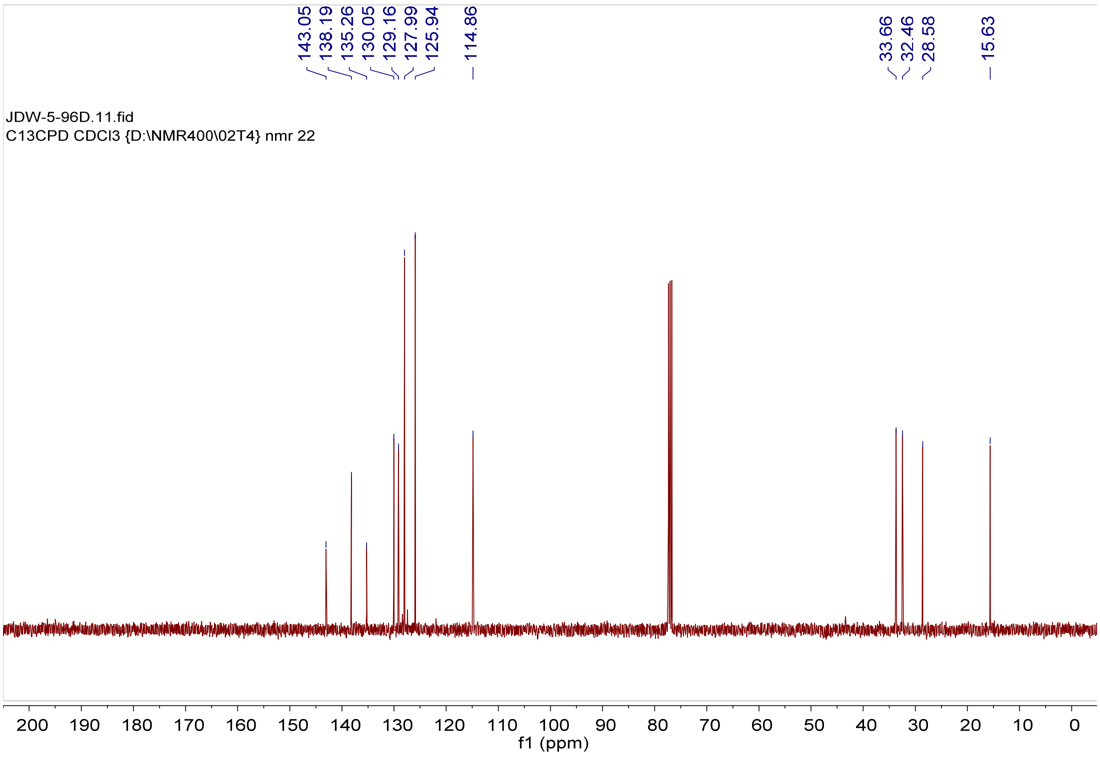

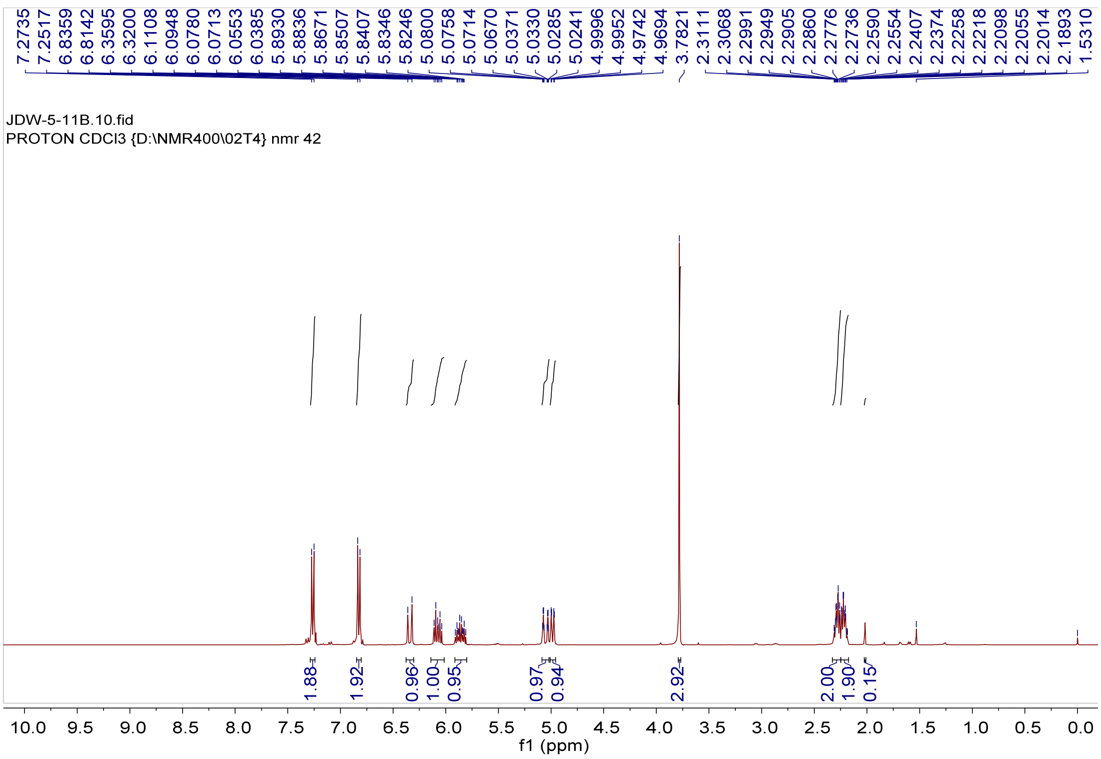

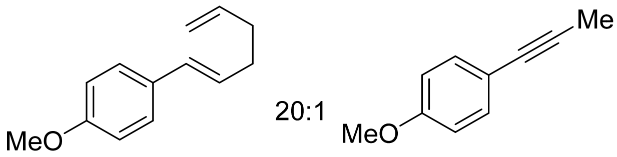

3d ^{1}H NMR (400 MHz, $CDCl_3$)
^{13}C NMR (100 MHz, $CDCl_3$)

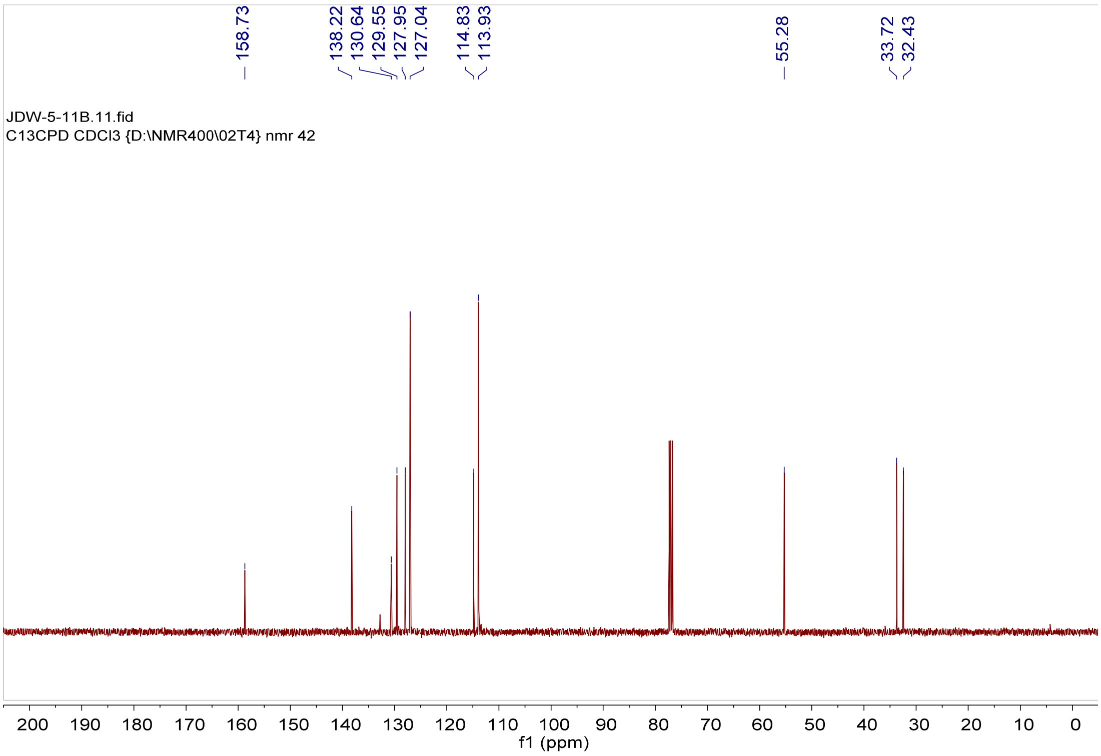

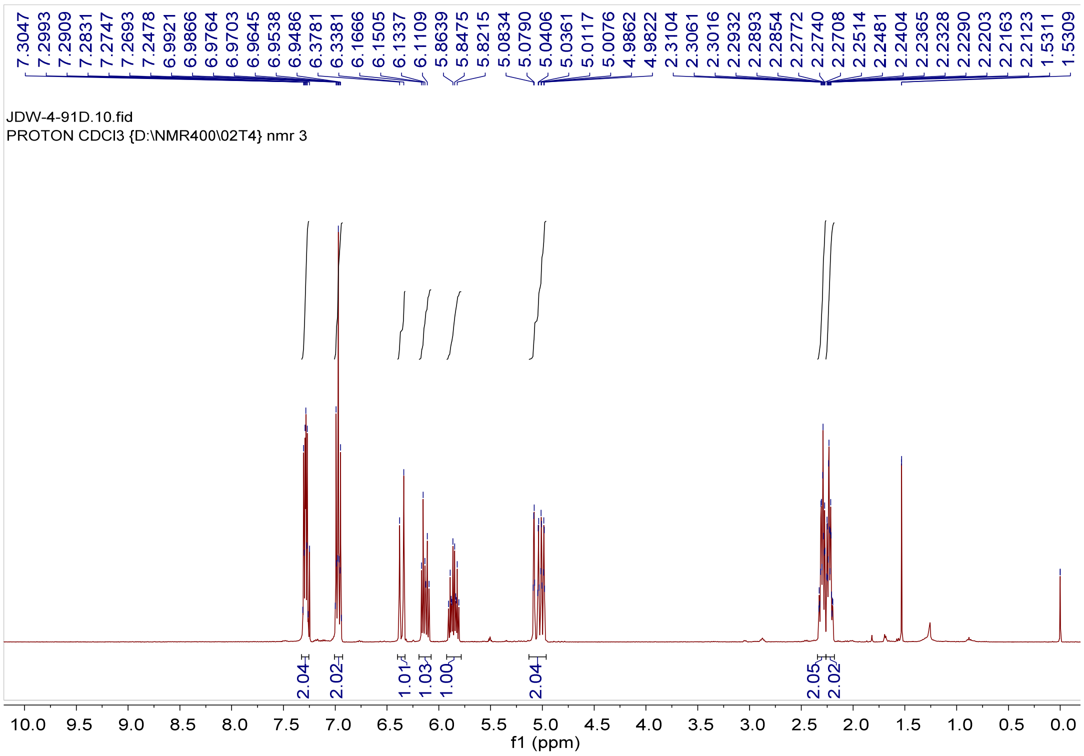

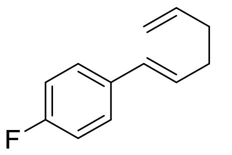

3e ^{1}H NMR (400 MHz, $CDCl_3$)

^{13}C NMR (100 MHz, $CDCl_3$)

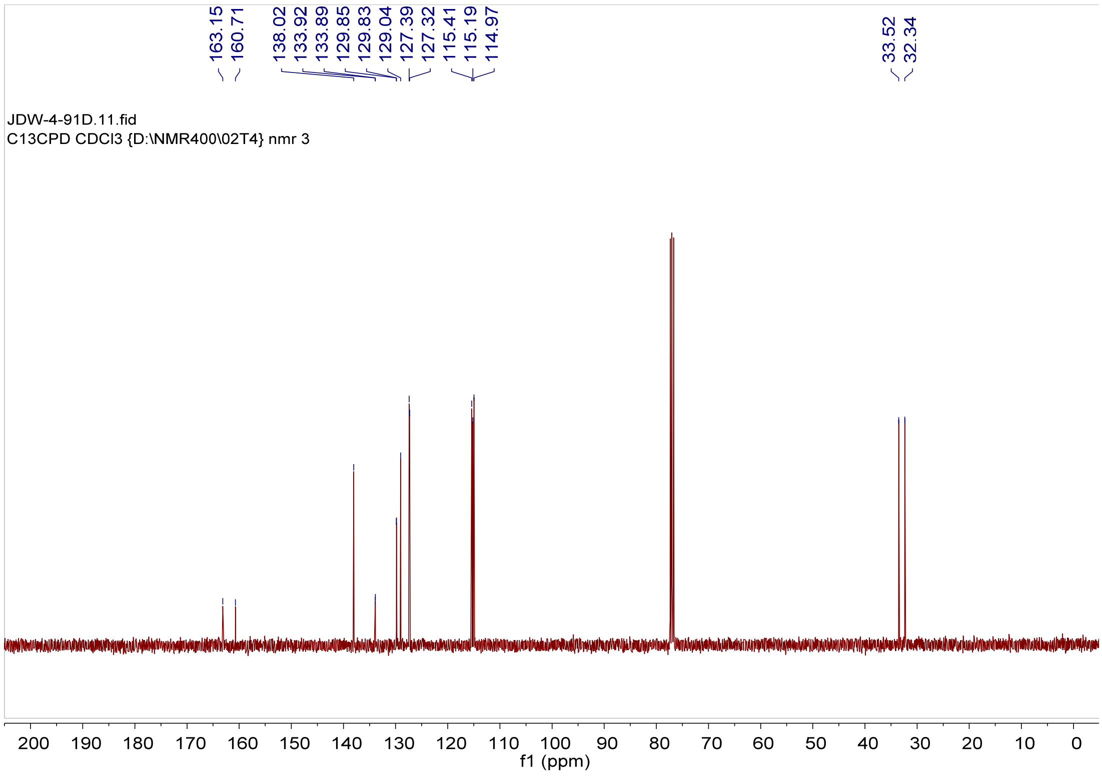

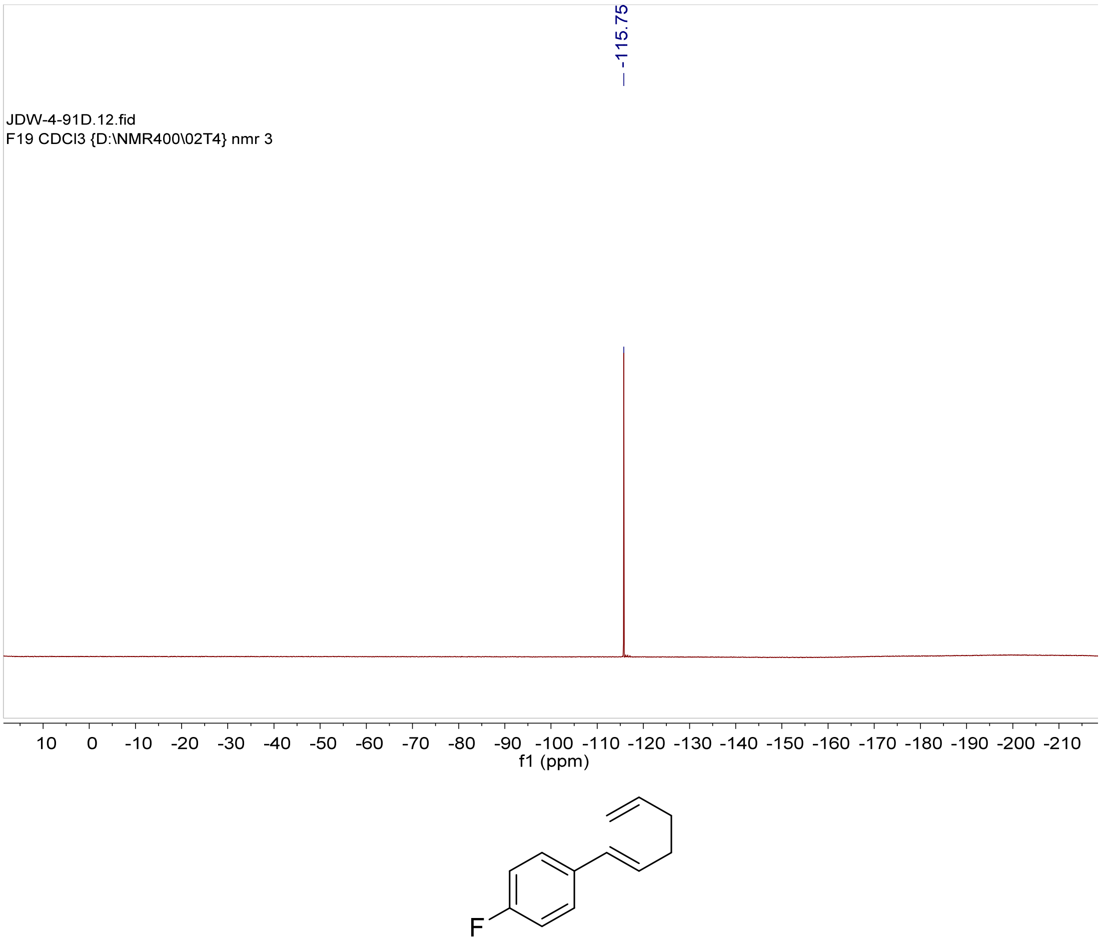

3e ^{19}F NMR (376 MHz, $CDCl_3$)

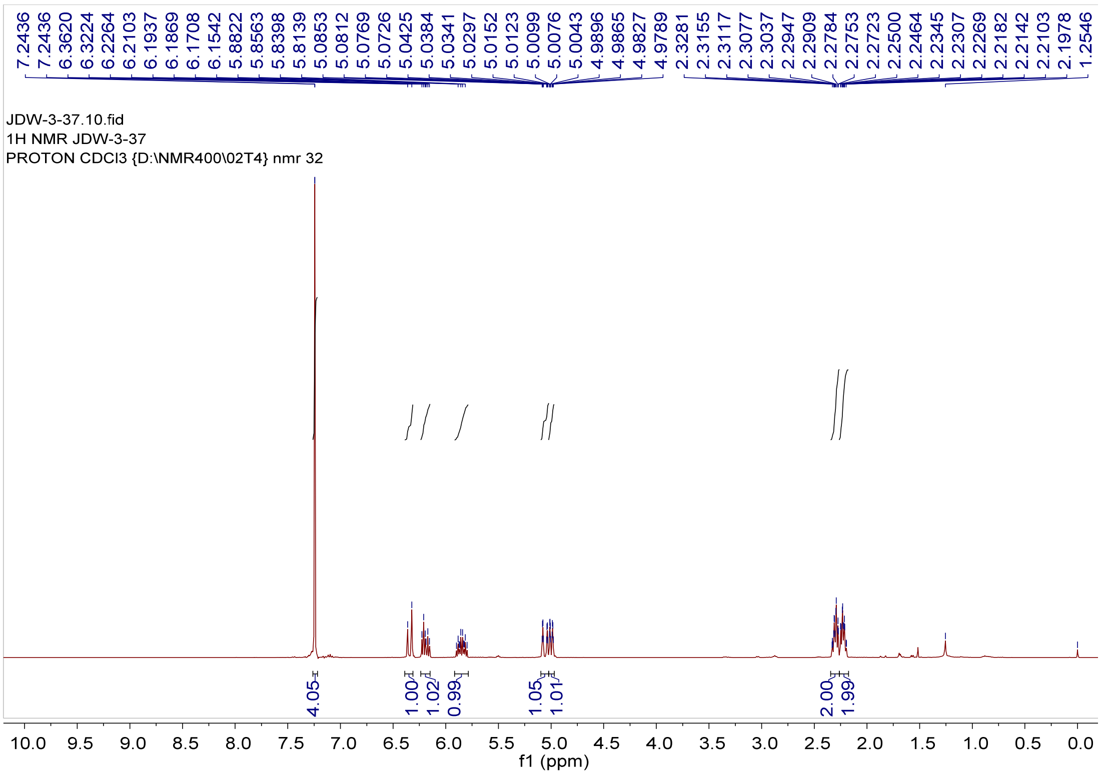

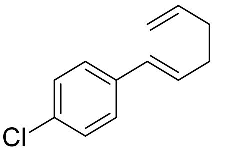

3f ^{1}H NMR (400 MHz, $CDCl_3$)
^{13}C NMR (100 MHz, $CDCl_3$)

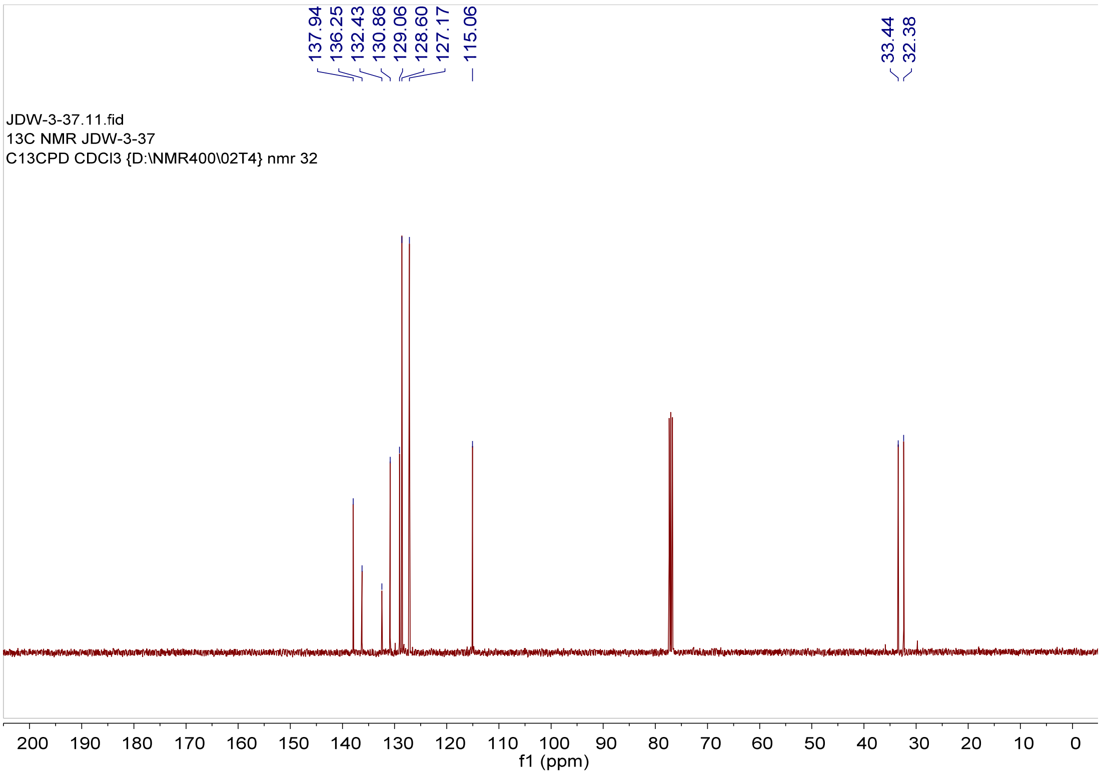

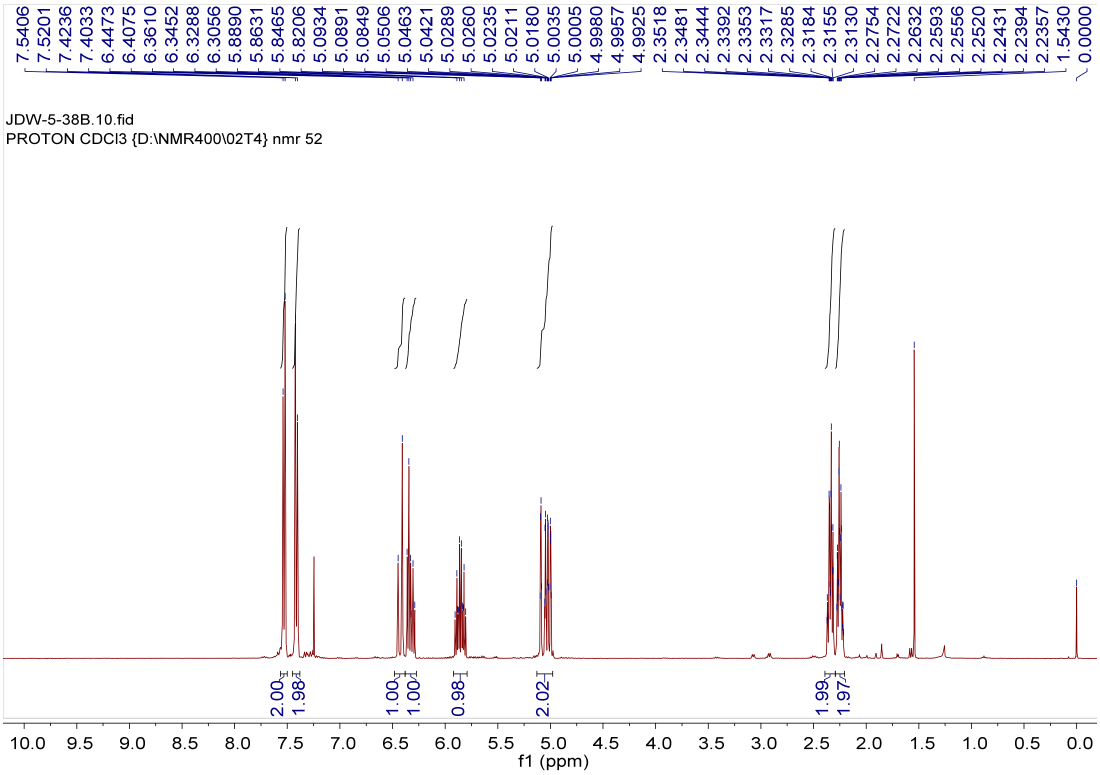

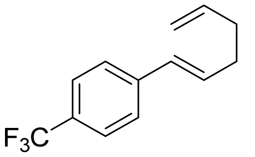

3g ^{1}H NMR (400 MHz, $CDCl_3$)

^{13}C NMR (100 MHz, $CDCl_3$)

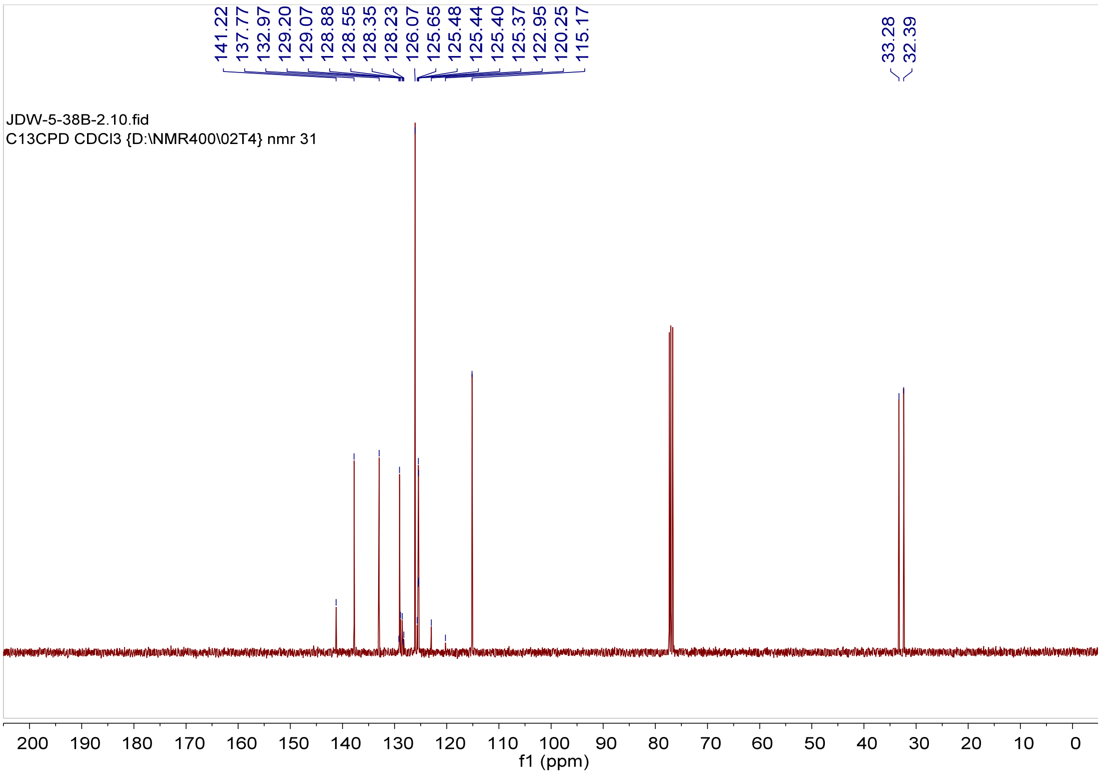

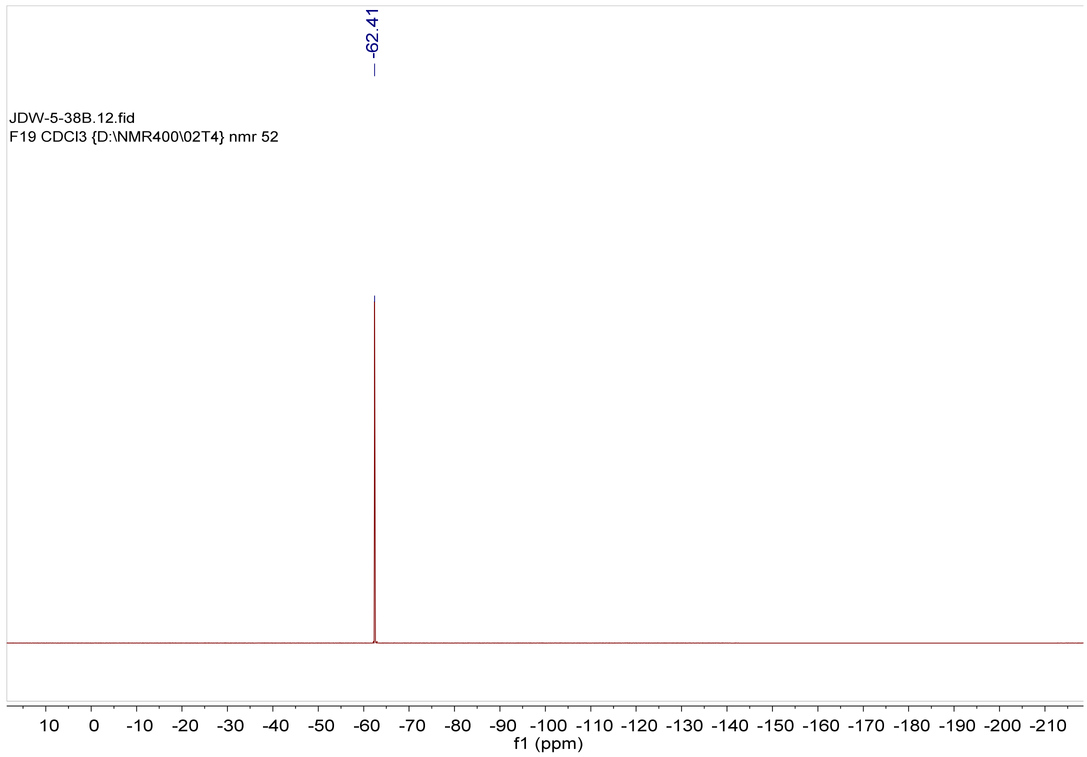

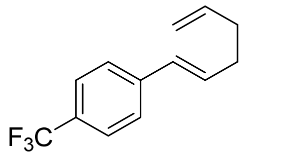

3g ^{19}F NMR (376 MHz, $CDCl_3$)

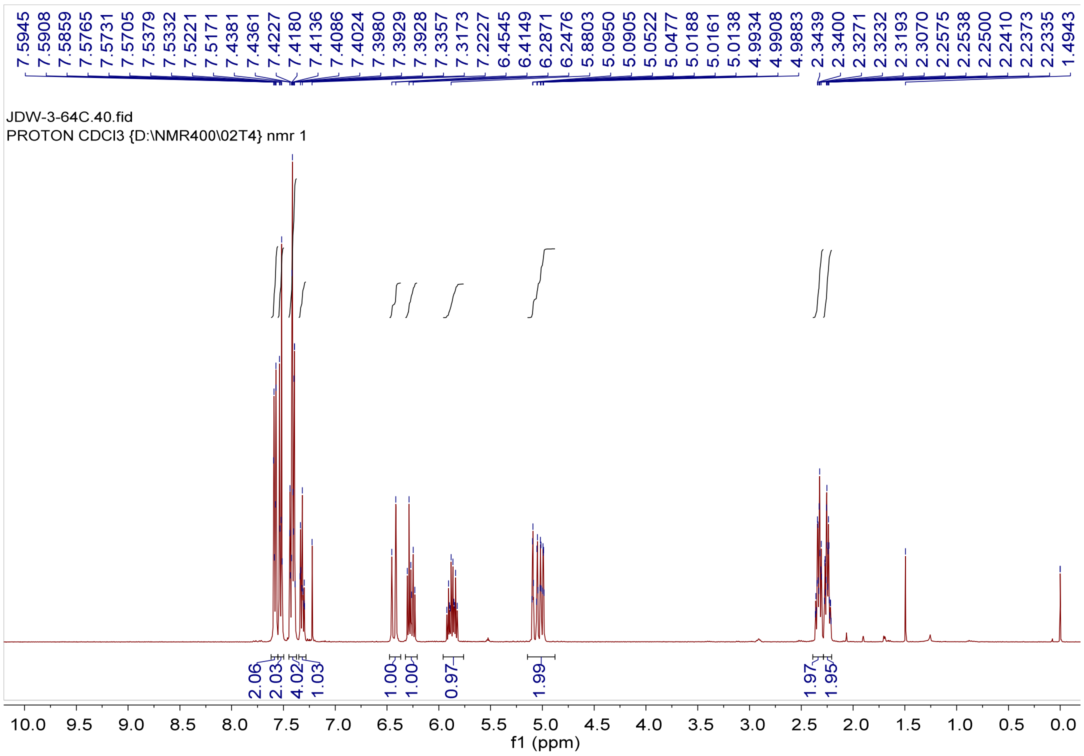

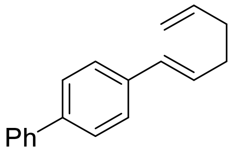

3h ^{1}H NMR (400 MHz, $CDCl_3$)
^{13}C NMR (100 MHz, $CDCl_3$)

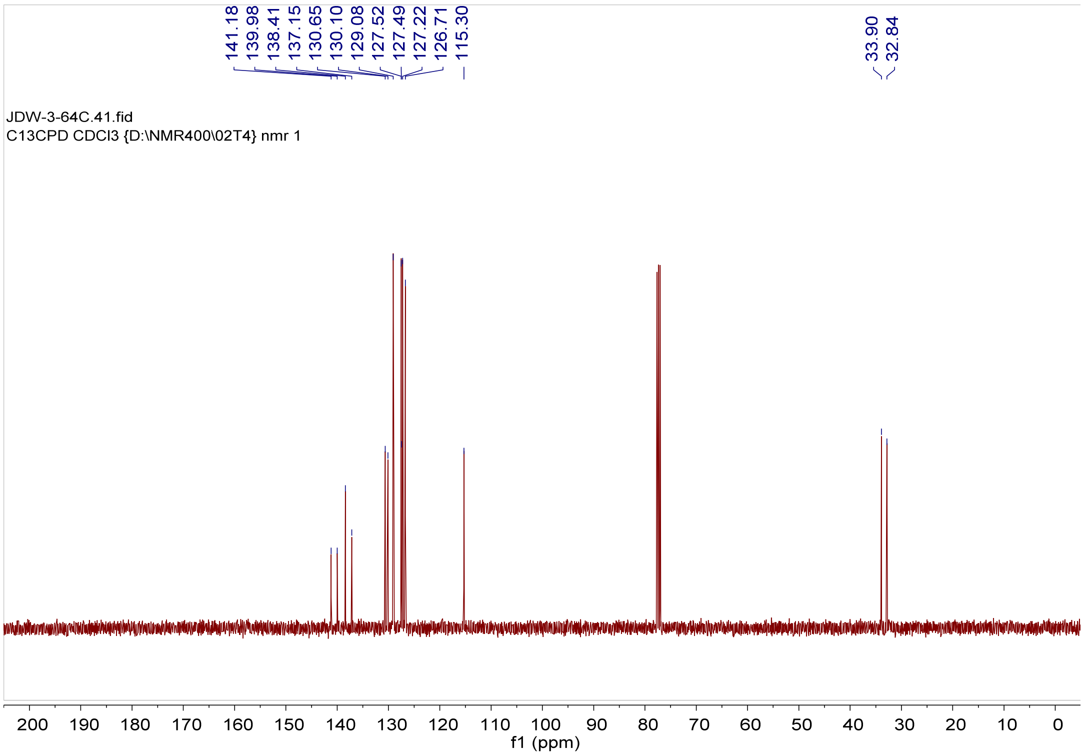

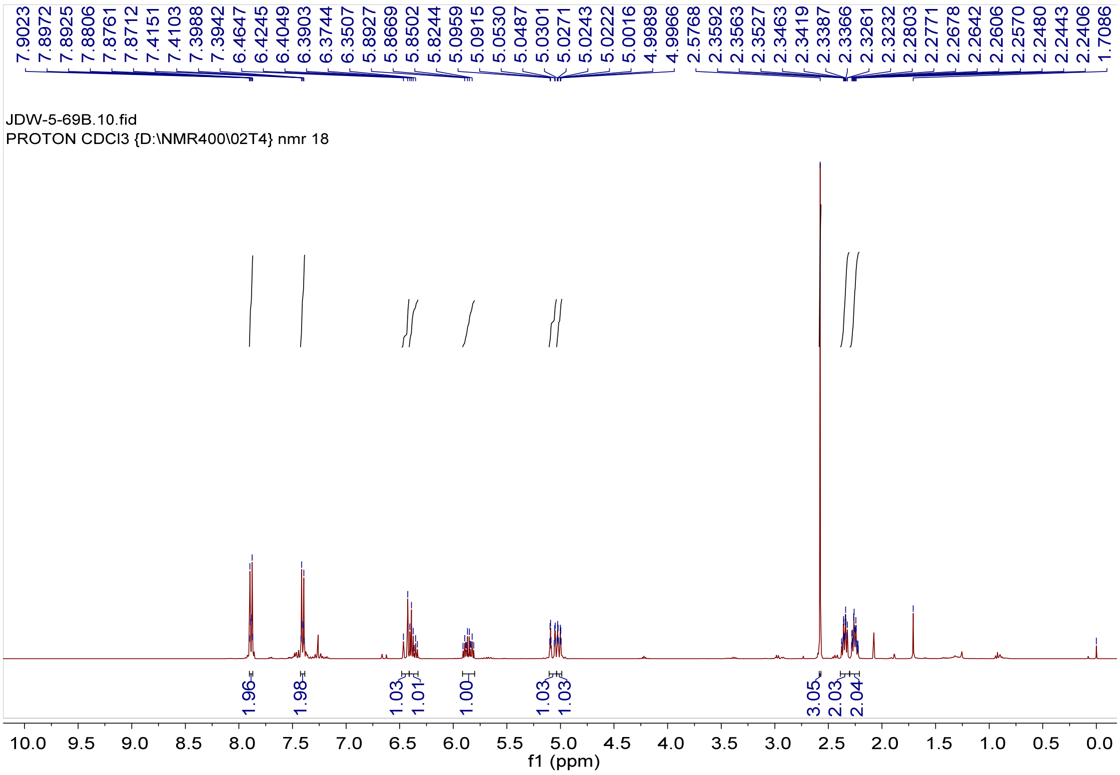

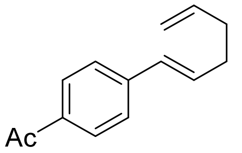

3i ^{1}H NMR (400 MHz, $CDCl_3$)
^{13}C NMR (100 MHz, $CDCl_3$)

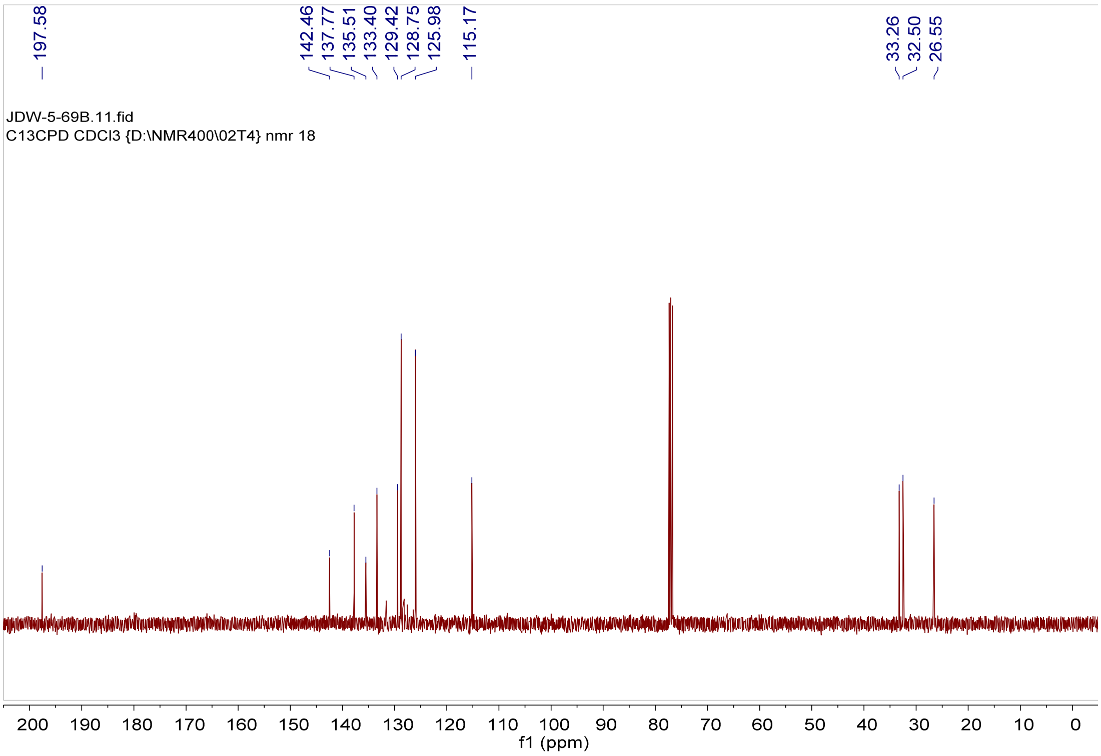

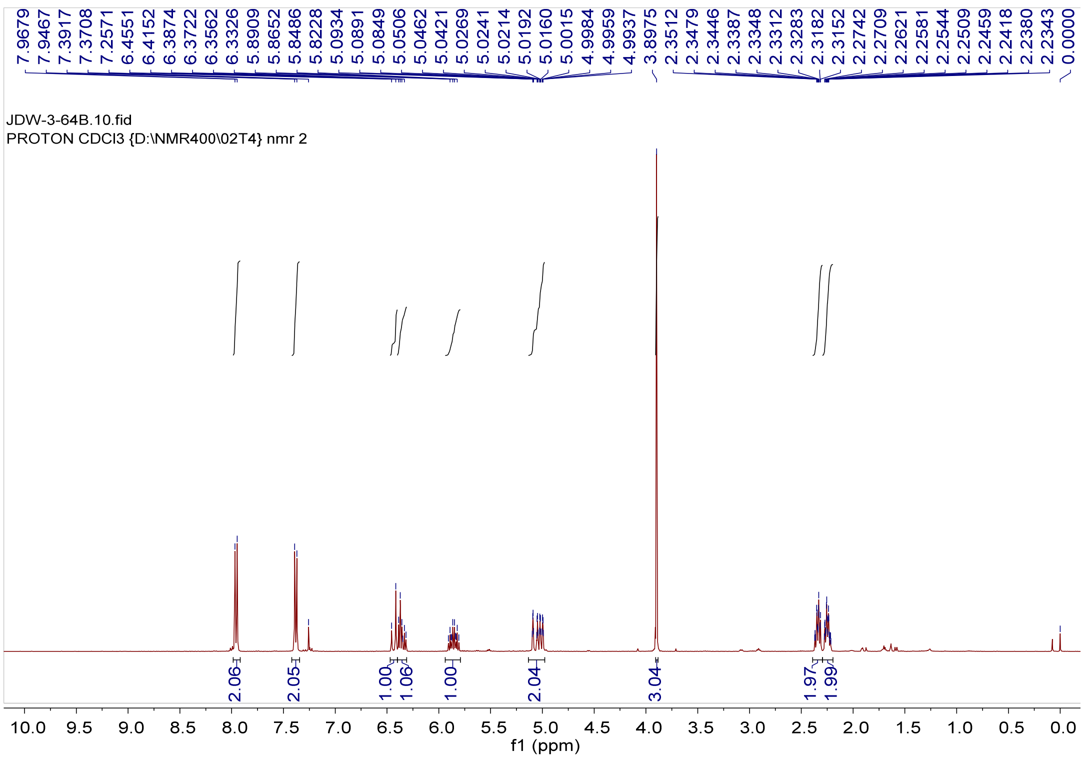

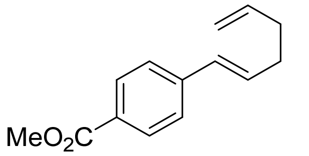

3j ^{1}H NMR (400 MHz, $CDCl_3$)
^{13}C NMR (100 MHz, $CDCl_3$)

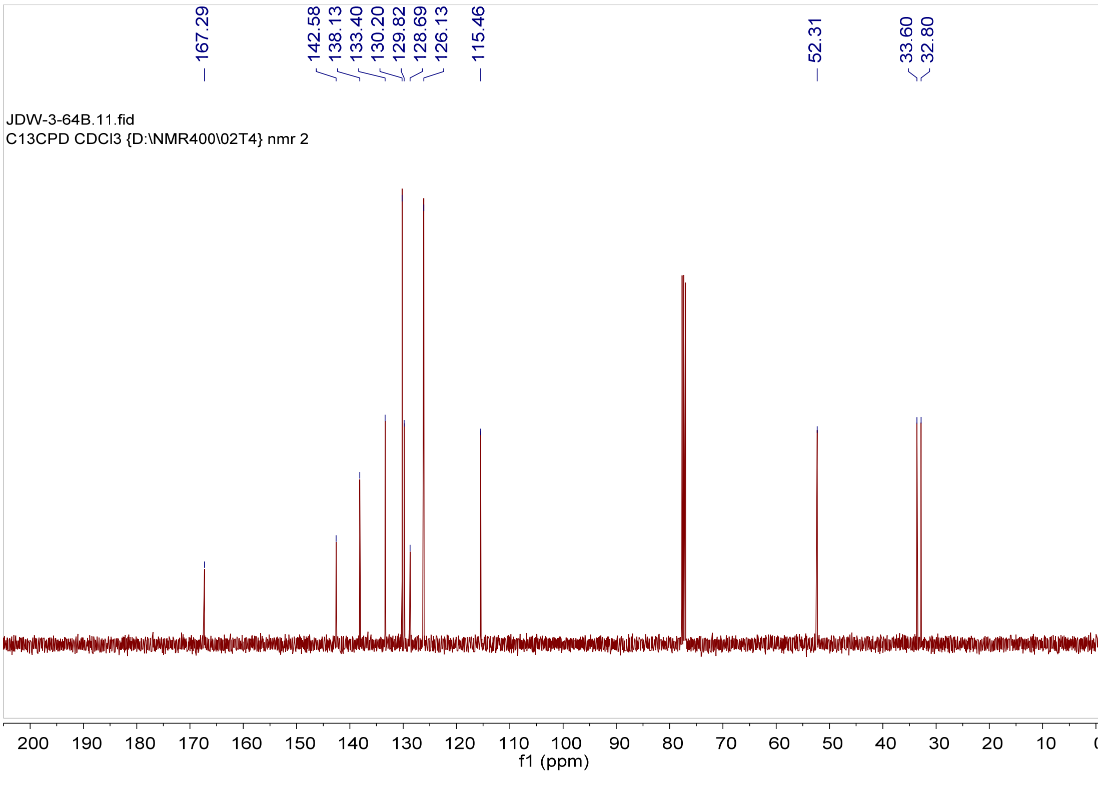

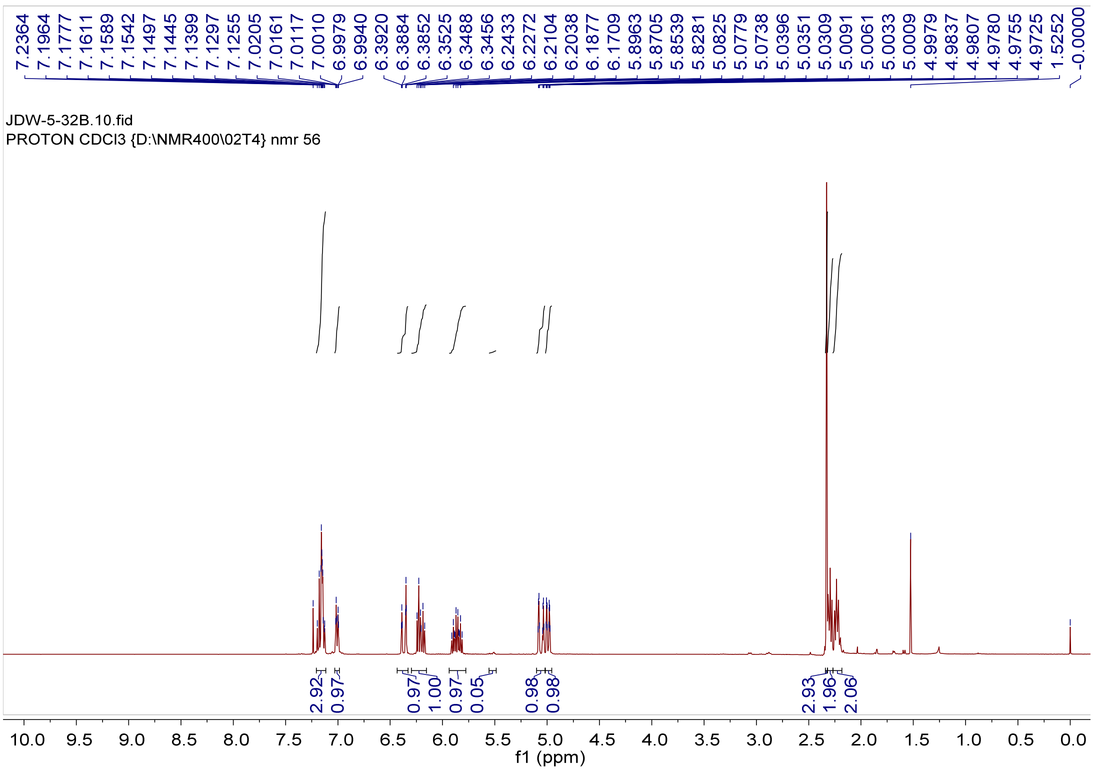

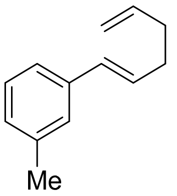

3k ^{1}H NMR (400 MHz, $CDCl_3$)

^{13}C NMR (100 MHz, $CDCl_3$)

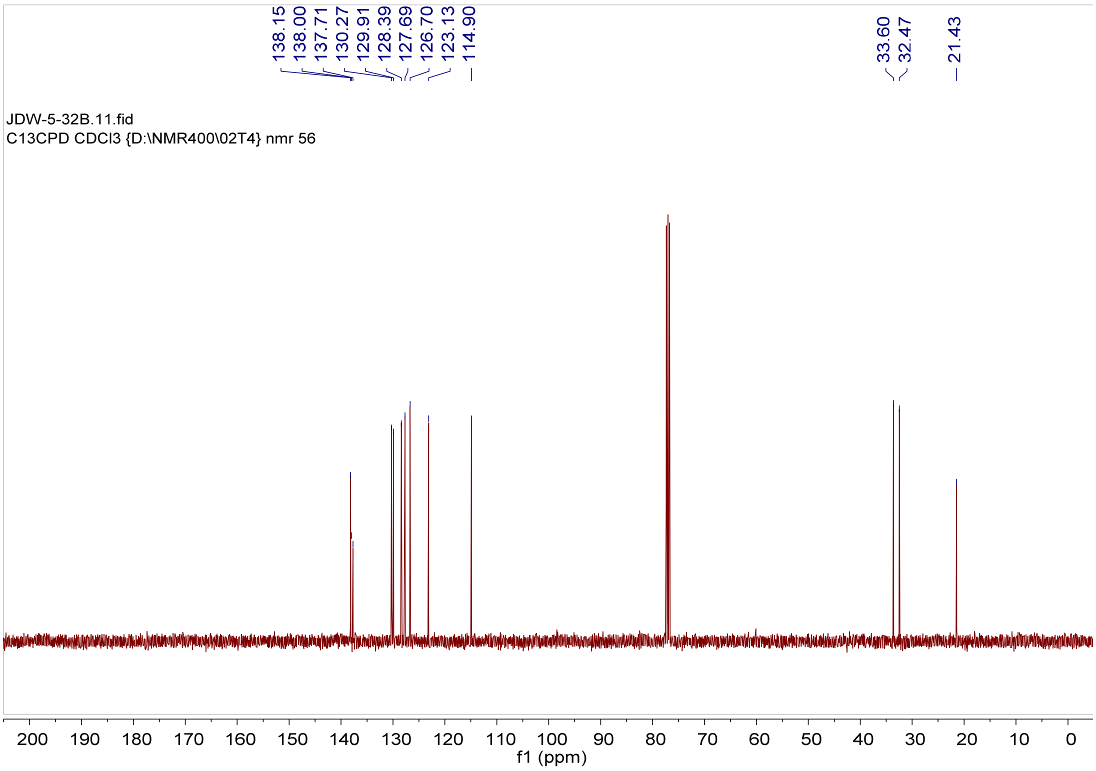

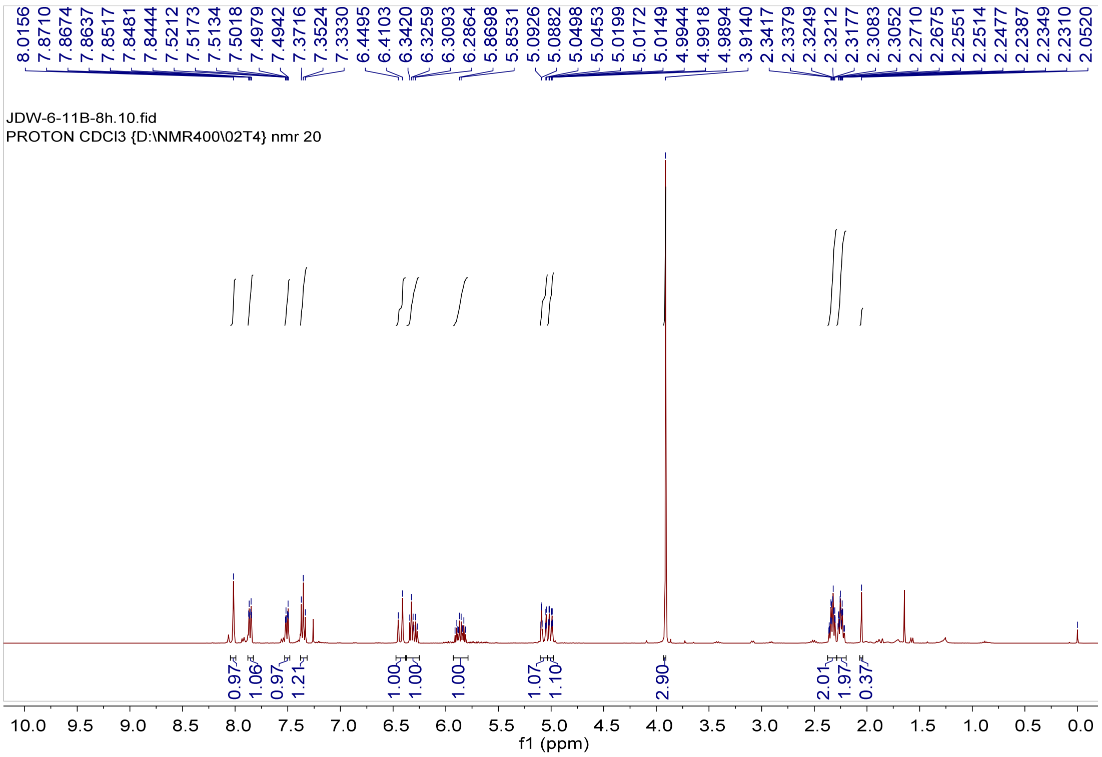

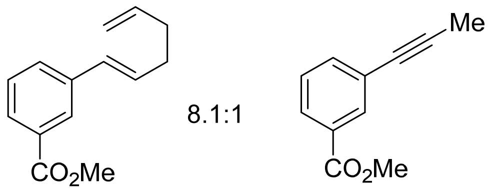

3l ^{1}H NMR (400 MHz, $CDCl_3$)

^{13}C NMR (100 MHz, $CDCl_3$)

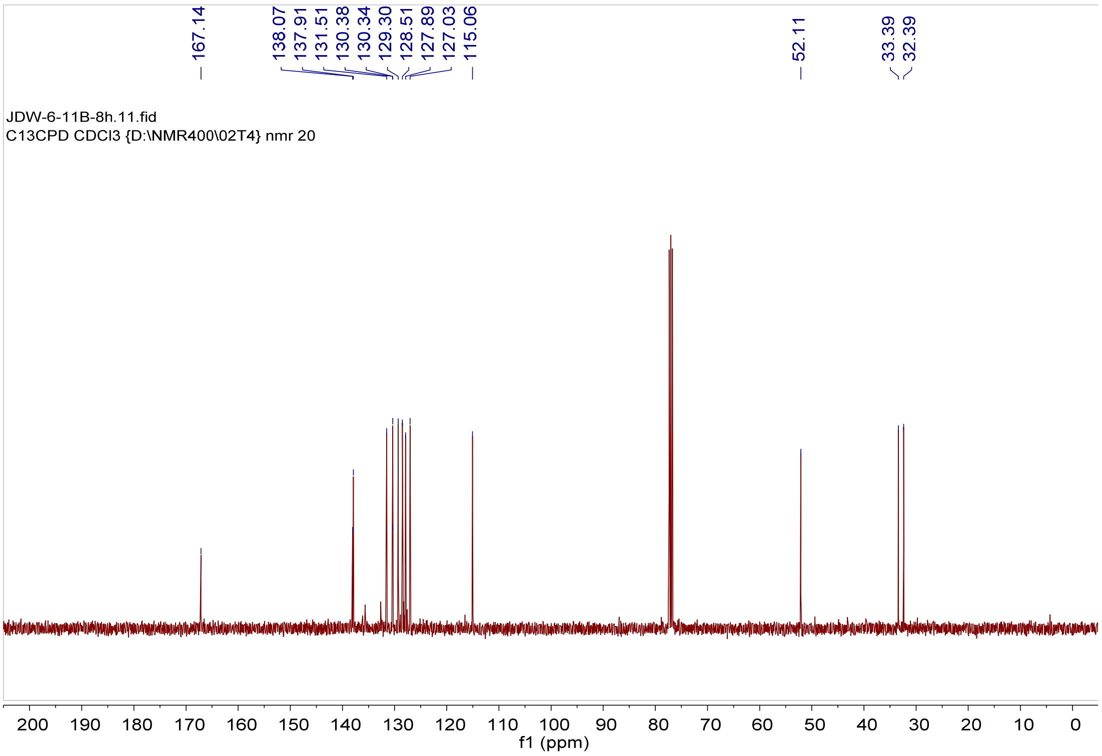

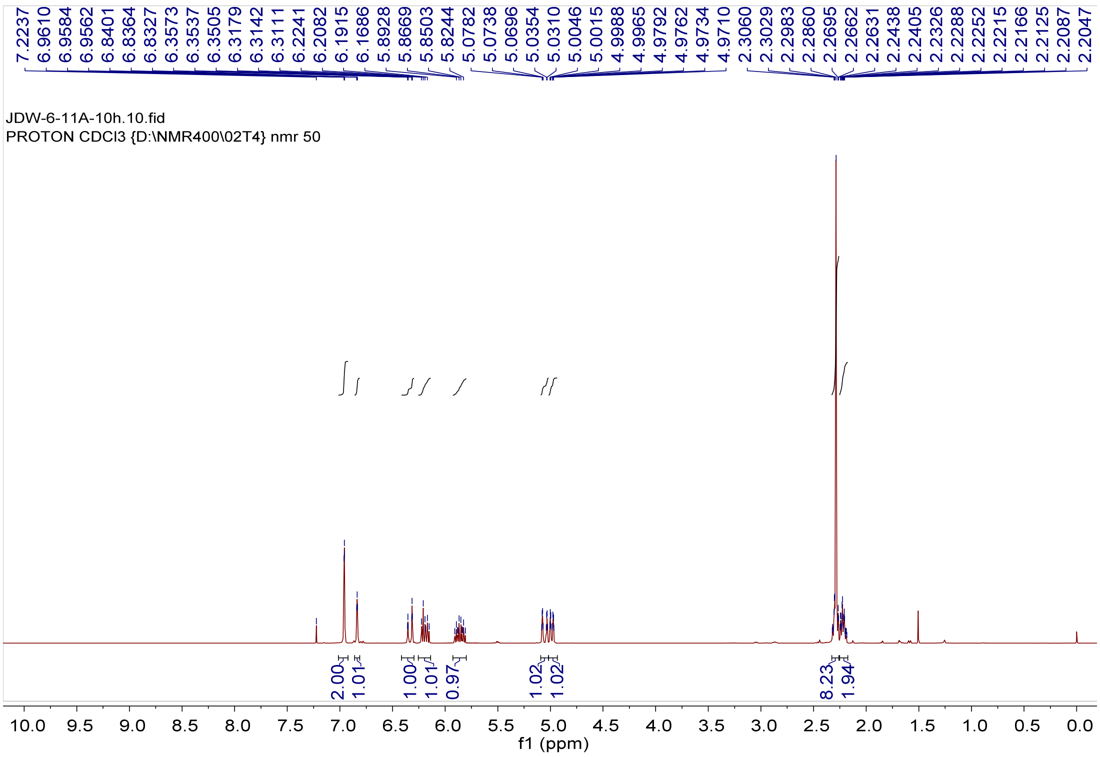

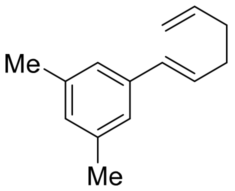

3m ^{1}H NMR (400 MHz, $CDCl_3$)
^{13}C NMR (100 MHz, $CDCl_3$)

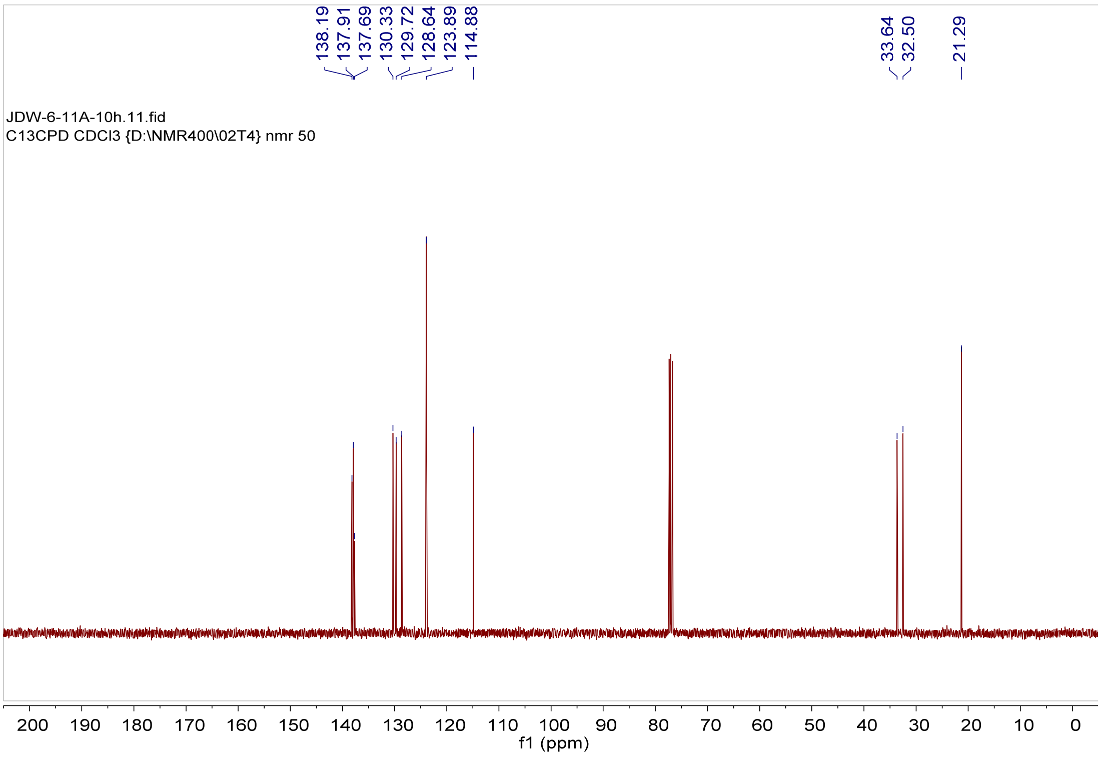

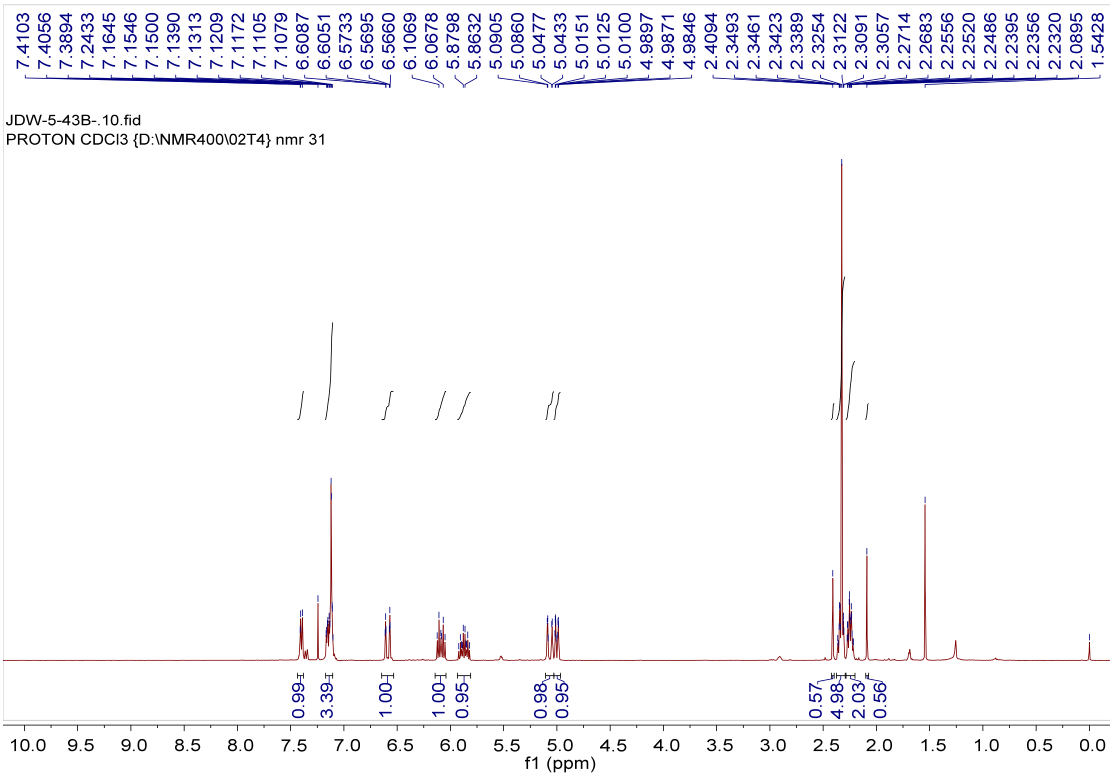

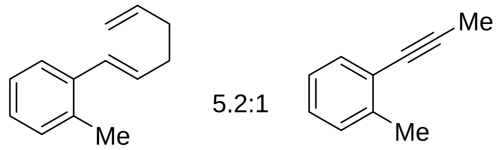

3n ^{1}H NMR (400 MHz, $CDCl_3$)
^{13}C NMR (100 MHz, $CDCl_3$)

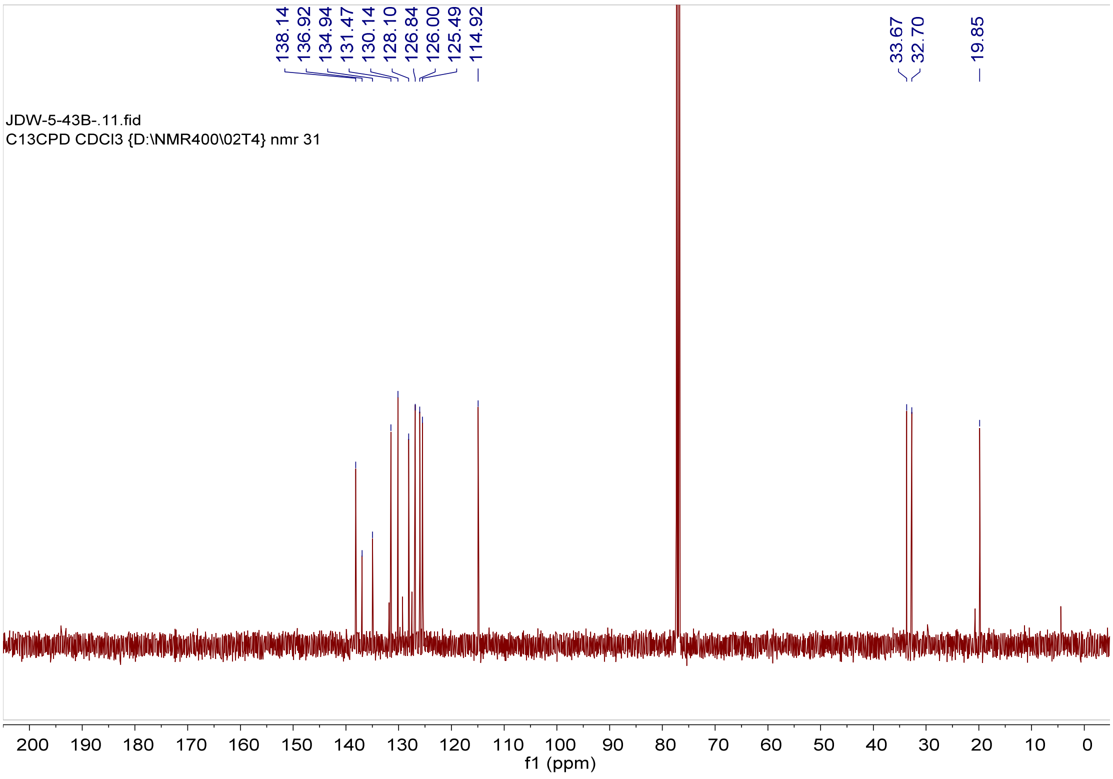

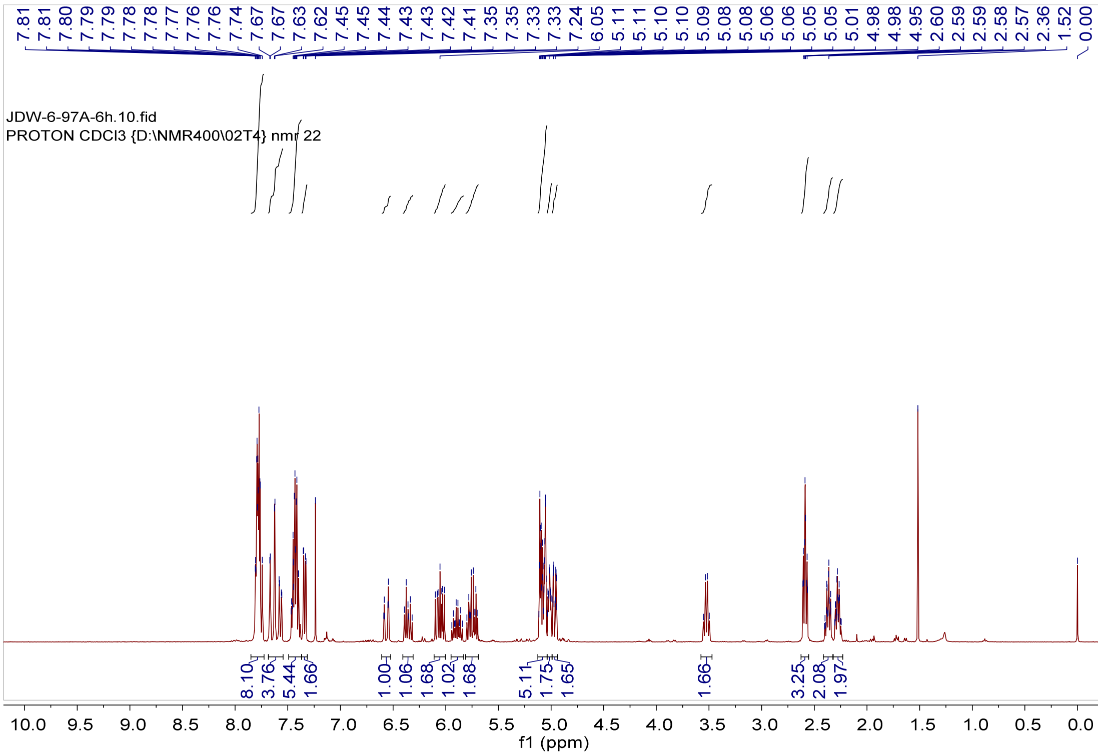

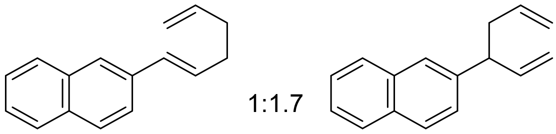

3o/3o' ^{1}H NMR (400 MHz, $CDCl_3$)

^{13}C NMR (100 MHz, $CDCl_3$)

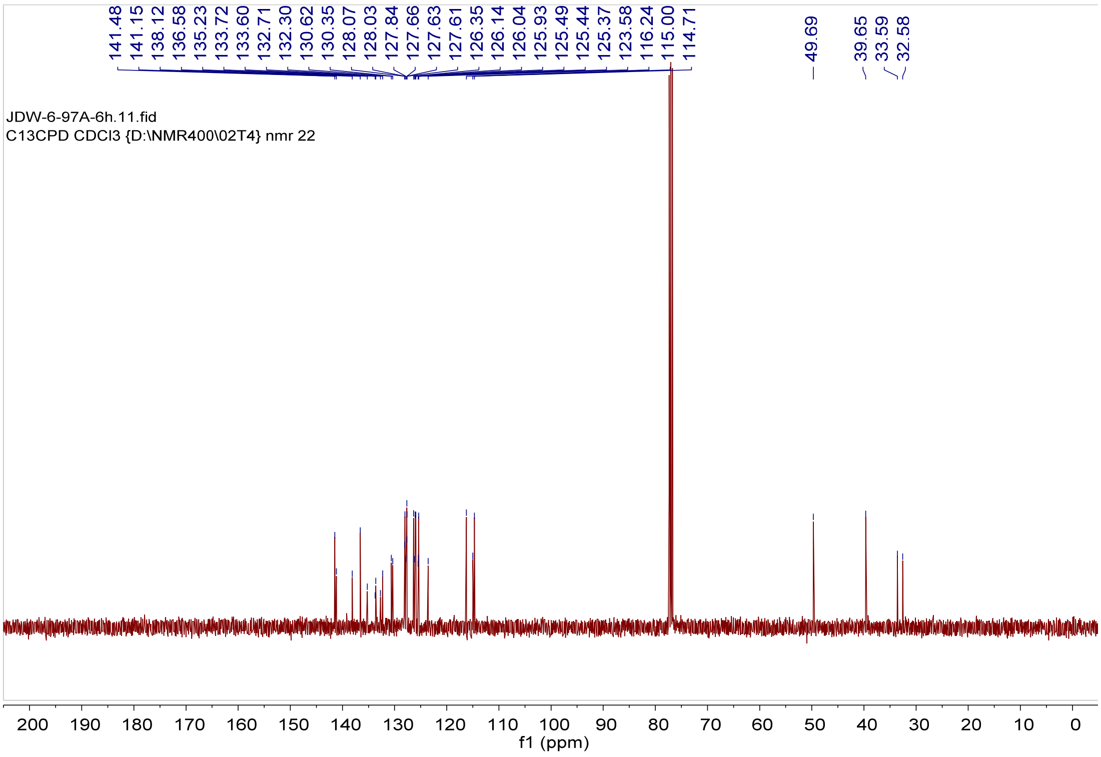

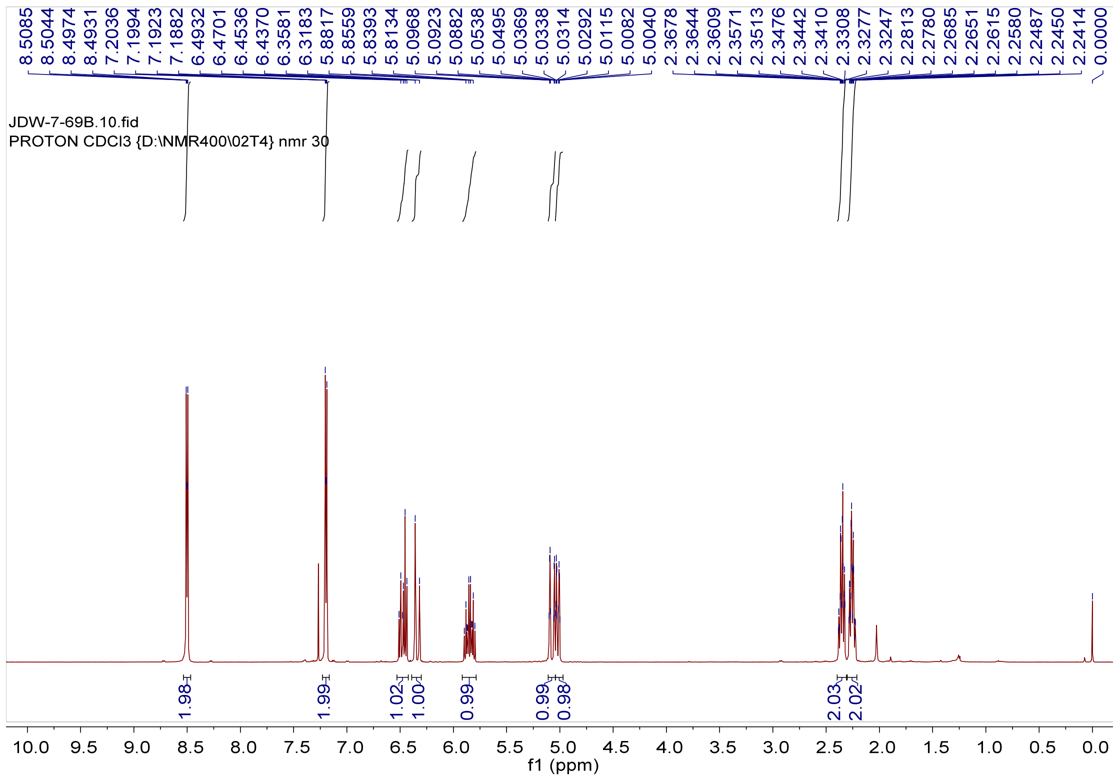

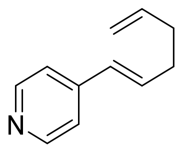

3r ^{1}H NMR (400 MHz, $CDCl_3$)
^{13}C NMR (100 MHz, $CDCl_3$)

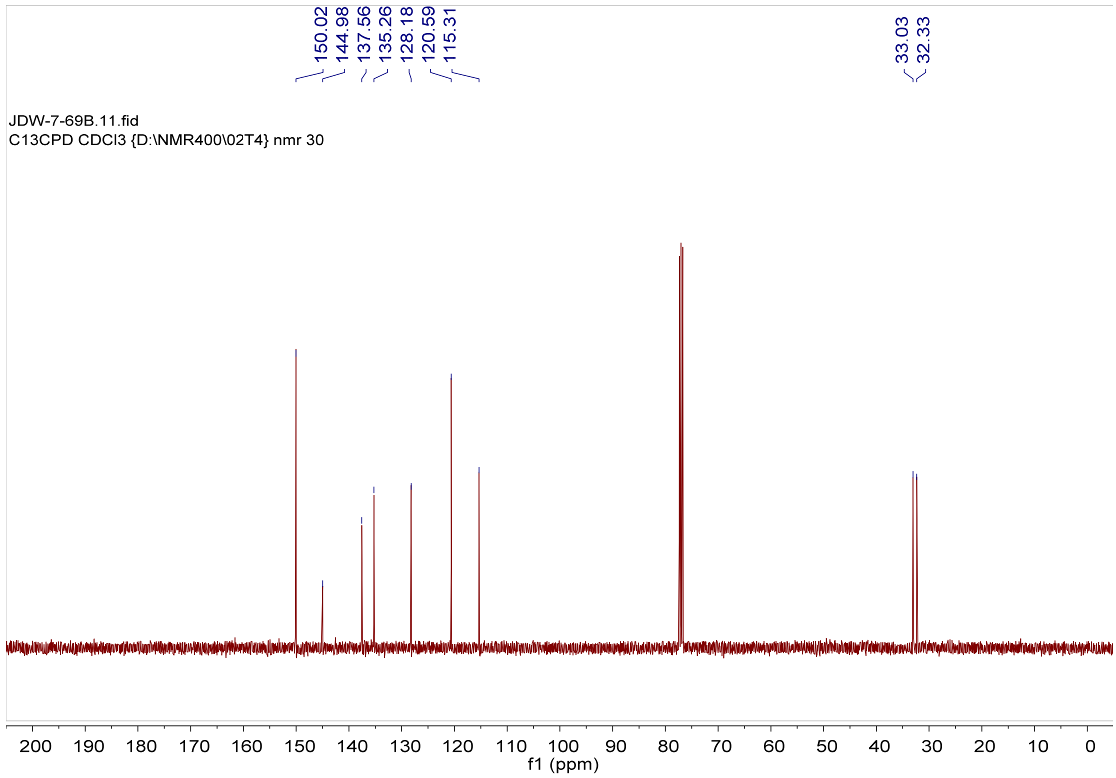

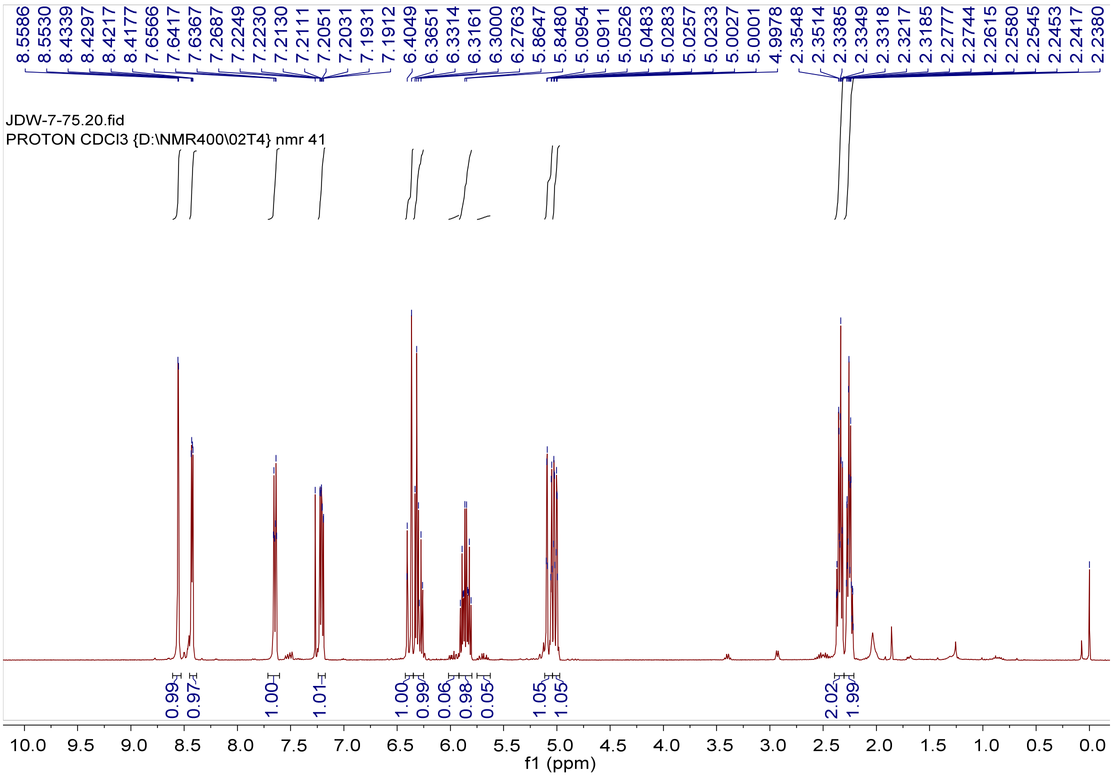

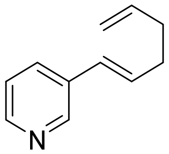

3s ^1H NMR (400 MHz, $CDCl_3$)
^{13}C NMR (100 MHz, $CDCl_3$)

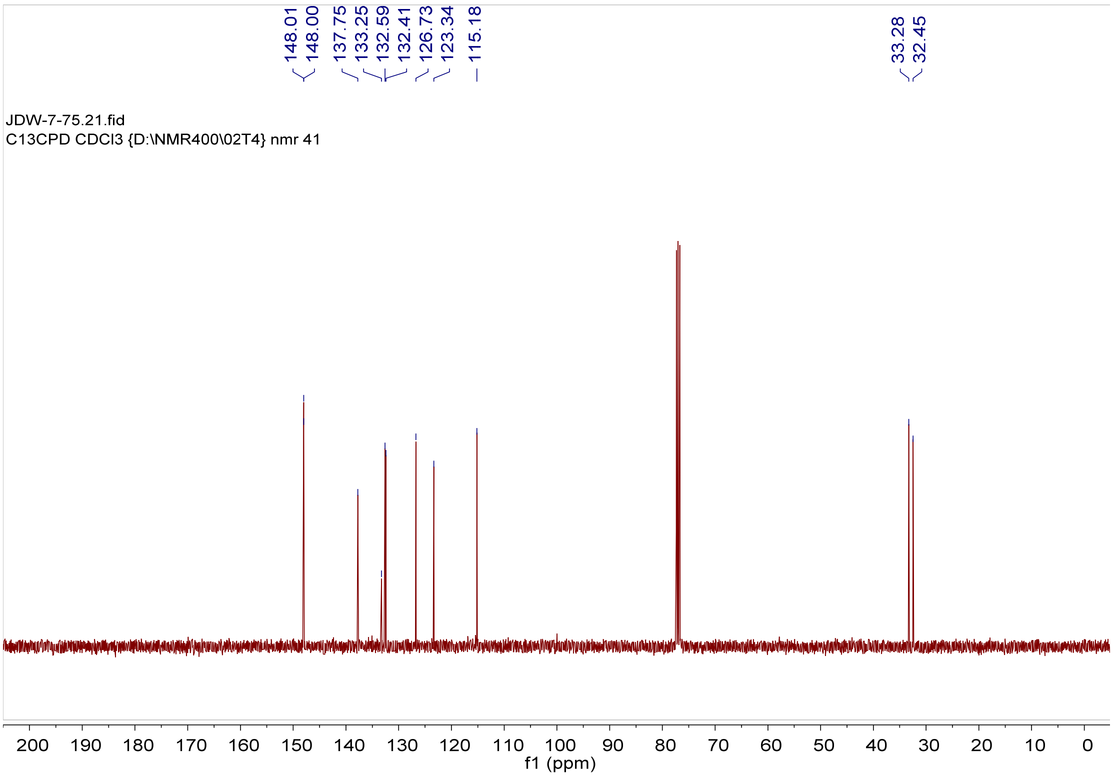

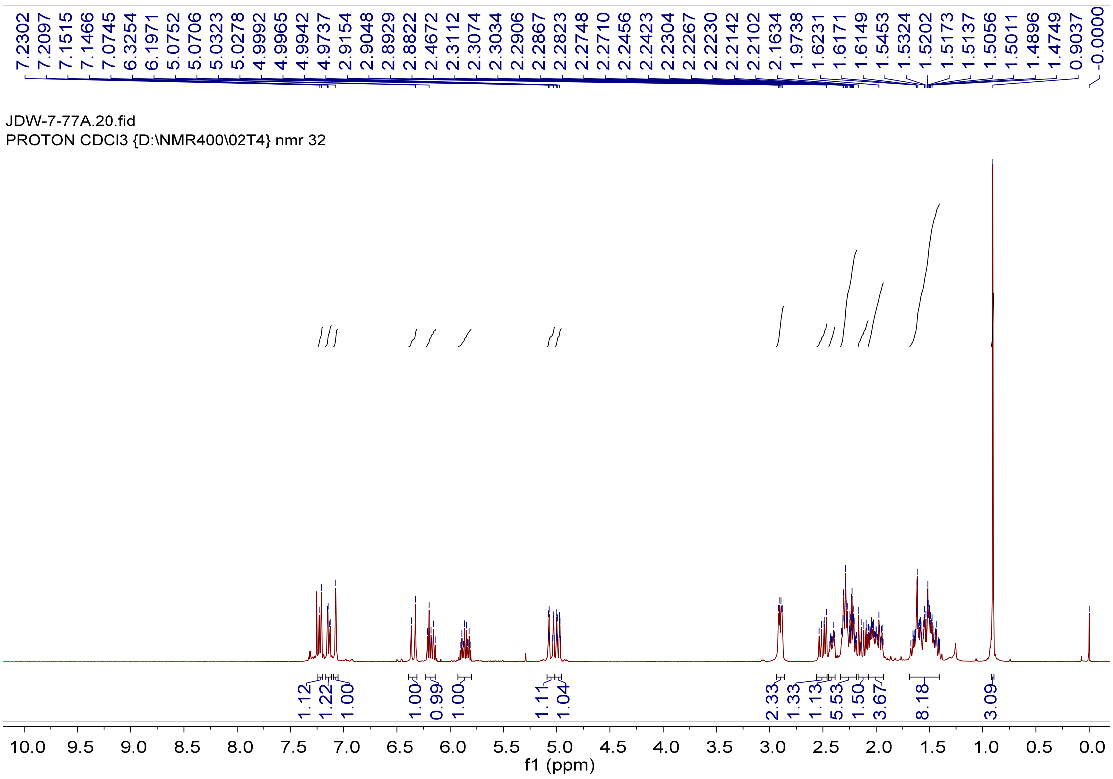

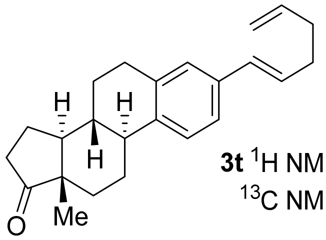

3t ^{1}H NMR (400 MHz, $CDCl_3$)
^{13}C NMR (100 MHz, $CDCl_3$)

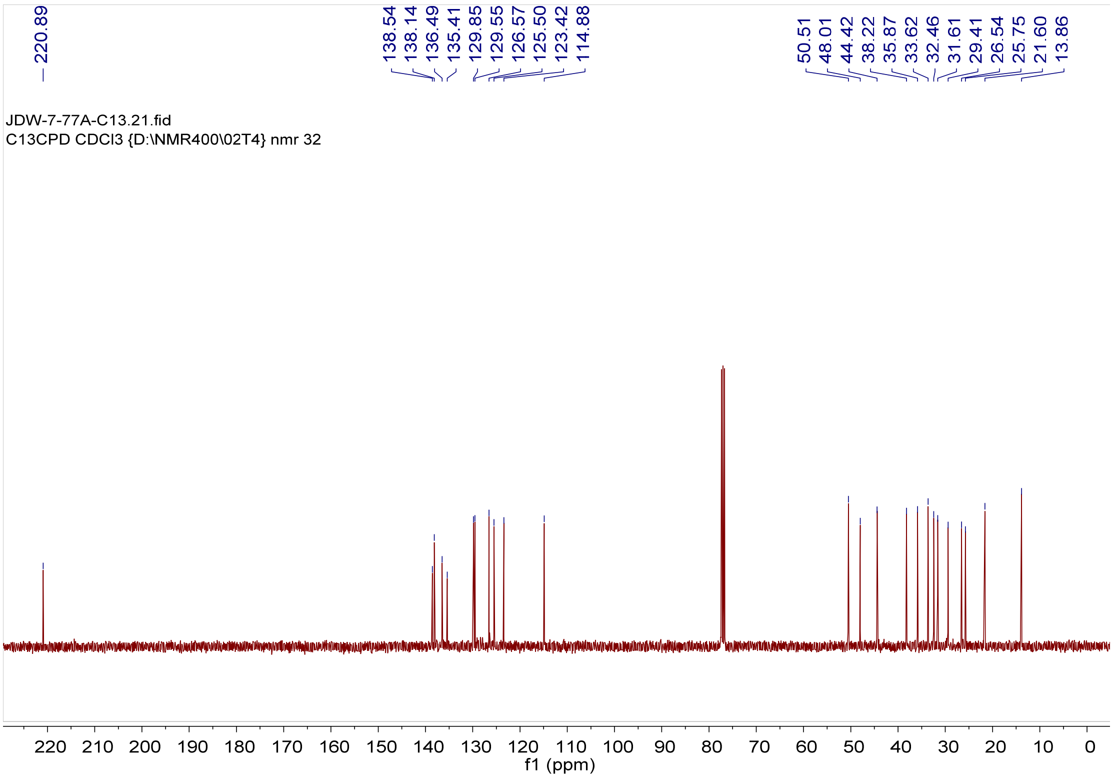

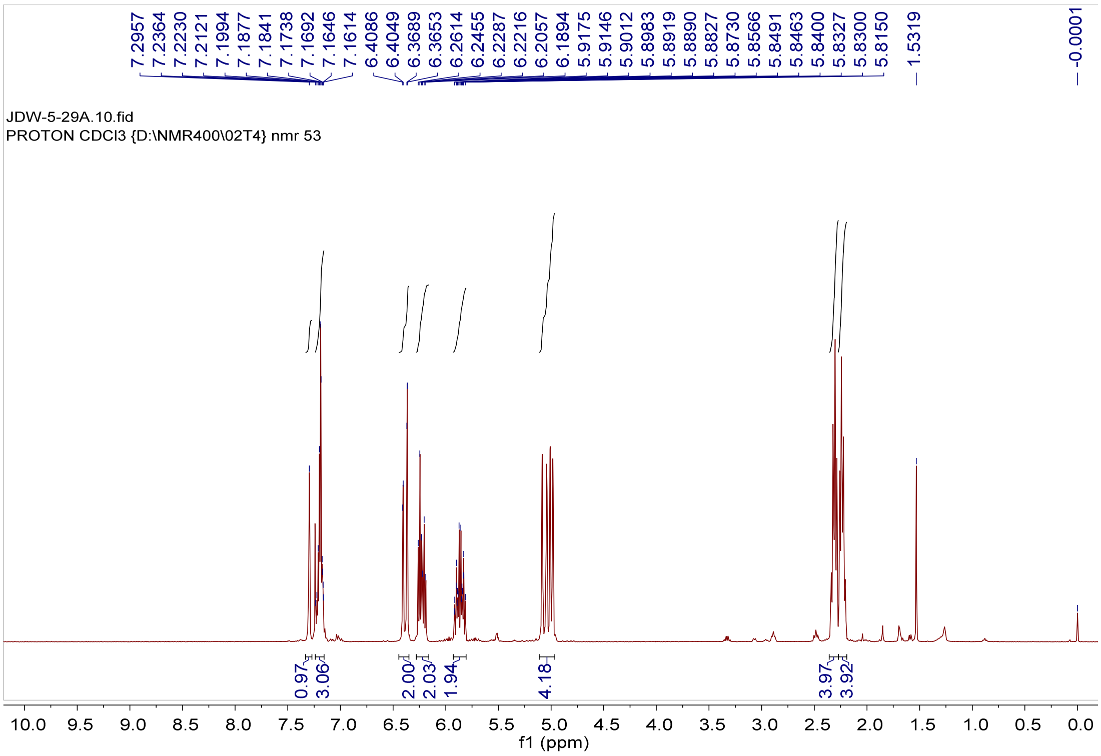

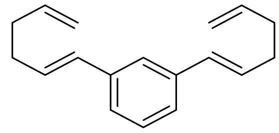

3v ^{1}H NMR (400 MHz, $CDCl_3$)
^{13}C NMR (100 MHz, $CDCl_3$)

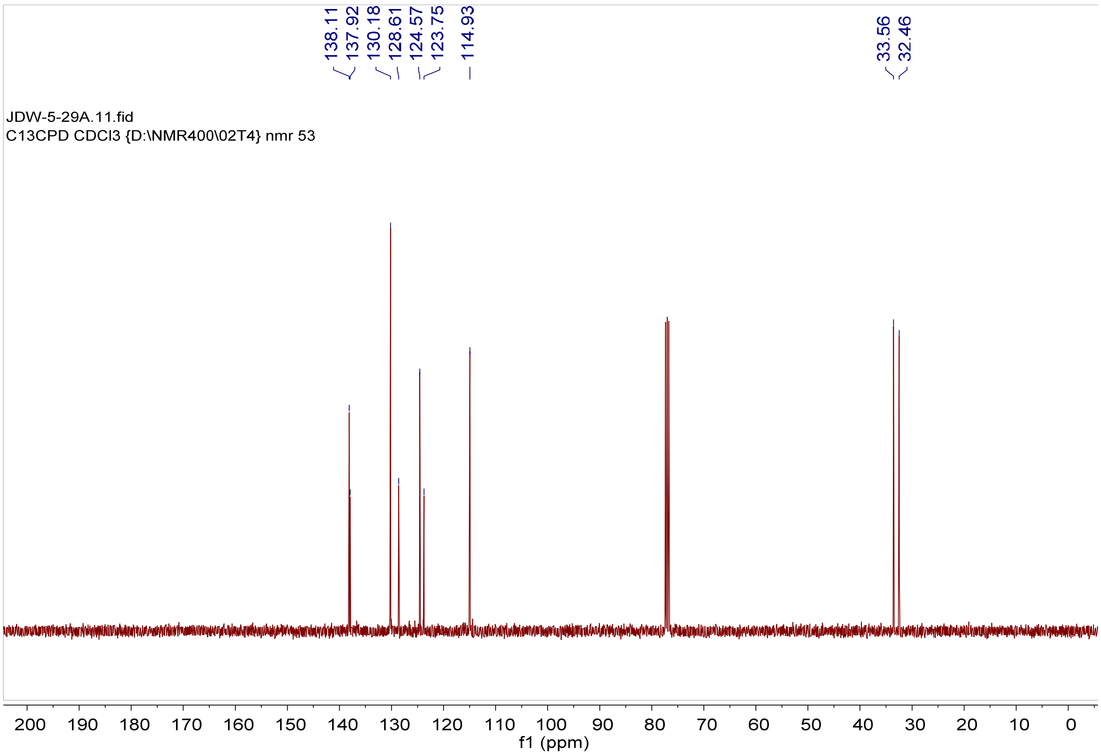

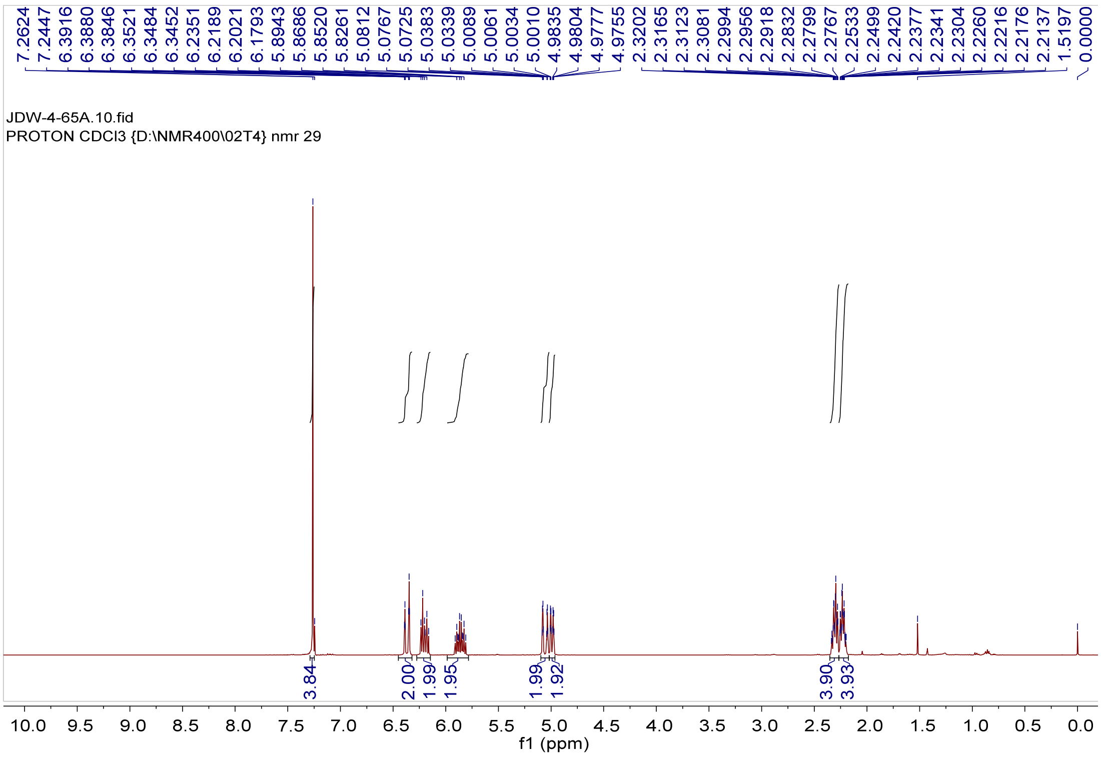

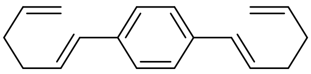

3w ^{1}H NMR (400 MHz, $CDCl_3$)

^{13}C NMR (100 MHz, $CDCl_3$)

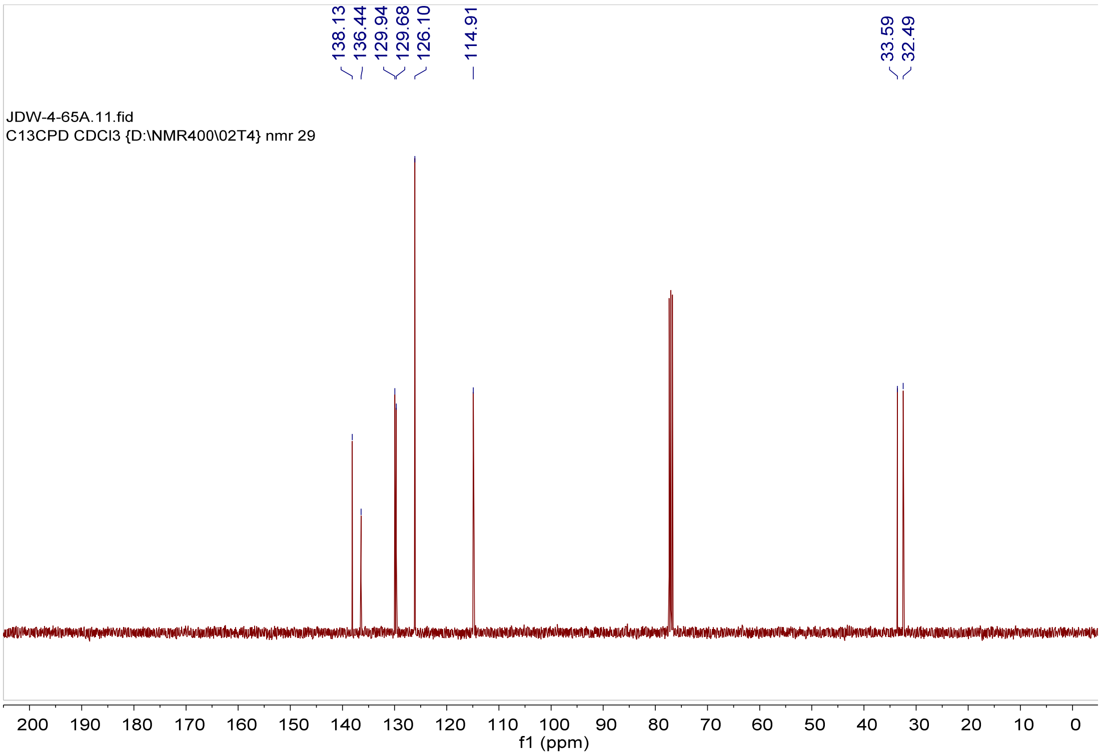

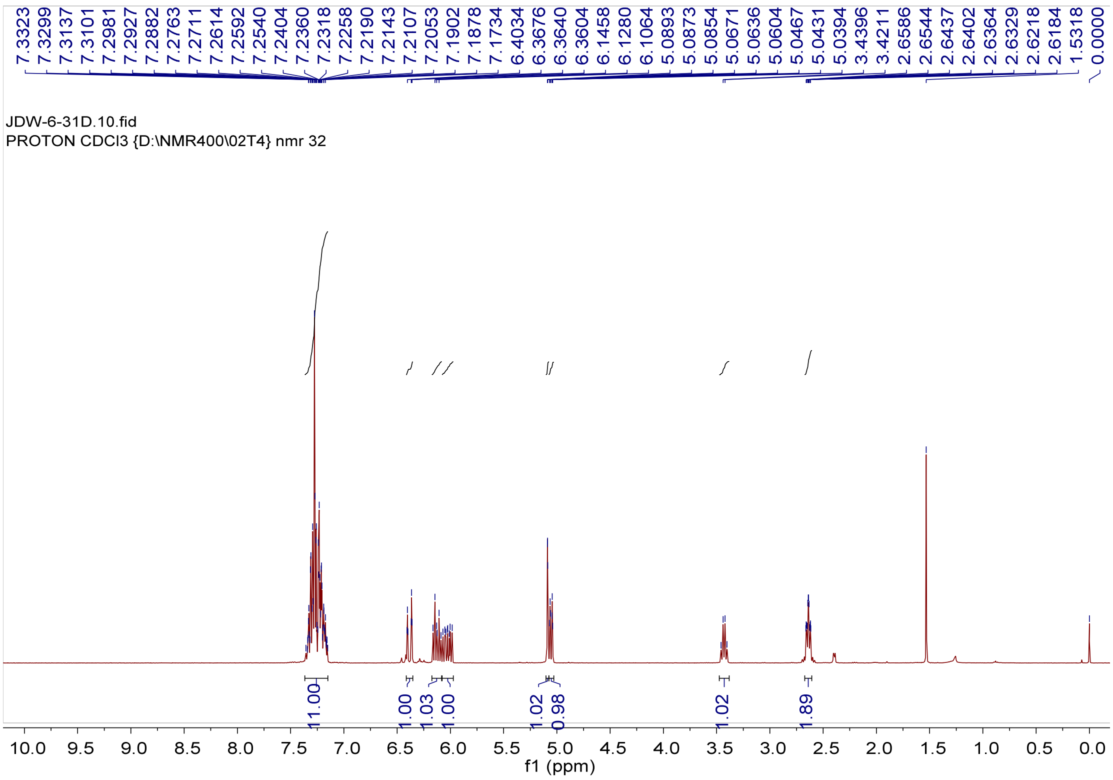

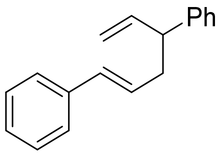

3x ^{1}H NMR (400 MHz, $CDCl_3$)

^{13}C NMR (100 MHz, $CDCl_3$)

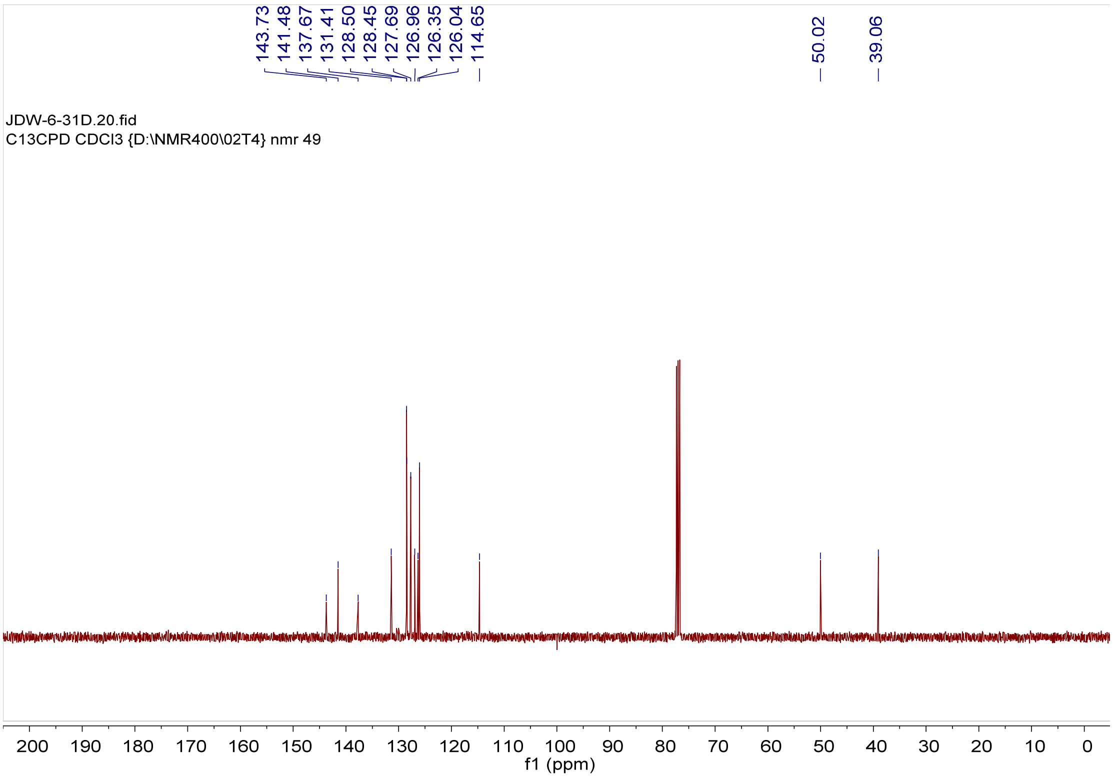

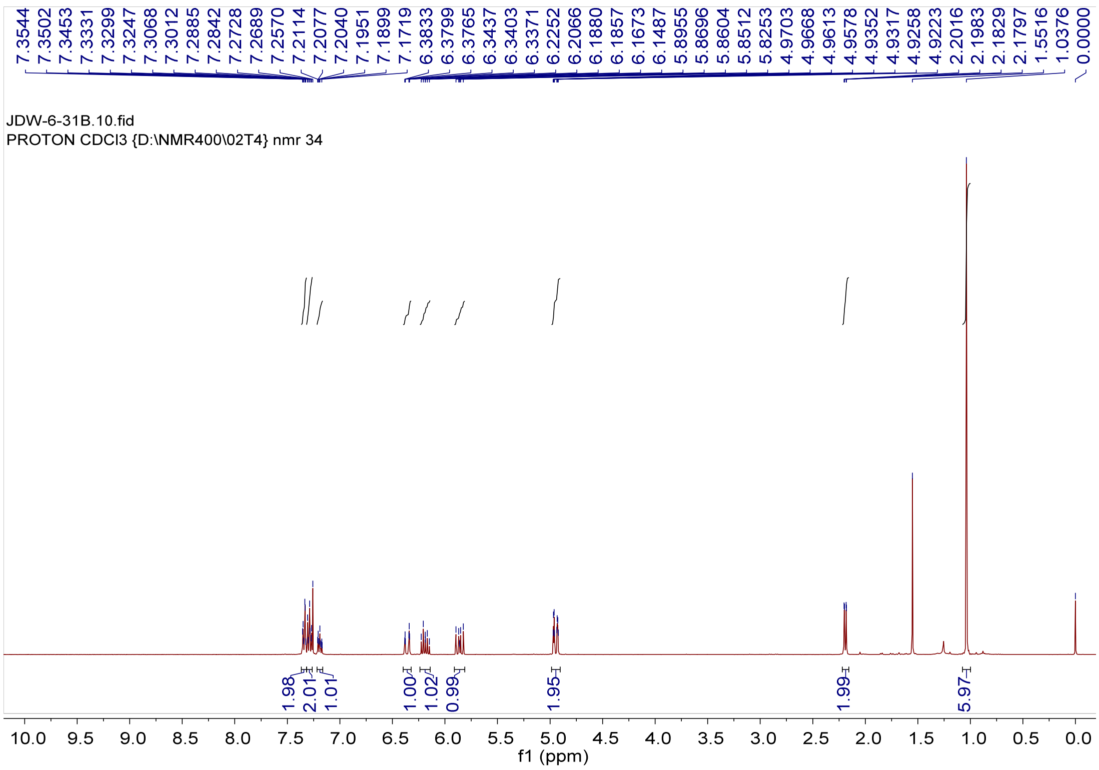

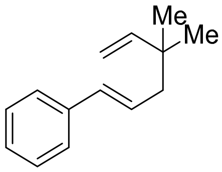

3y ^{1}H NMR (400 MHz, $CDCl_3$)
^{13}C NMR (100 MHz, $CDCl_3$)

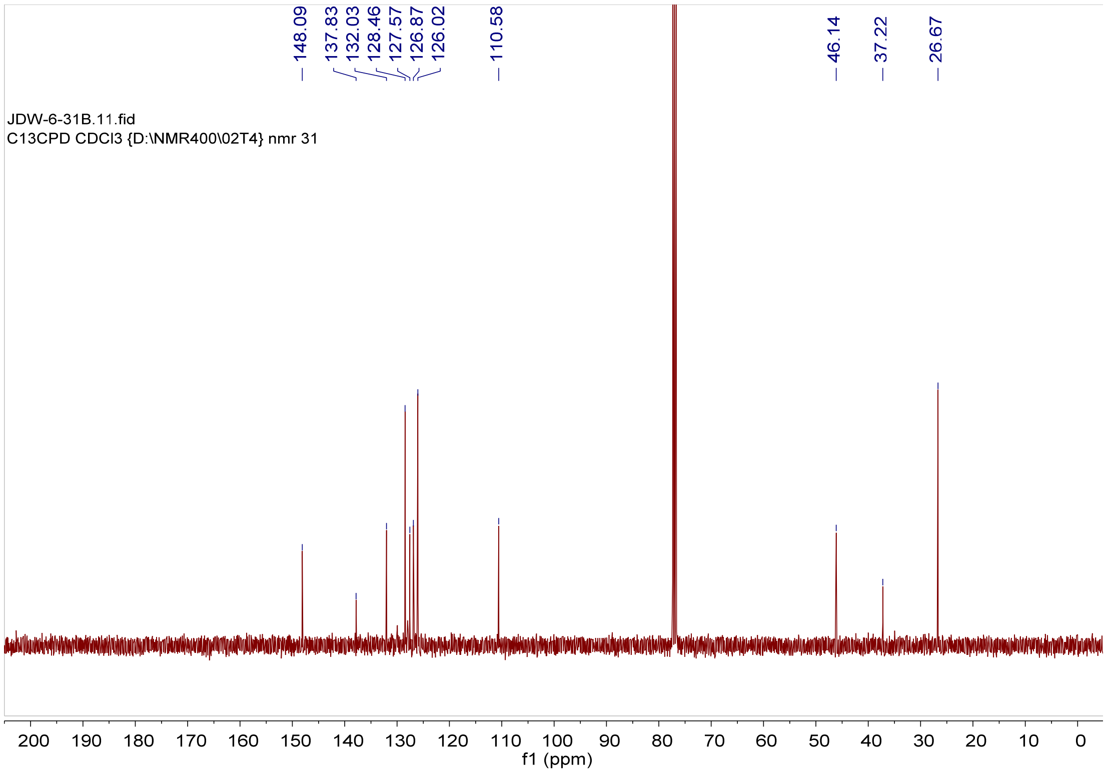

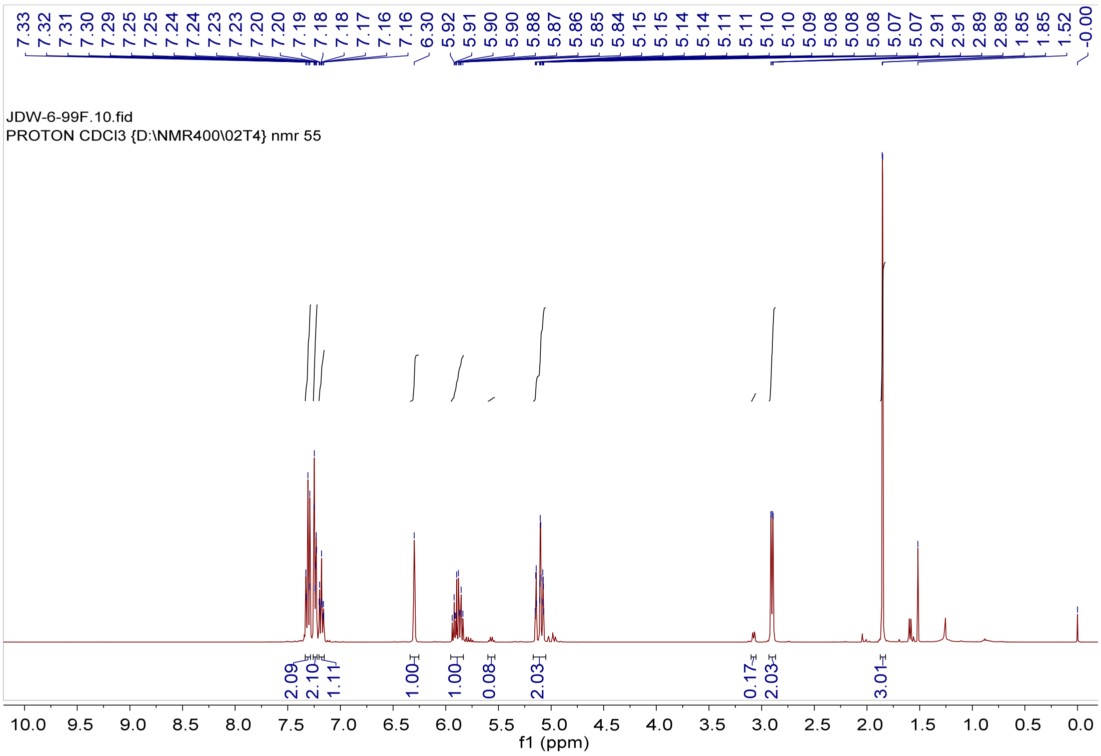

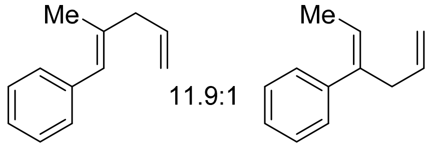

4a/4a' ^{1}H NMR (400 MHz, $CDCl_3$)
^{13}C NMR (100 MHz, $CDCl_3$)

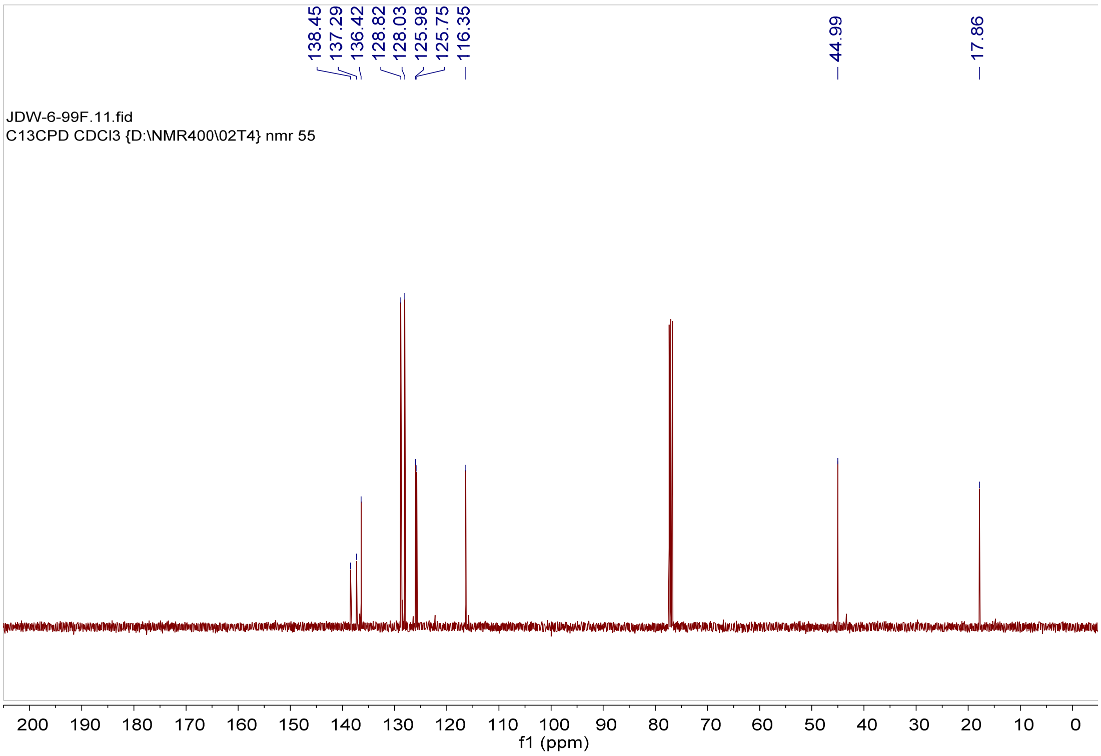

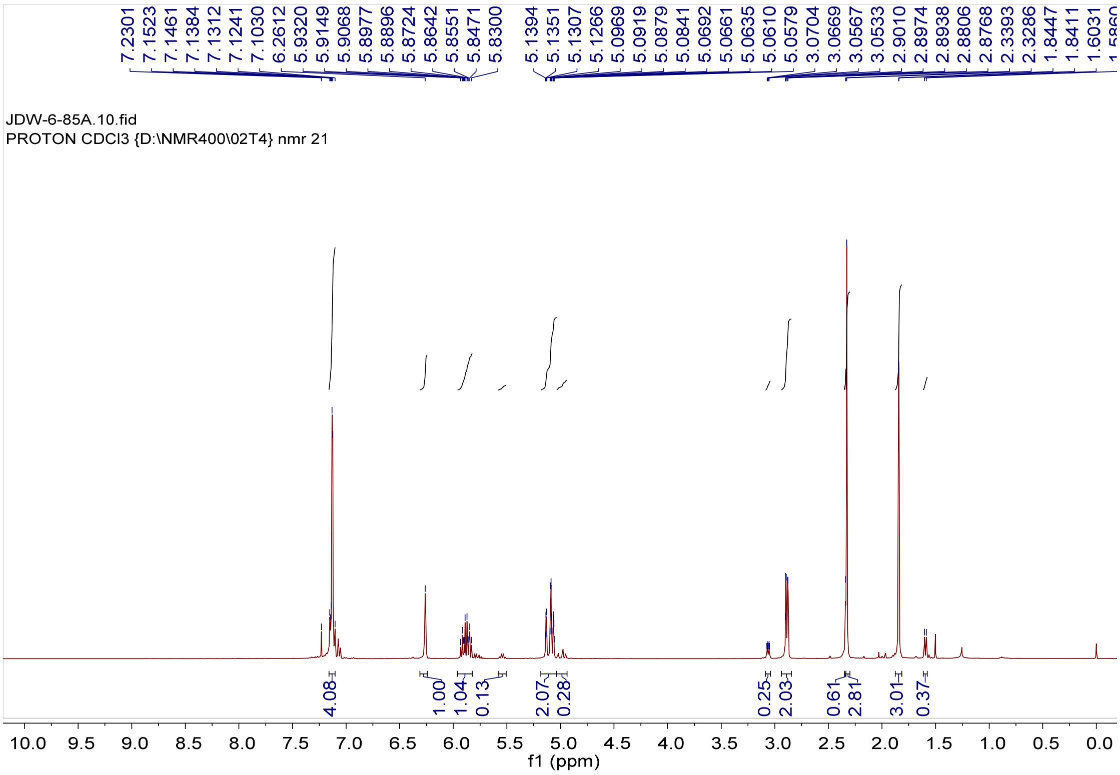

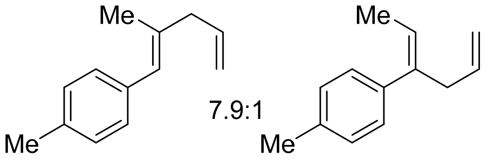

4b/4b' ^{1}H NMR (400 MHz, $CDCl_3$)

^{13}C NMR (100 MHz, $CDCl_3$)

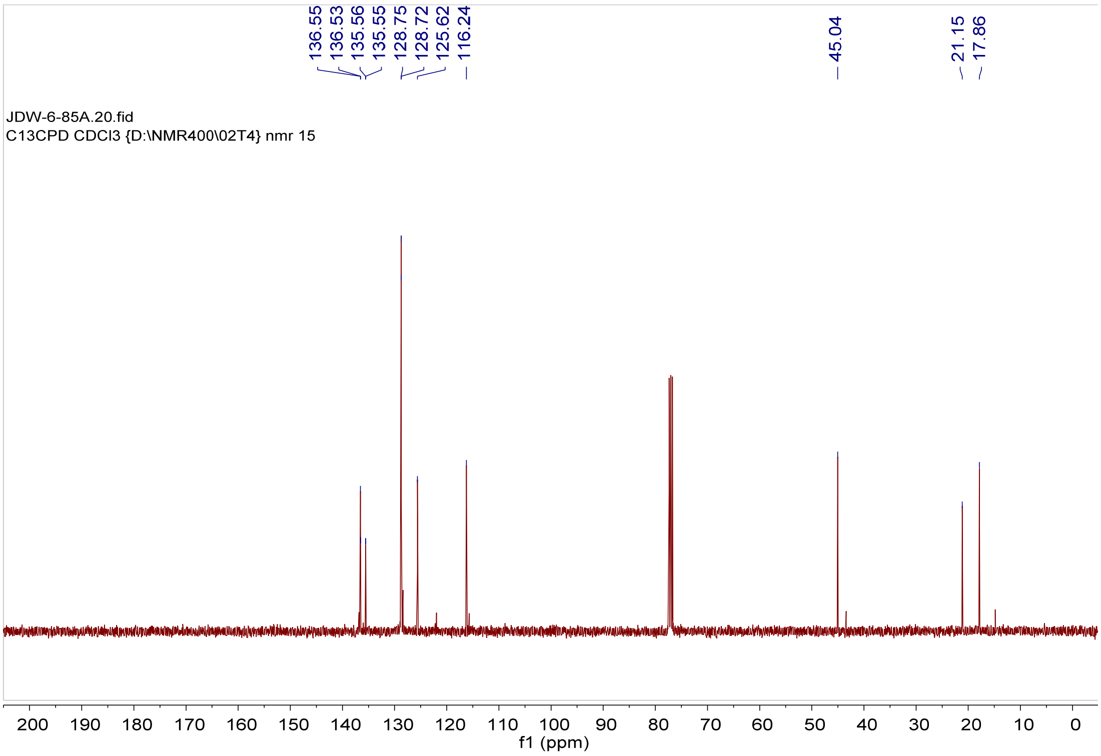

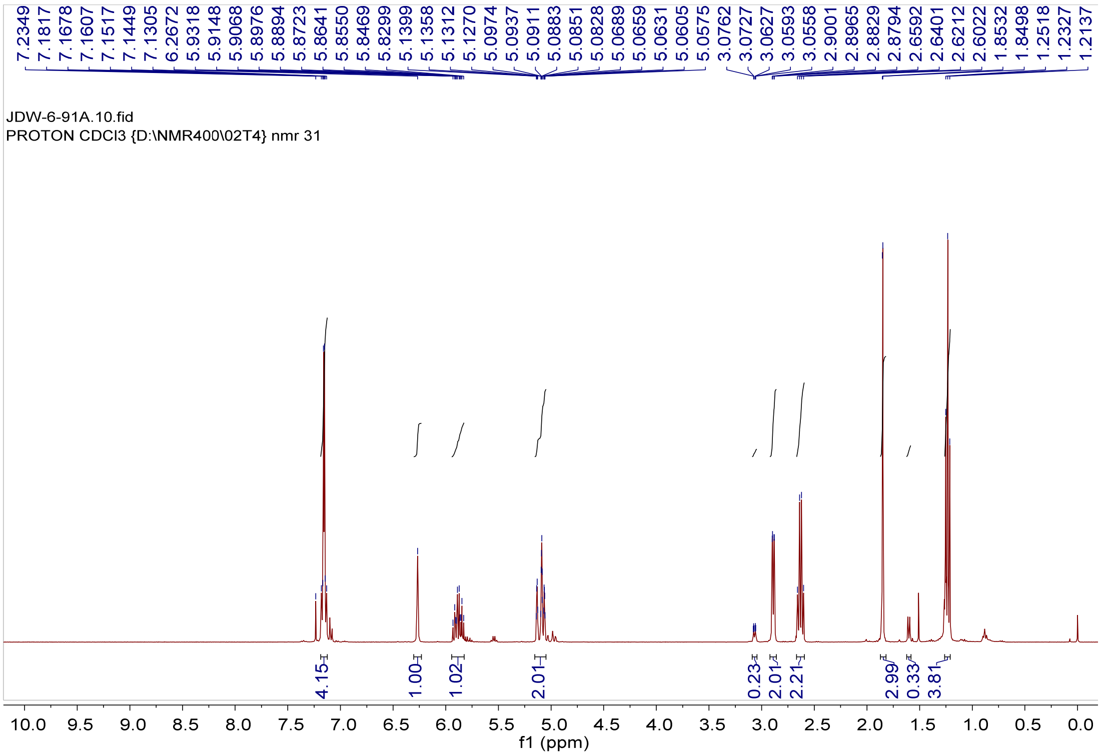

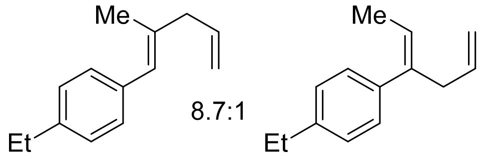

4c/4c' ^{1}H NMR (400 MHz, $CDCl_3$)
^{13}C NMR (100 MHz, $CDCl_3$)

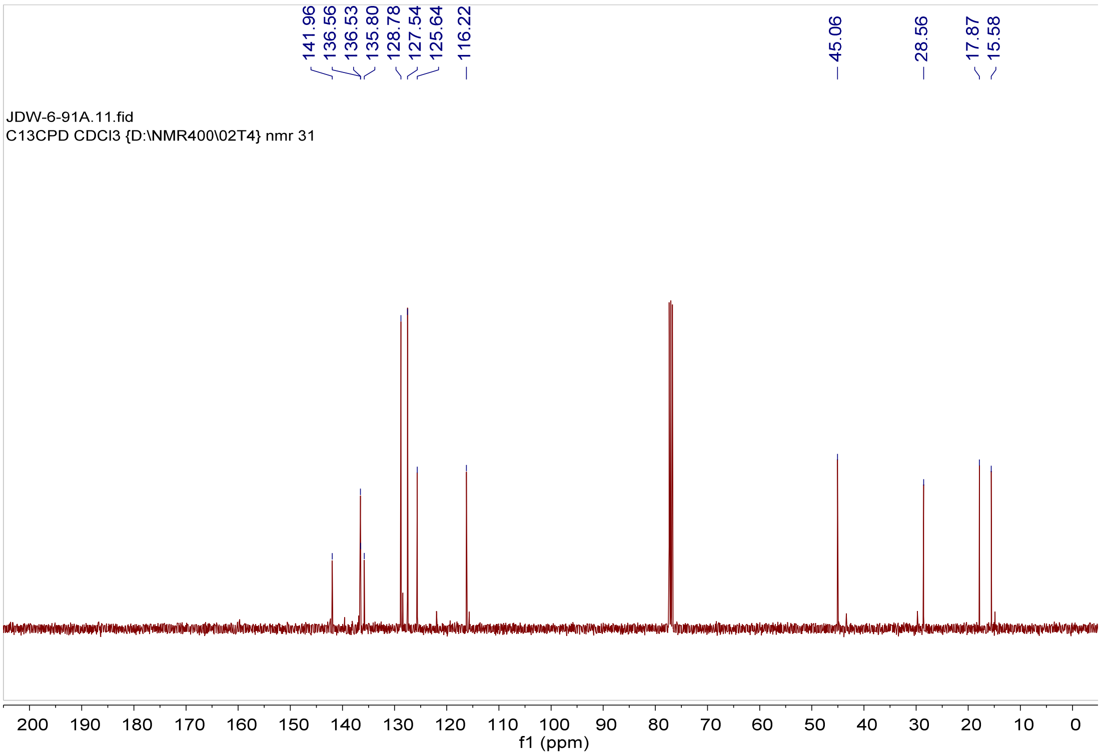

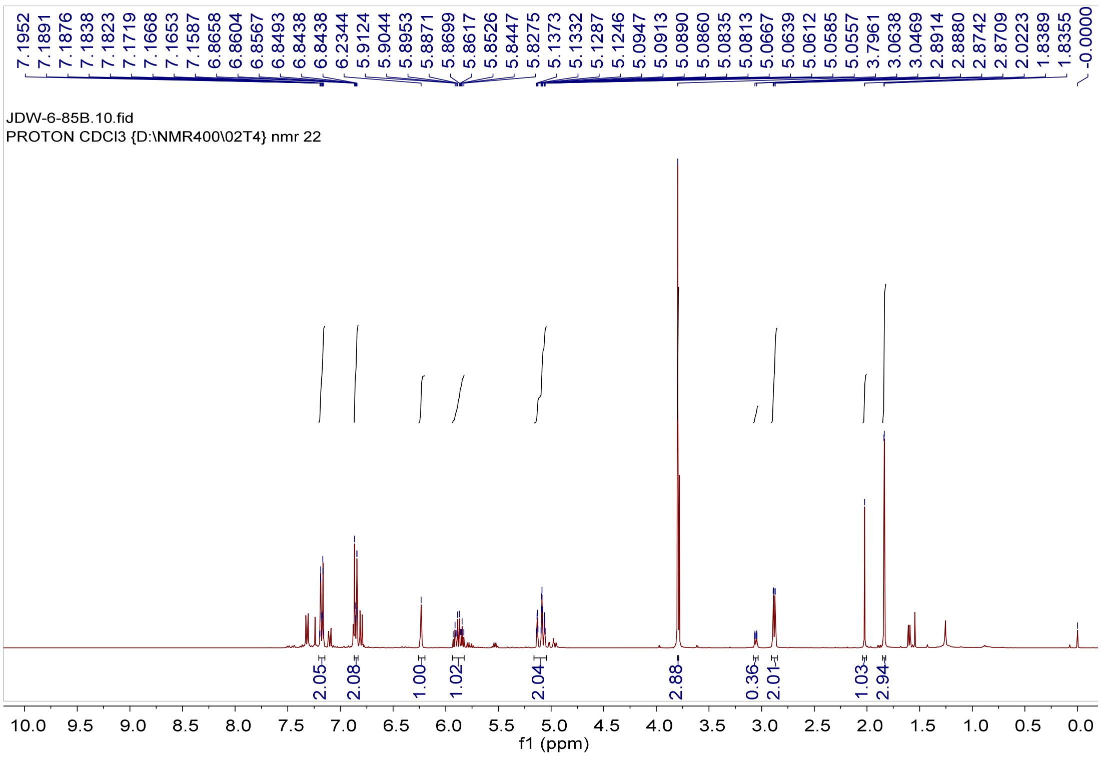

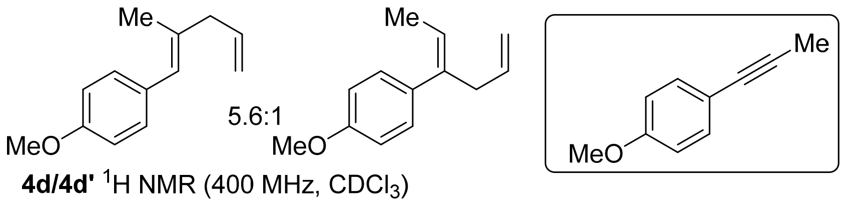

4d/4d' ^{1}H NMR (400 MHz, $CDCl_3$)

^{13}C NMR (100 MHz, $CDCl_3$)

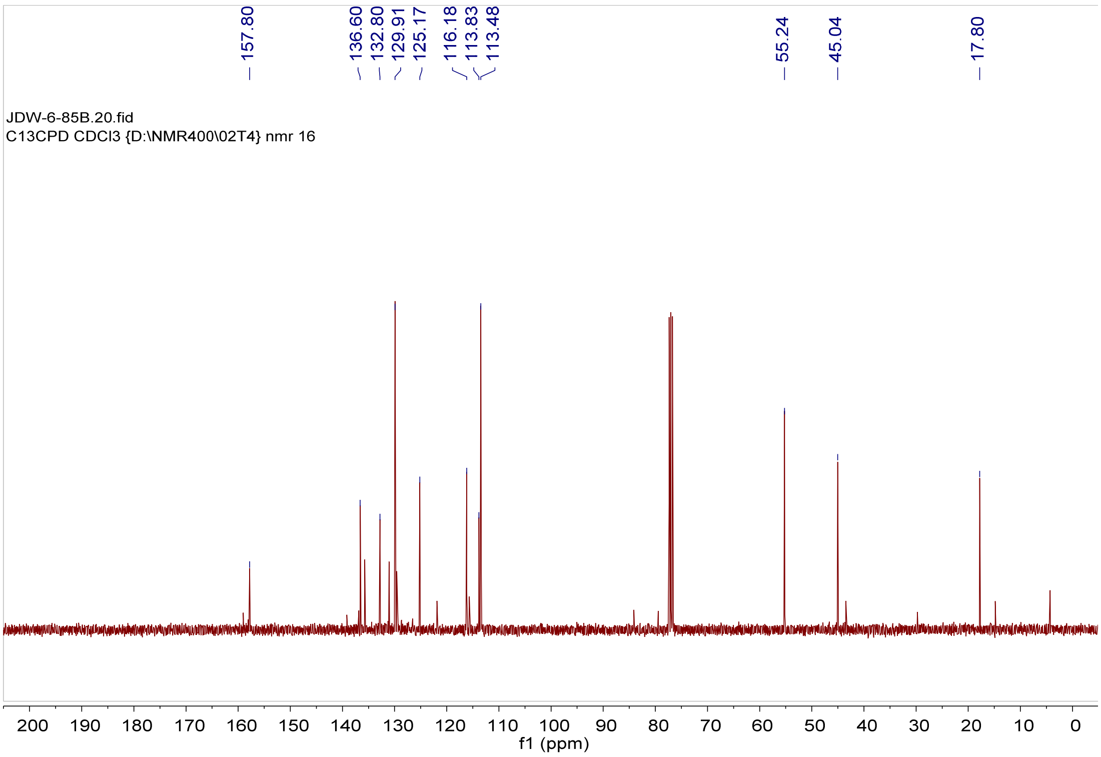

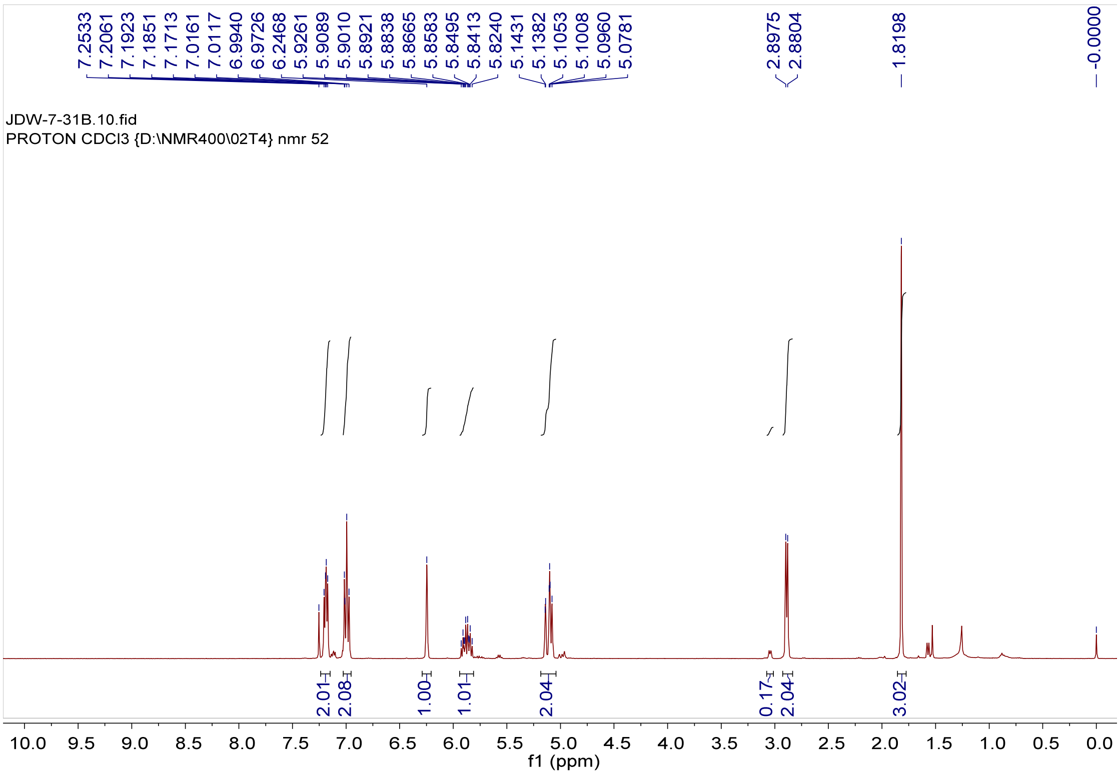

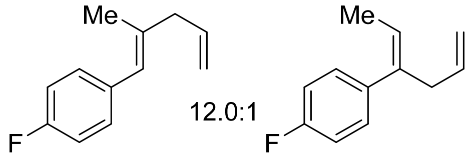

4e/4e' ^{1}H NMR (400 MHz, $CDCl_3$)

^{13}C NMR (100 MHz, $CDCl_3$)

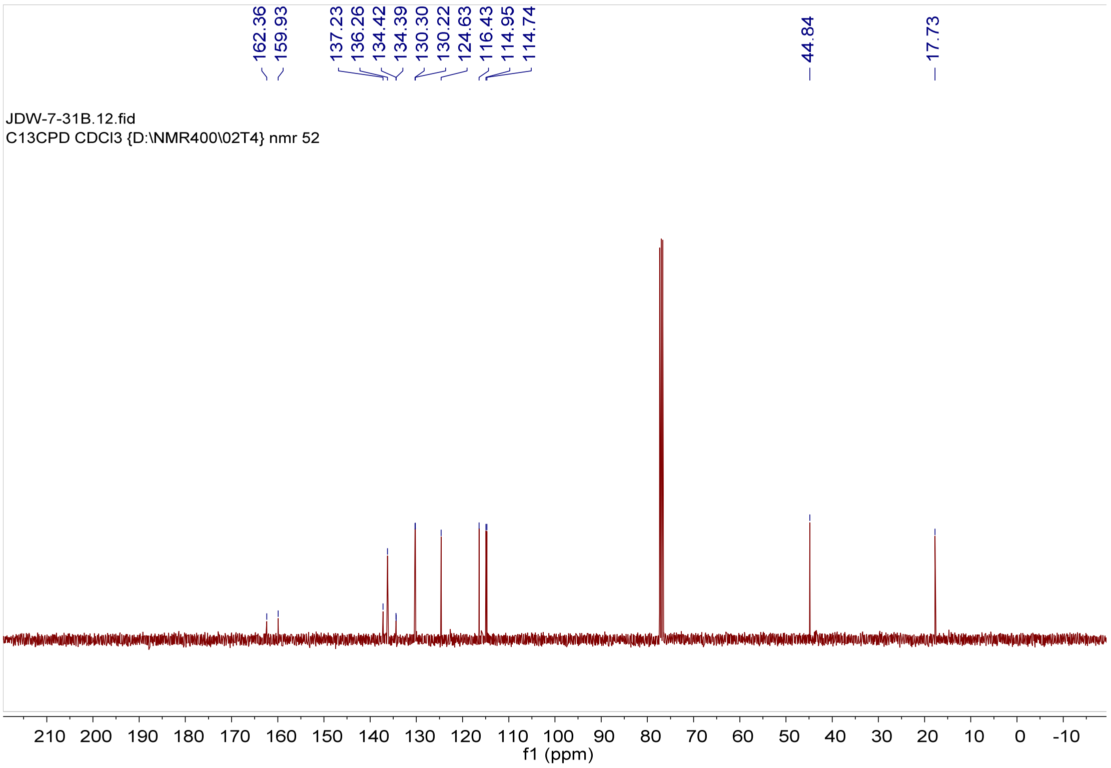

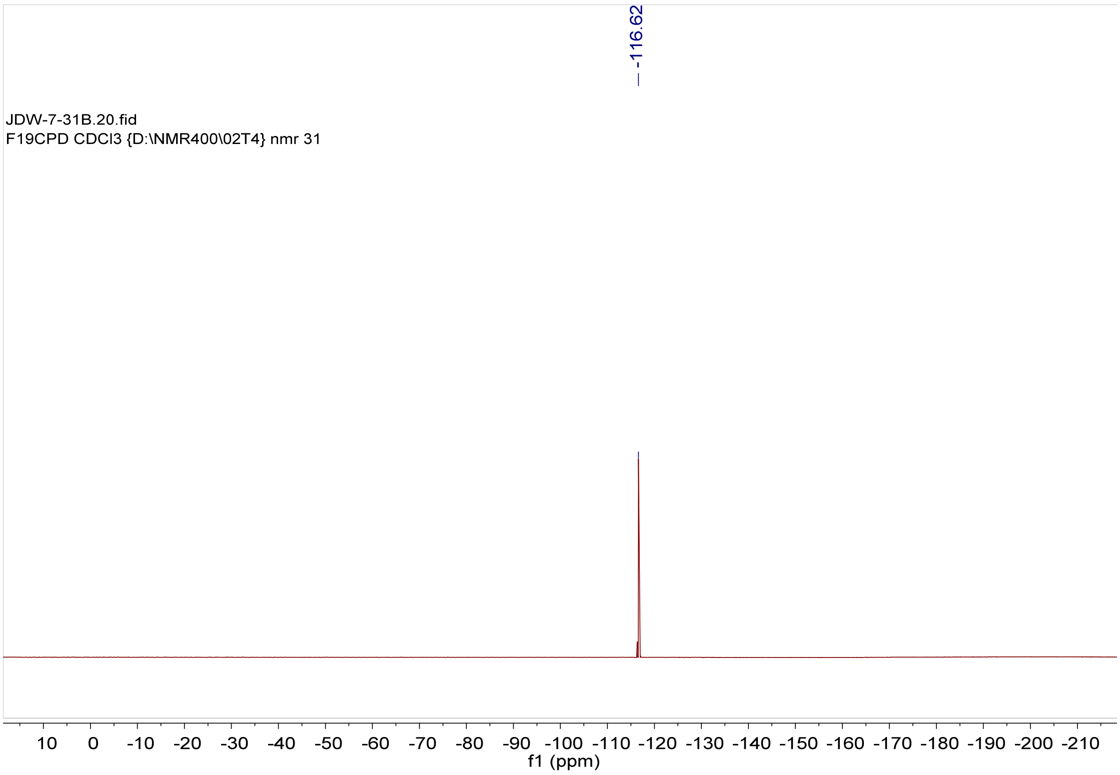

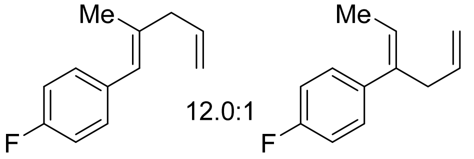

4e/4e' ^{19}F NMR (376 MHz, $CDCl_3$)

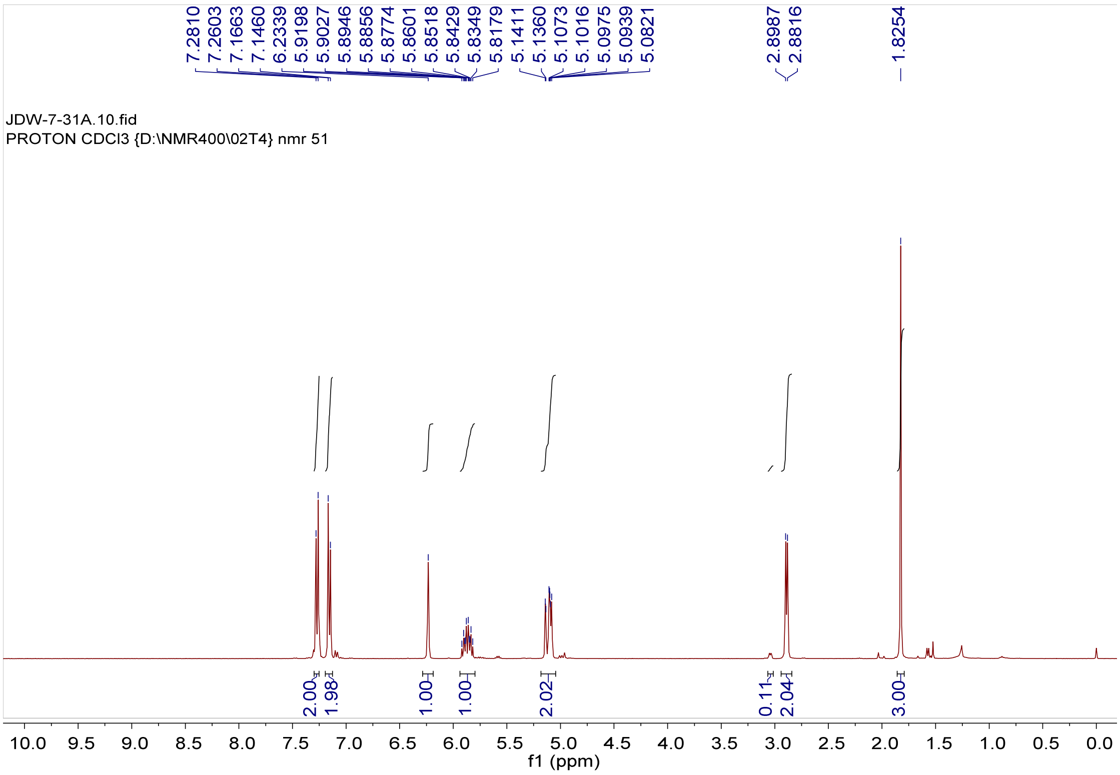

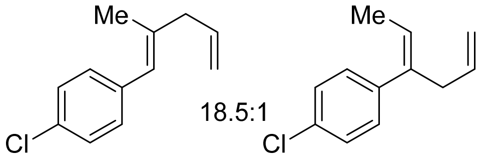

4f/4f' ^{1}H NMR (400 MHz, $CDCl_3$)

^{13}C NMR (100 MHz, $CDCl_3$)

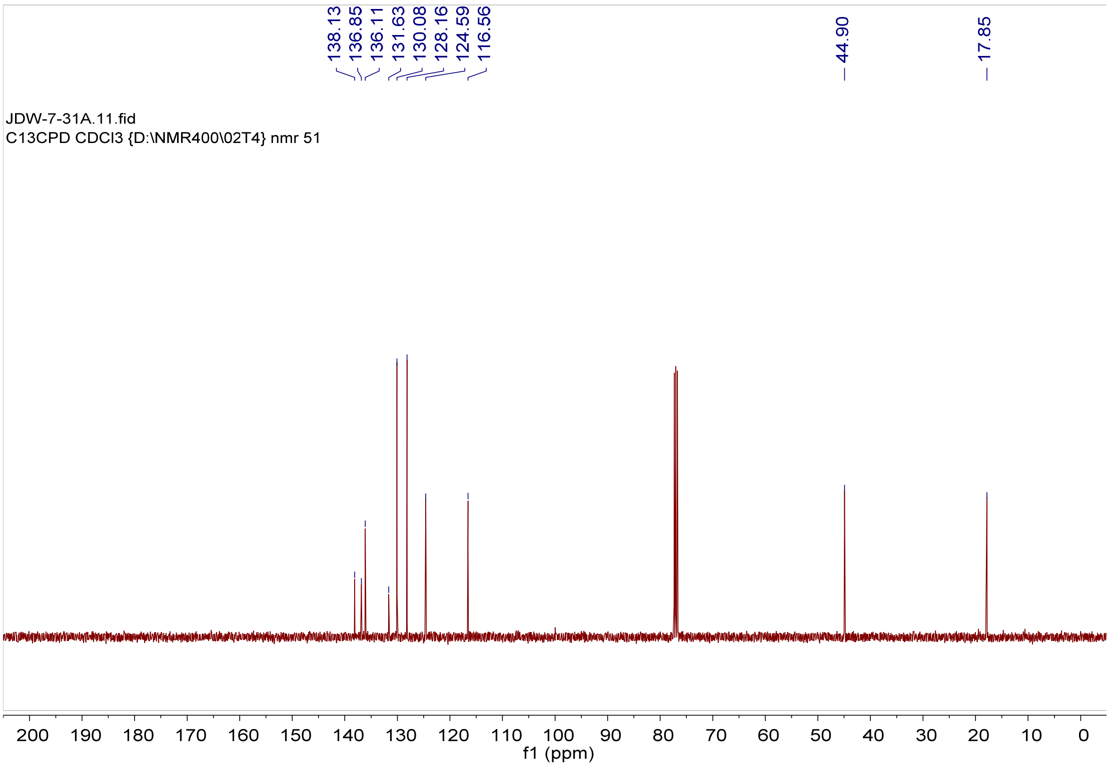

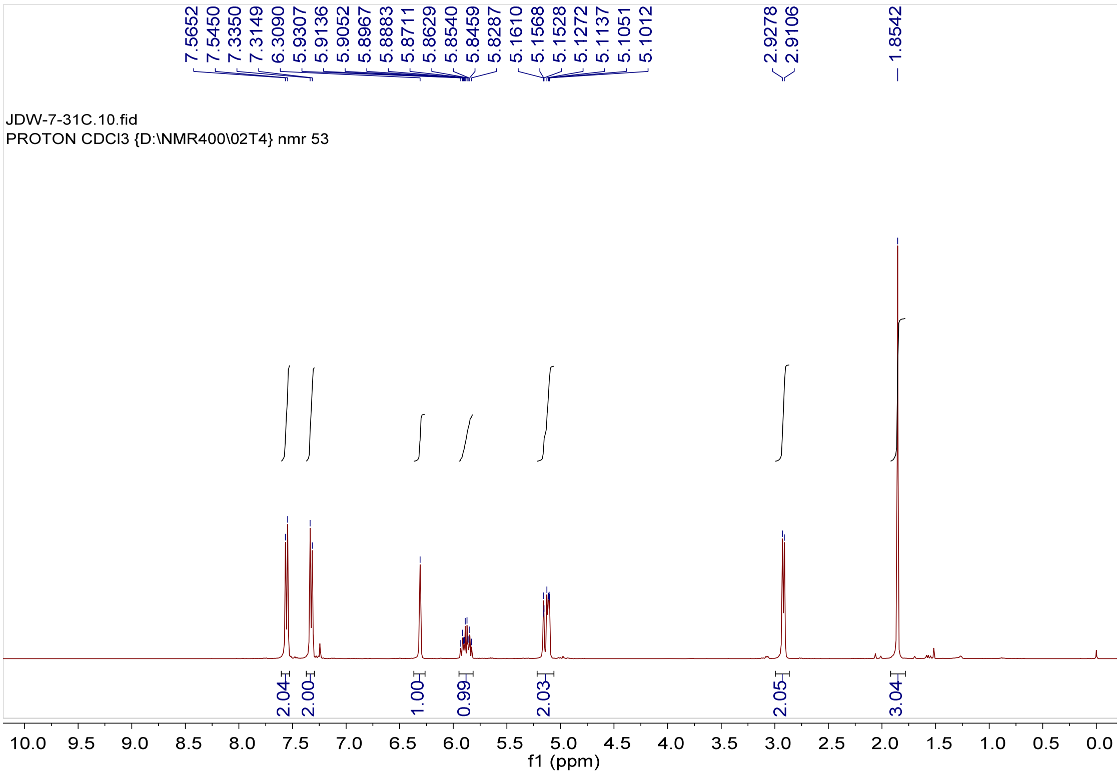

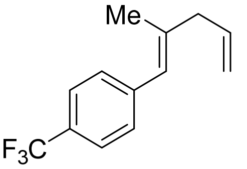

4g ^{1}H NMR (400 MHz, $CDCl_3$)

^{13}C NMR (100 MHz, $CDCl_3$)

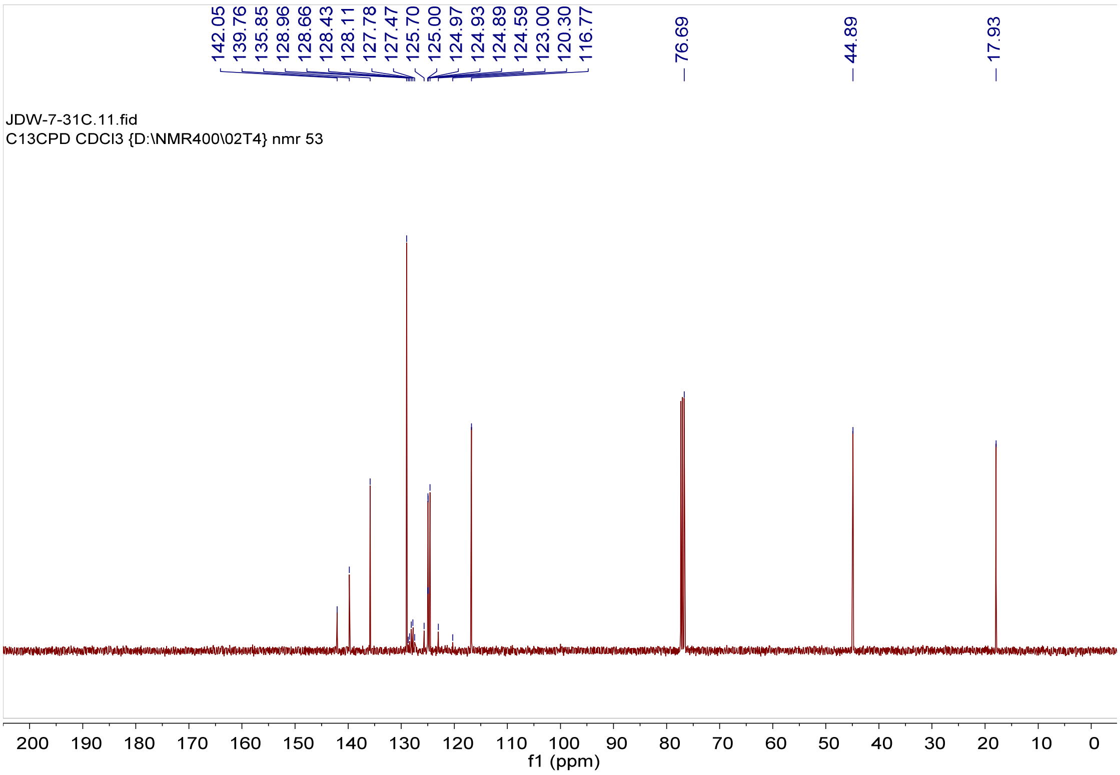

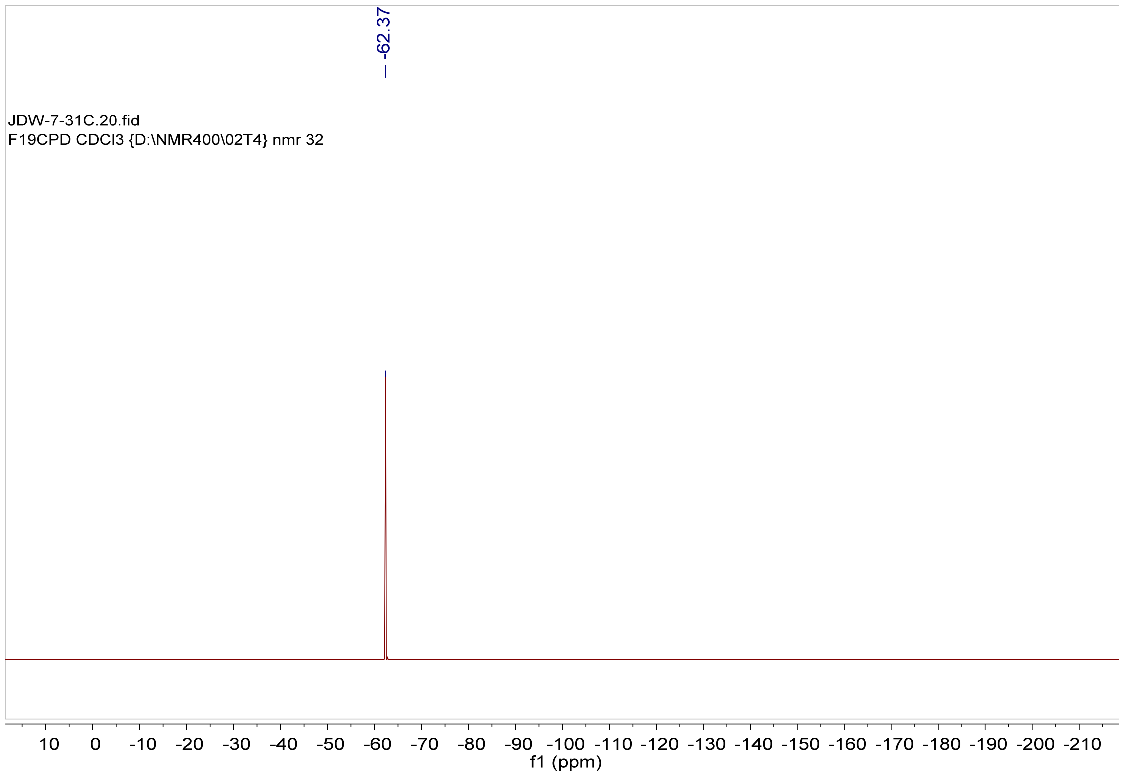

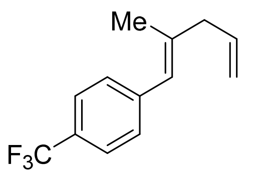

4g ^{19}F NMR (376 MHz, $CDCl_3$)

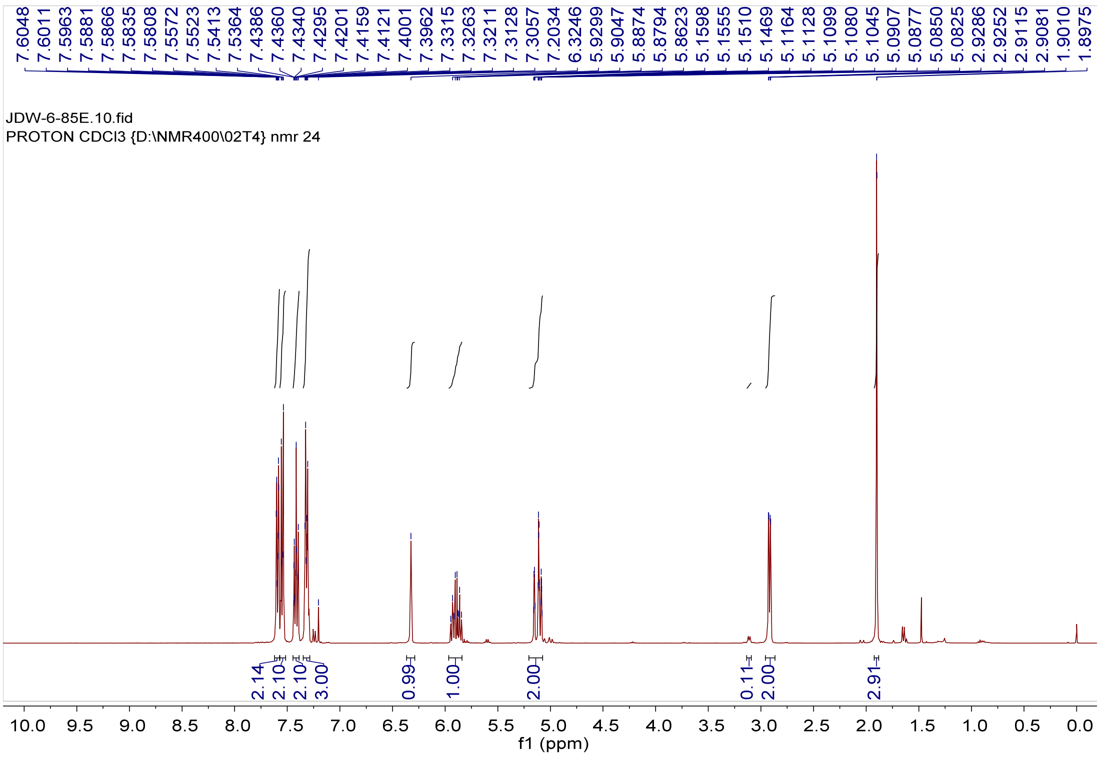

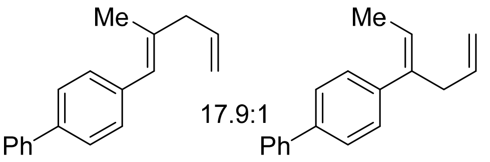

4h/4h' ^{1}H NMR (400 MHz, $CDCl_3$)
^{13}C NMR (100 MHz, $CDCl_3$)

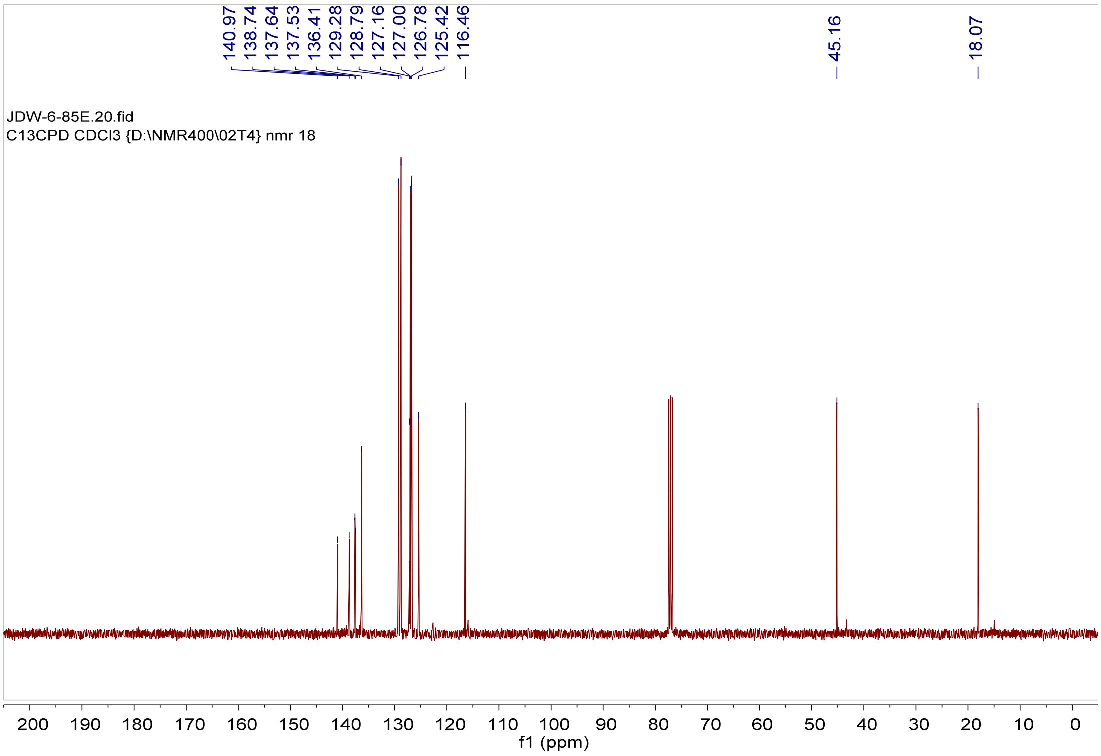

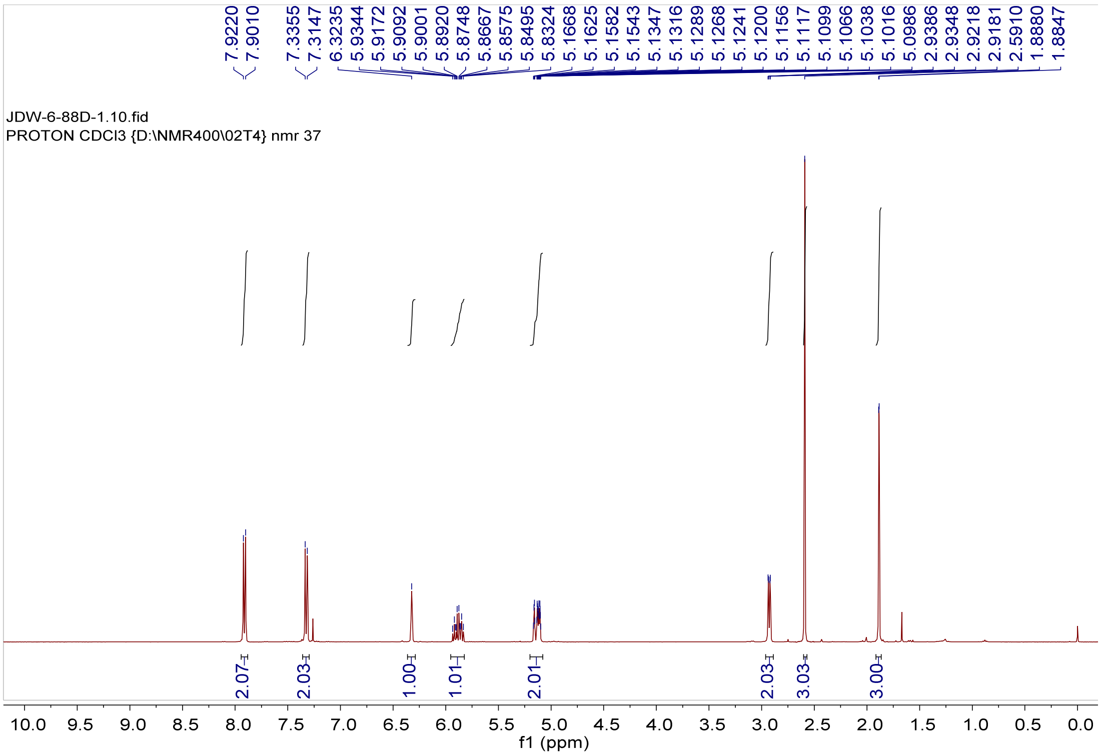

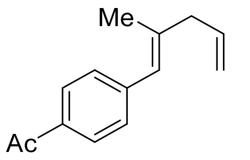

4i ^{1}H NMR (400 MHz, $CDCl_3$)
^{13}C NMR (100 MHz, $CDCl_3$)

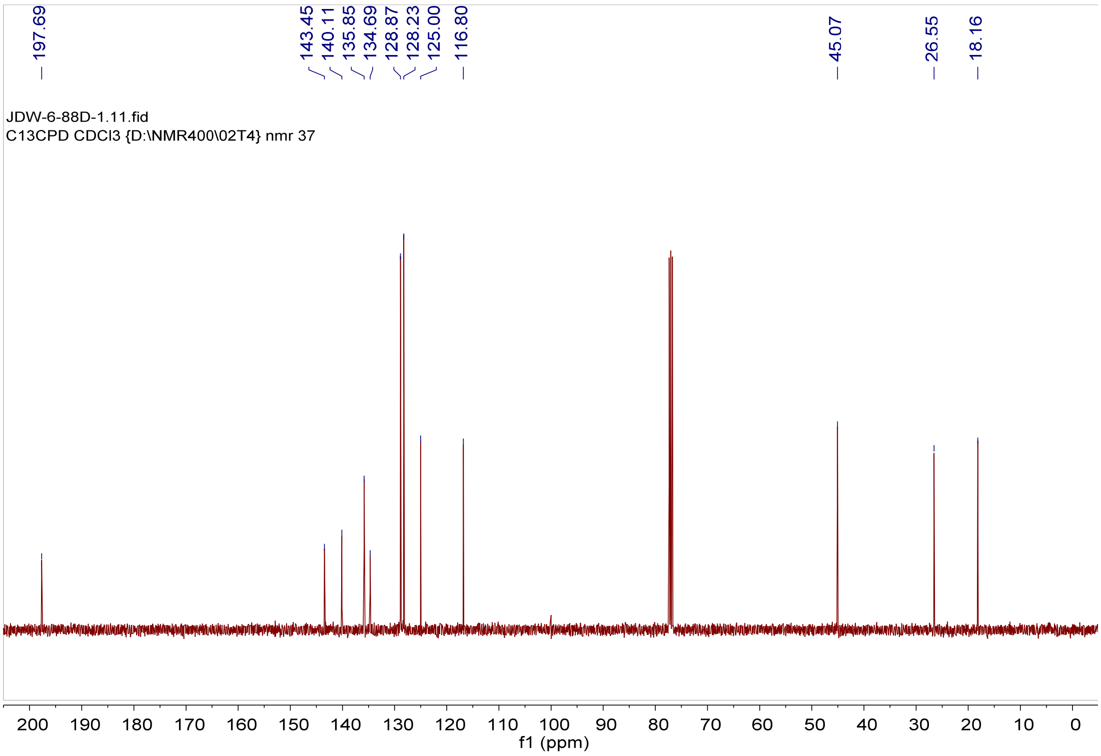

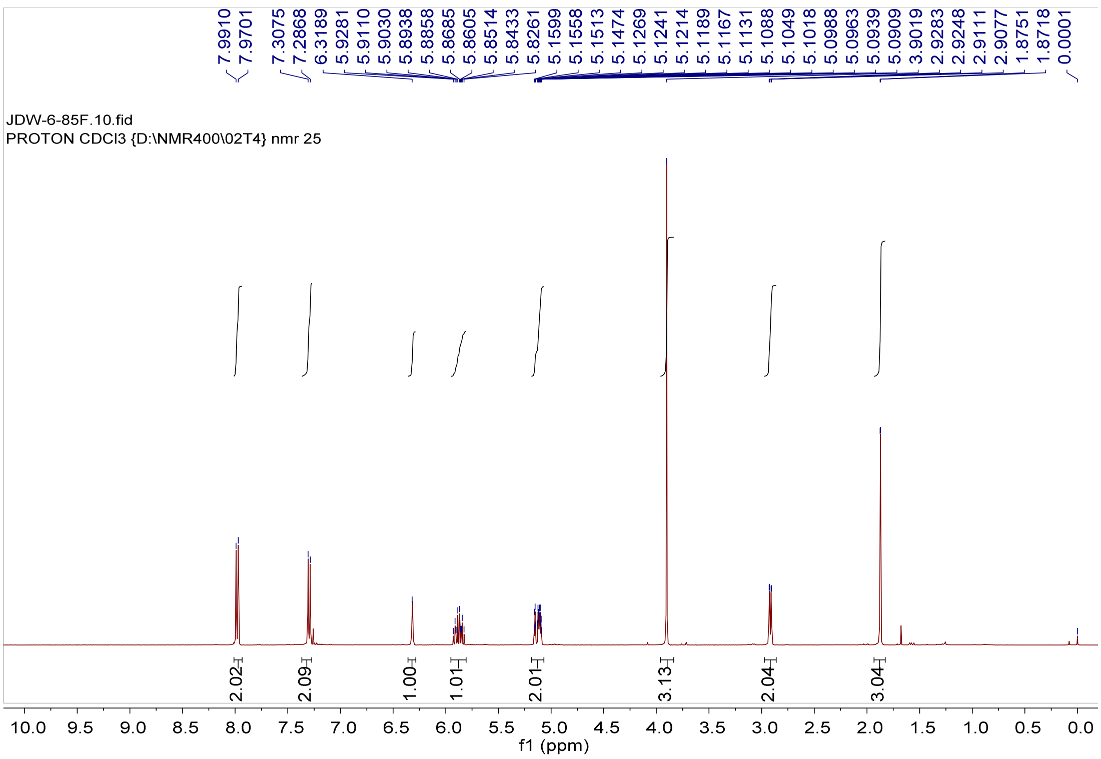

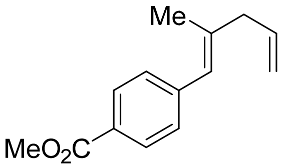

4j ^{1}H NMR (400 MHz, $CDCl_3$)

^{13}C NMR (100 MHz, $CDCl_3$)

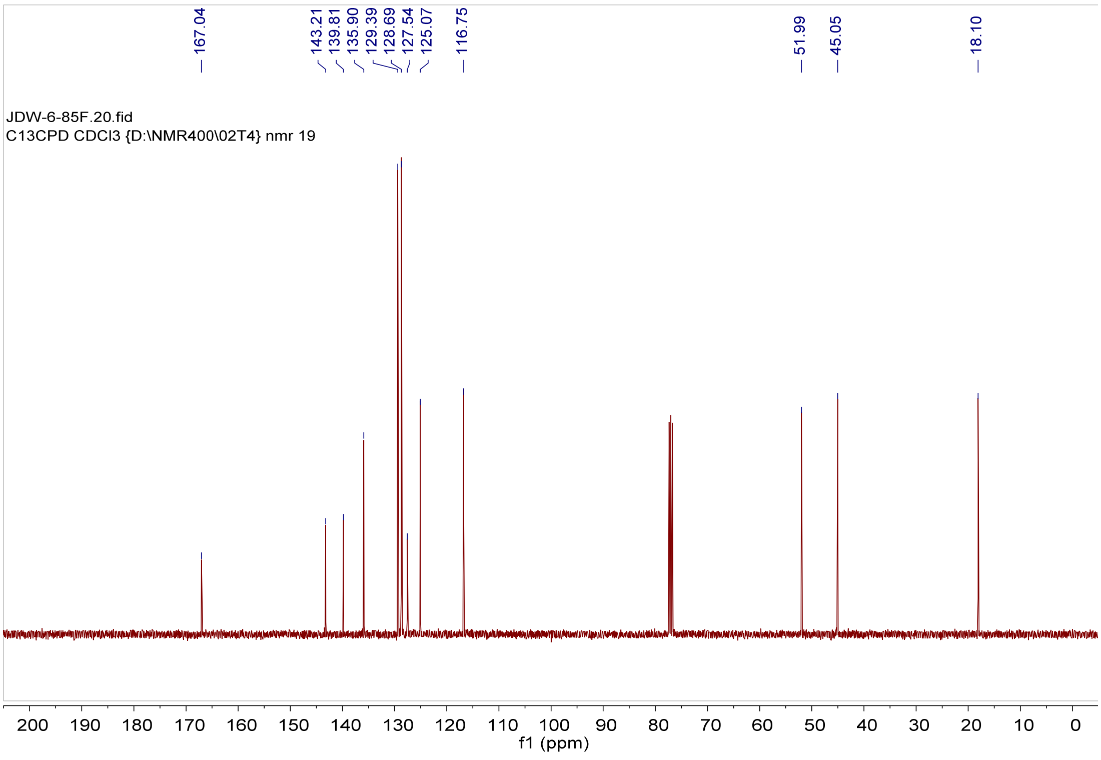

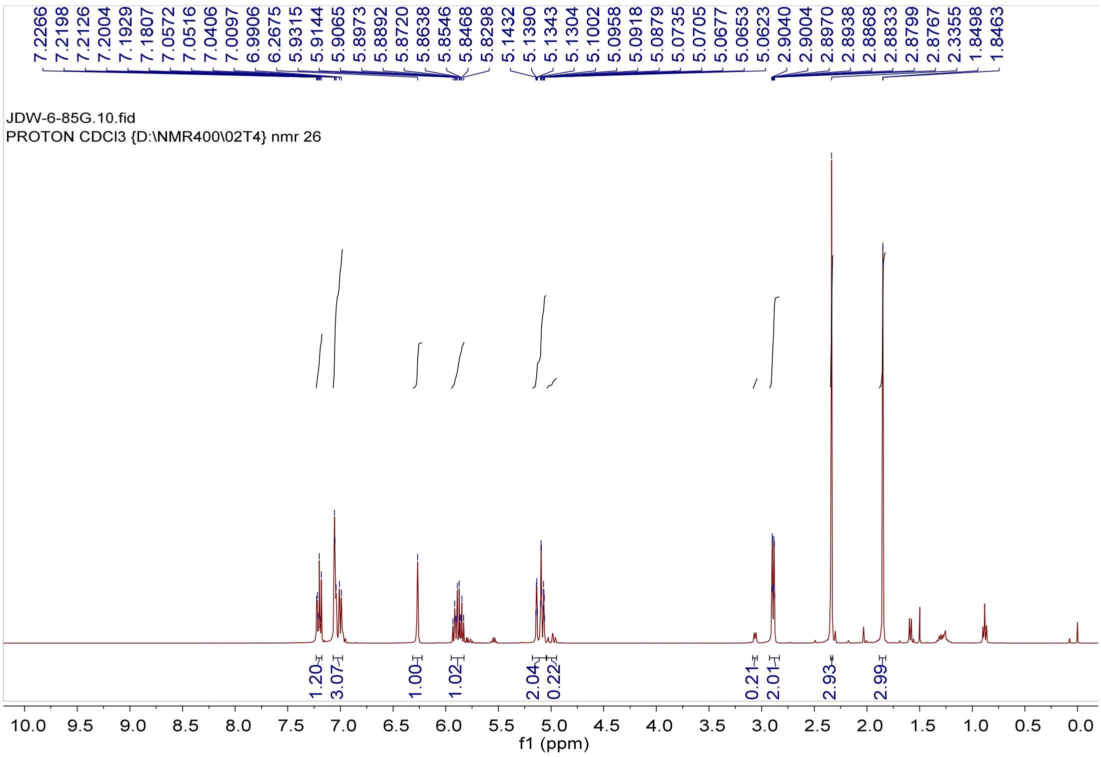

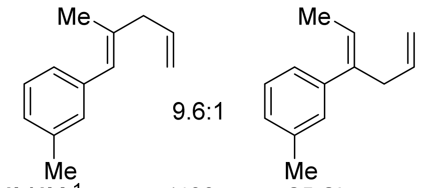

4k/4k' ^{1}H NMR (400 MHz, $CDCl_3$)

^{13}C NMR (100 MHz, $CDCl_3$)

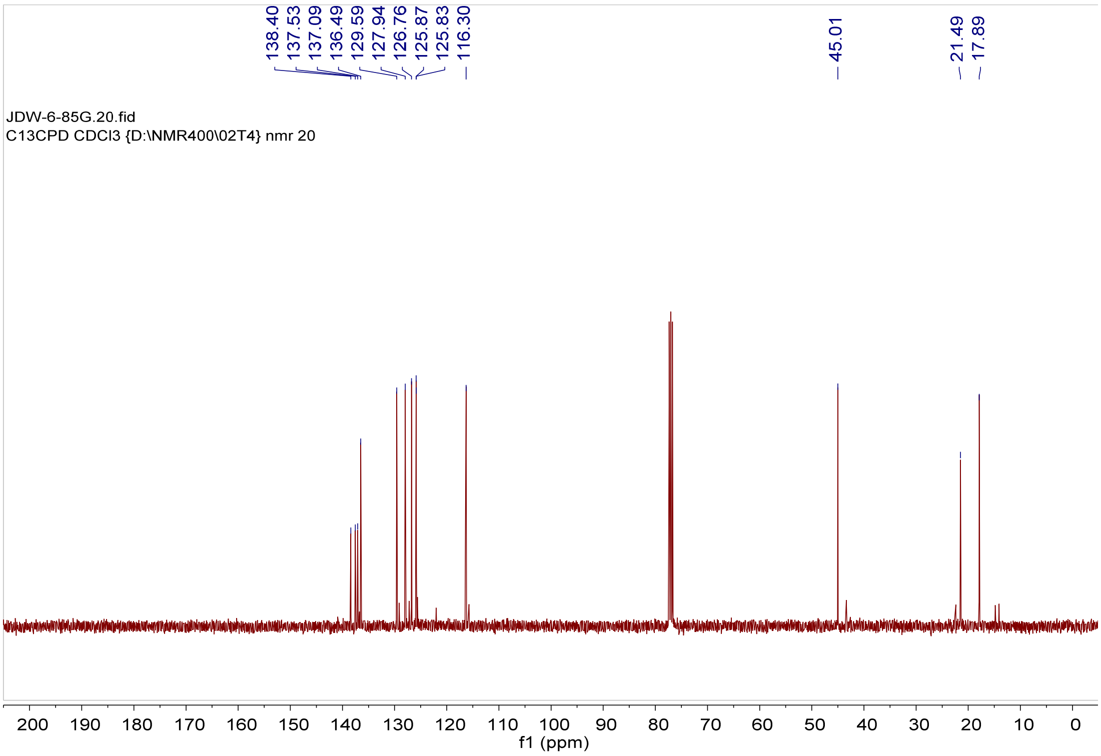

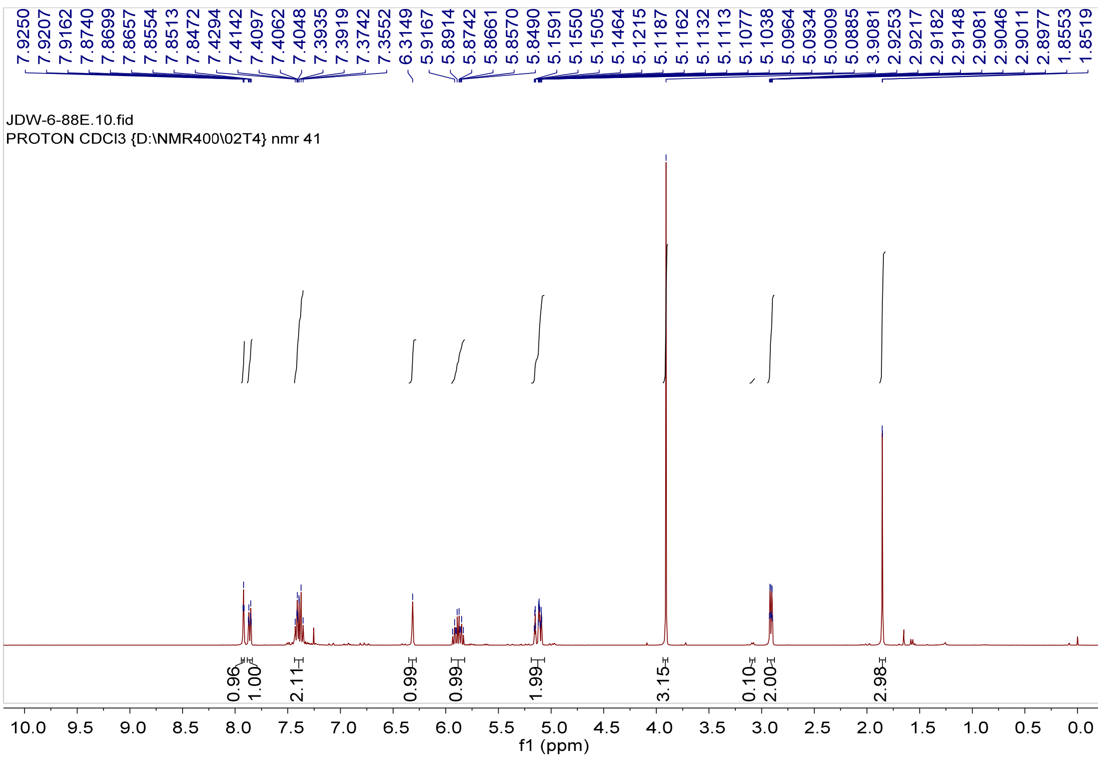

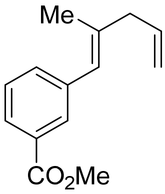

4l ^{1}H NMR (400 MHz, $CDCl_3$)

^{13}C NMR (100 MHz, $CDCl_3$)

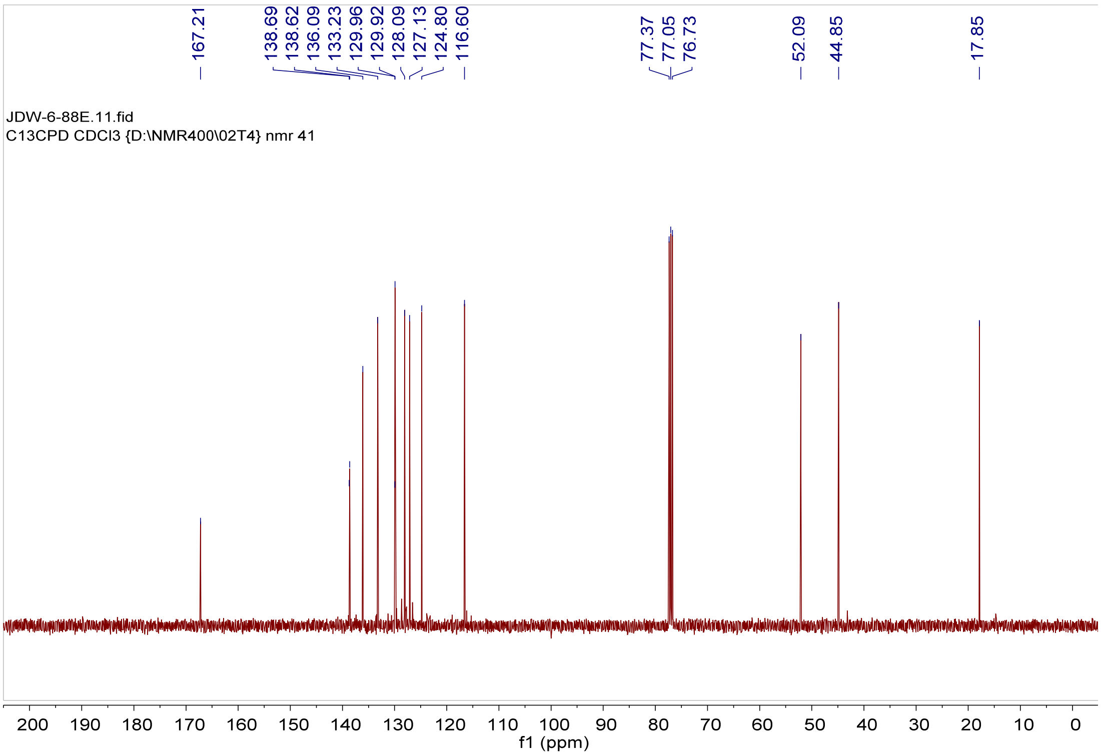

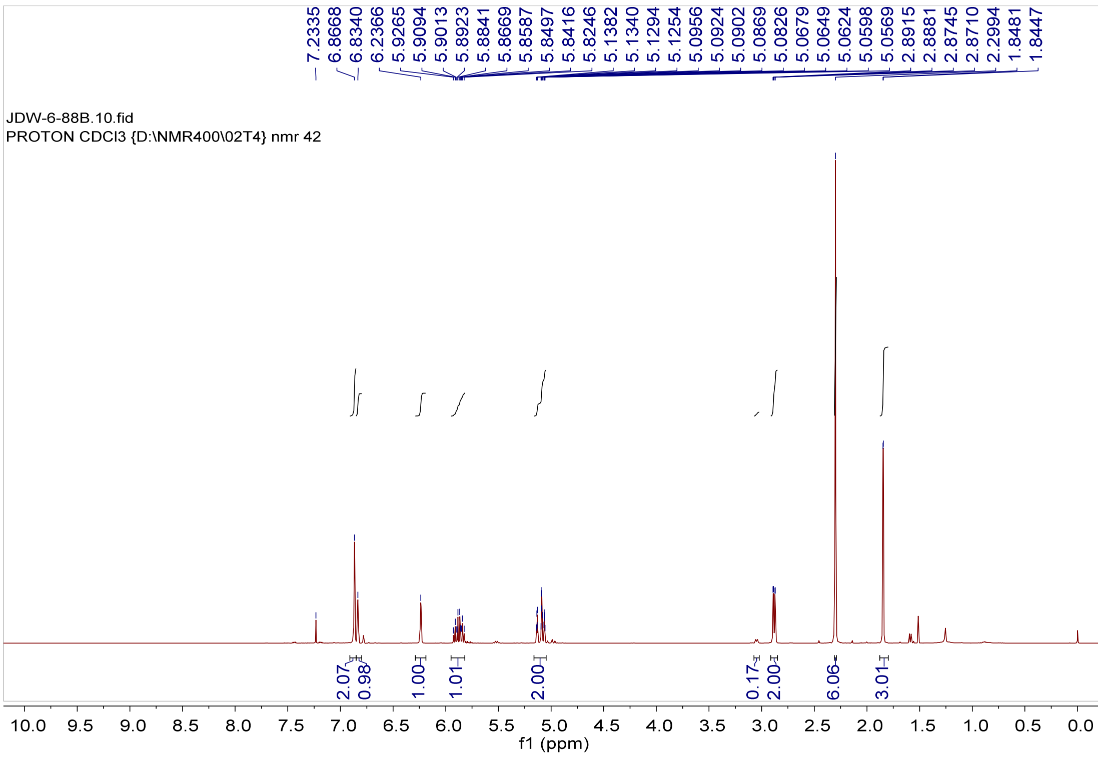

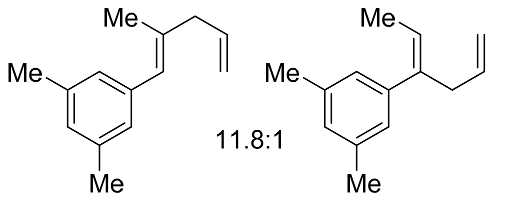

4m/4m' ^{1}H NMR (400 MHz, $CDCl_3$)
^{13}C NMR (100 MHz, $CDCl_3$)

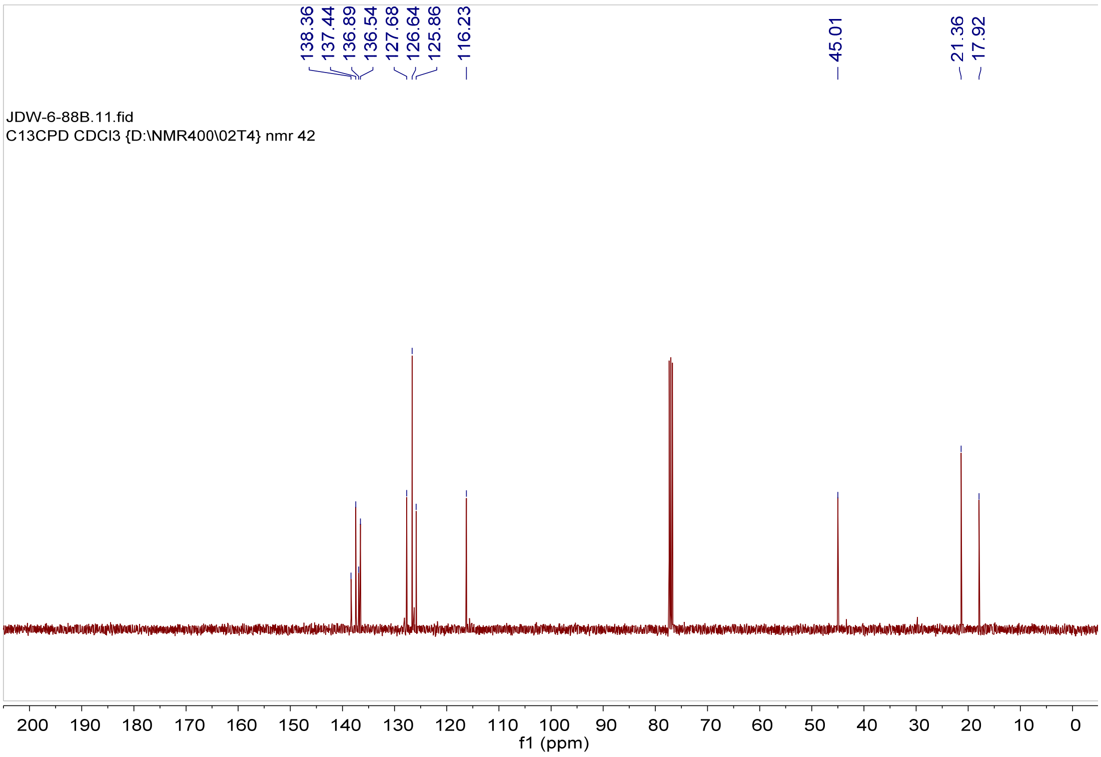

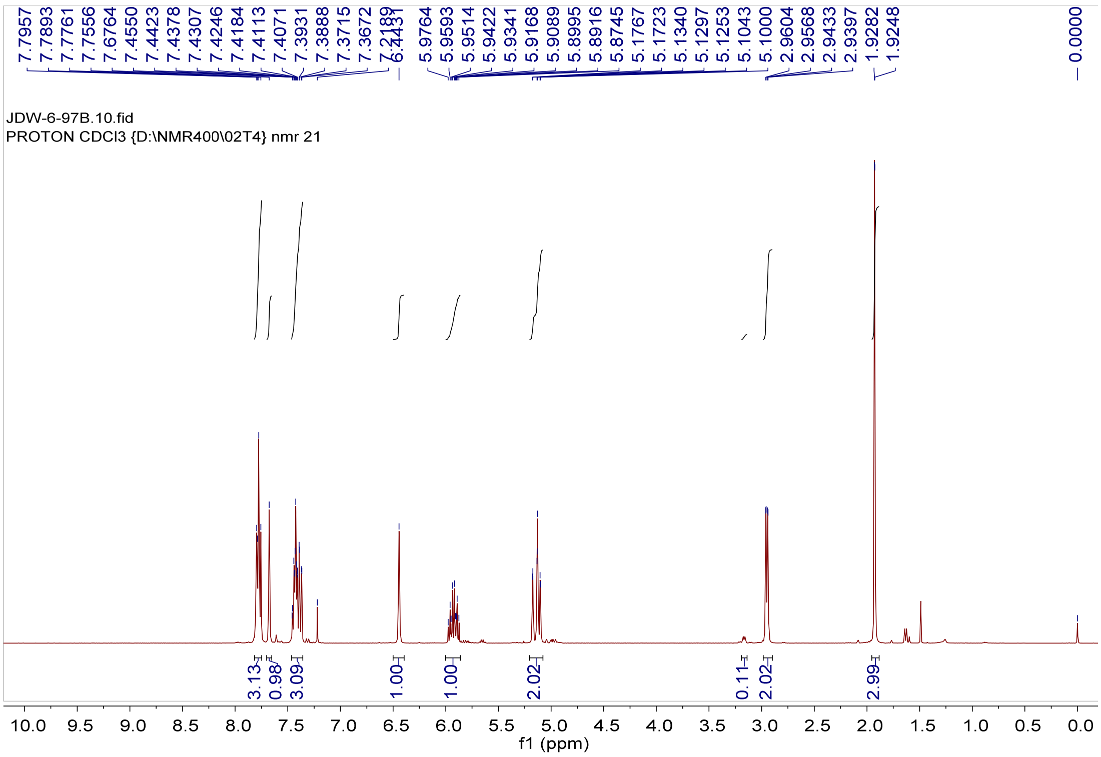

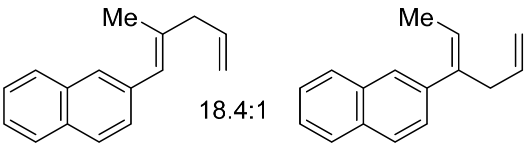

4o/4o' ^{1}H NMR (400 MHz, $CDCl_3$)
^{13}C NMR (100 MHz, $CDCl_3$)

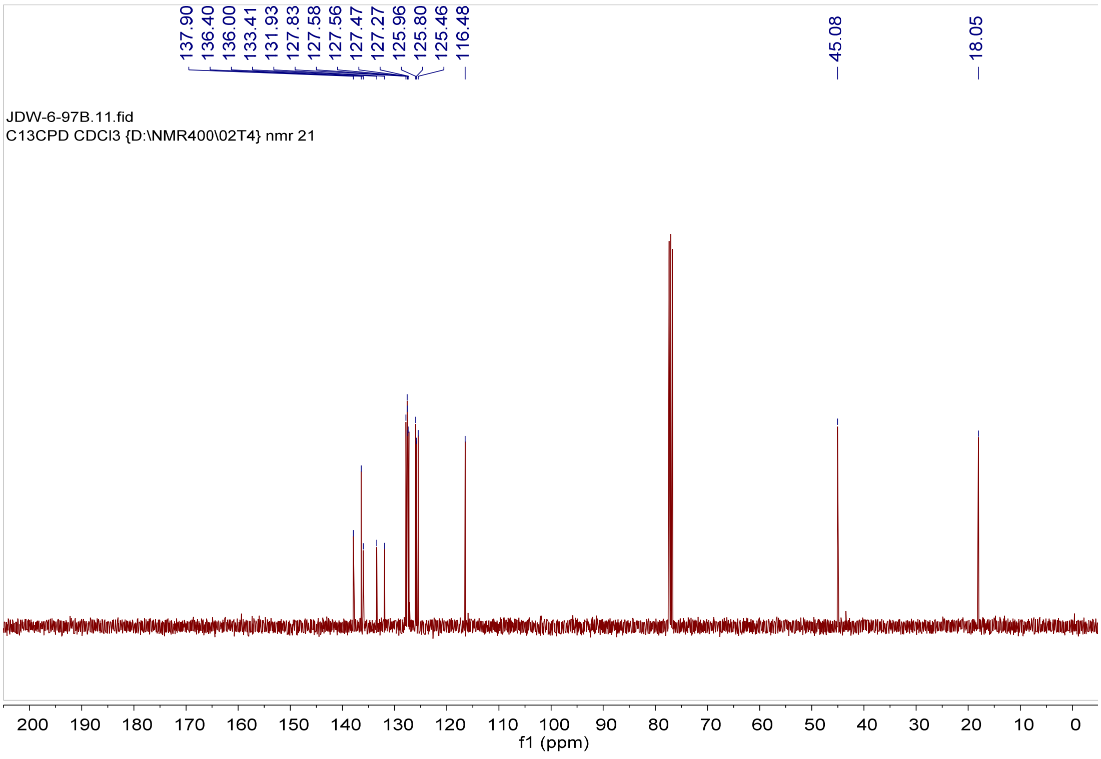

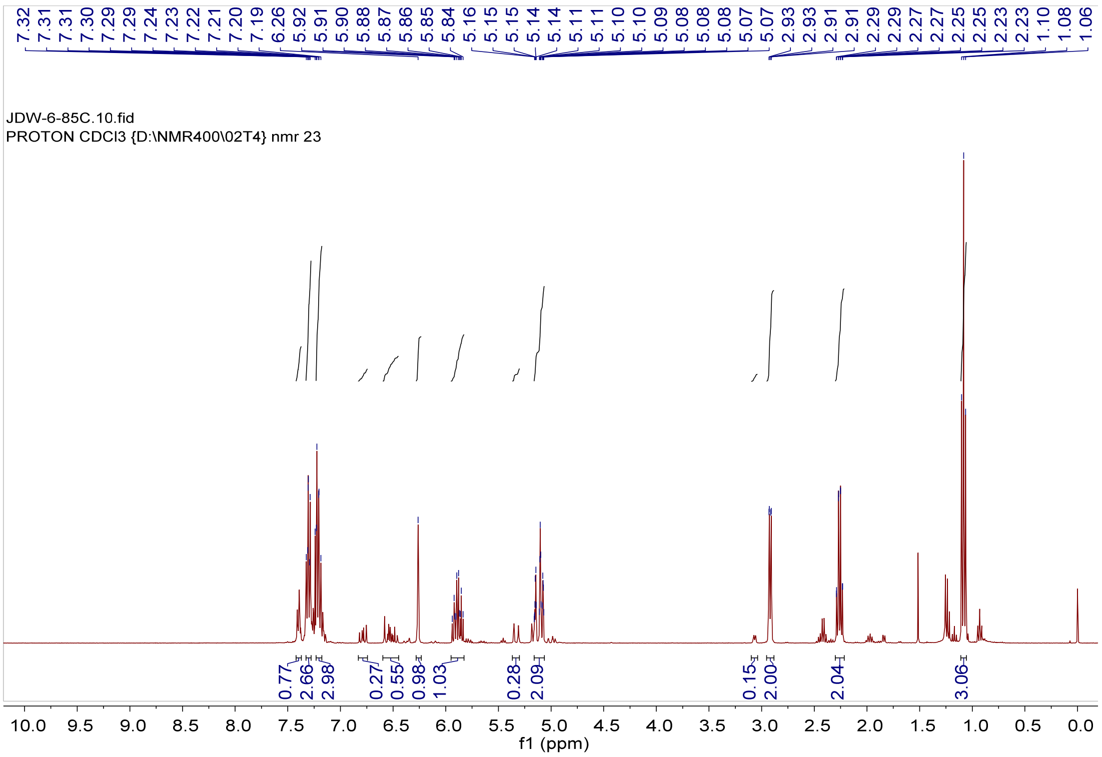

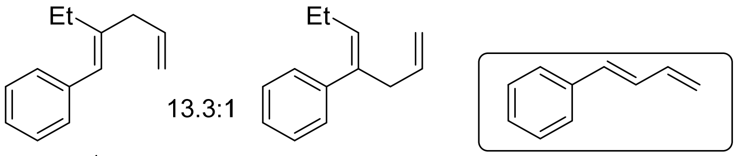

4p/4p' ^{1}H NMR (400 MHz, $CDCl_3$)
^{13}C NMR (100 MHz, $CDCl_3$)

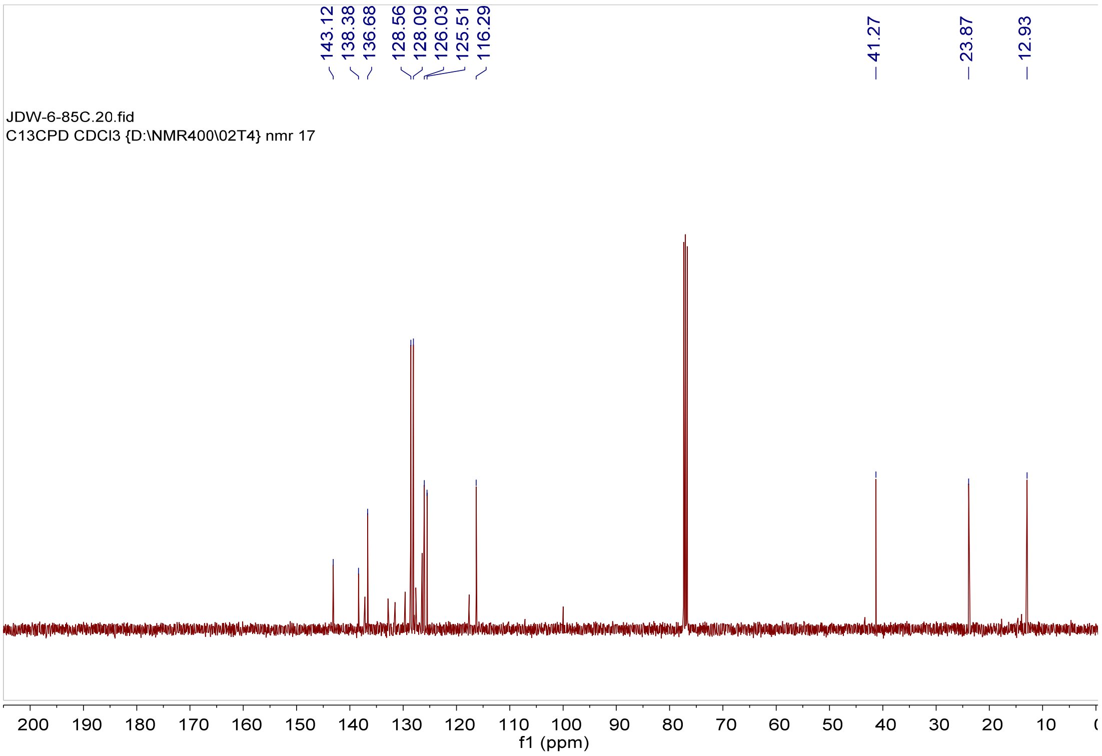

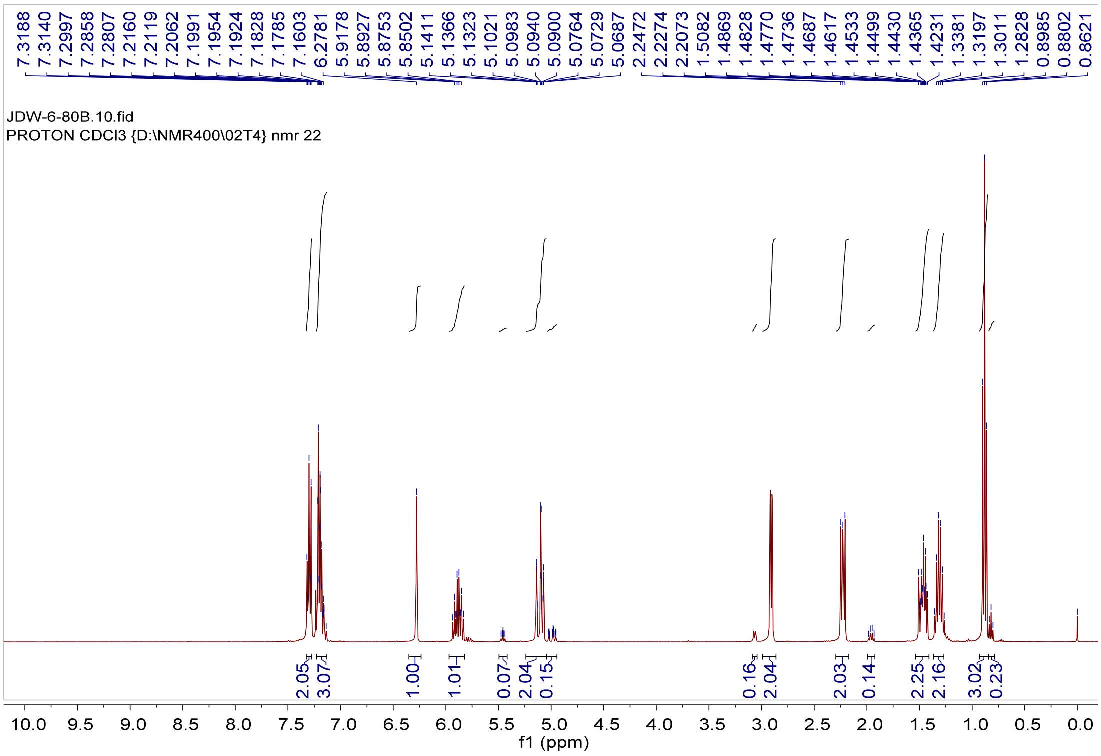

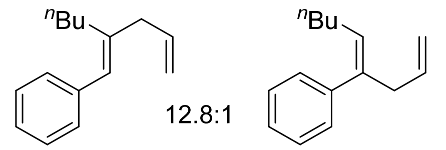

4q/4q' ^{1}H NMR (400 MHz, $CDCl_3$)
^{13}C NMR (100 MHz, $CDCl_3$)

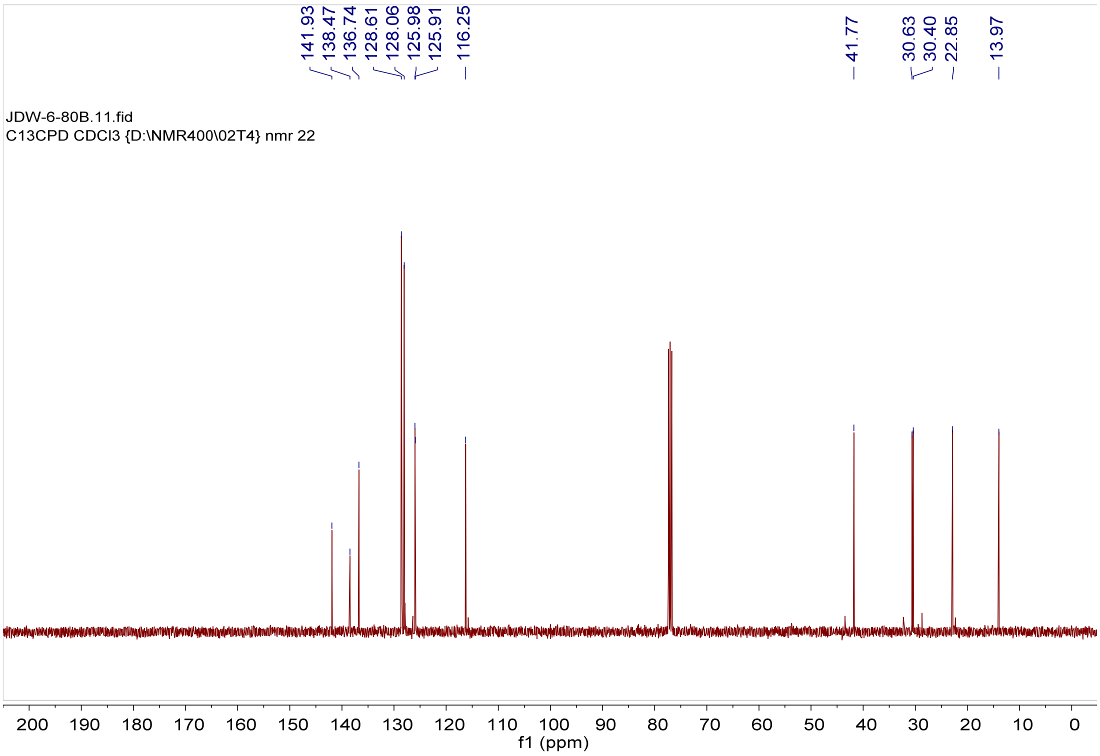

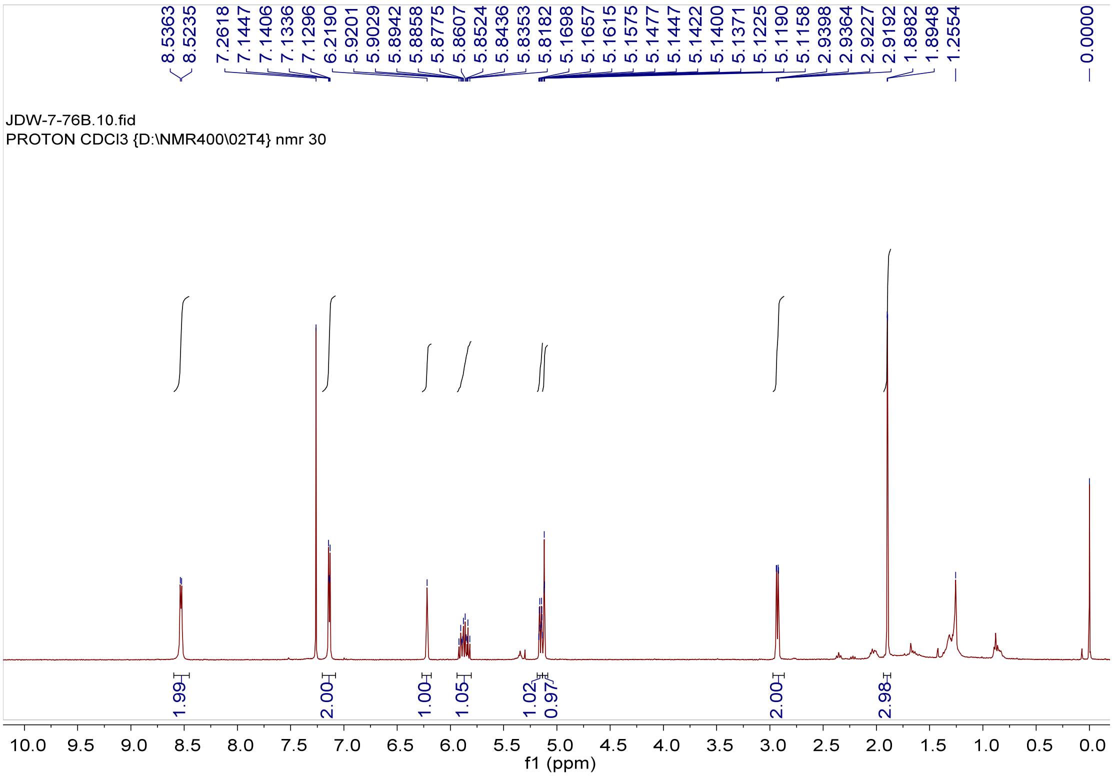

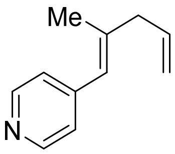

4r ^{1}H NMR (400 MHz, $CDCl_3$)

^{13}C NMR (100 MHz, $CDCl_3$)

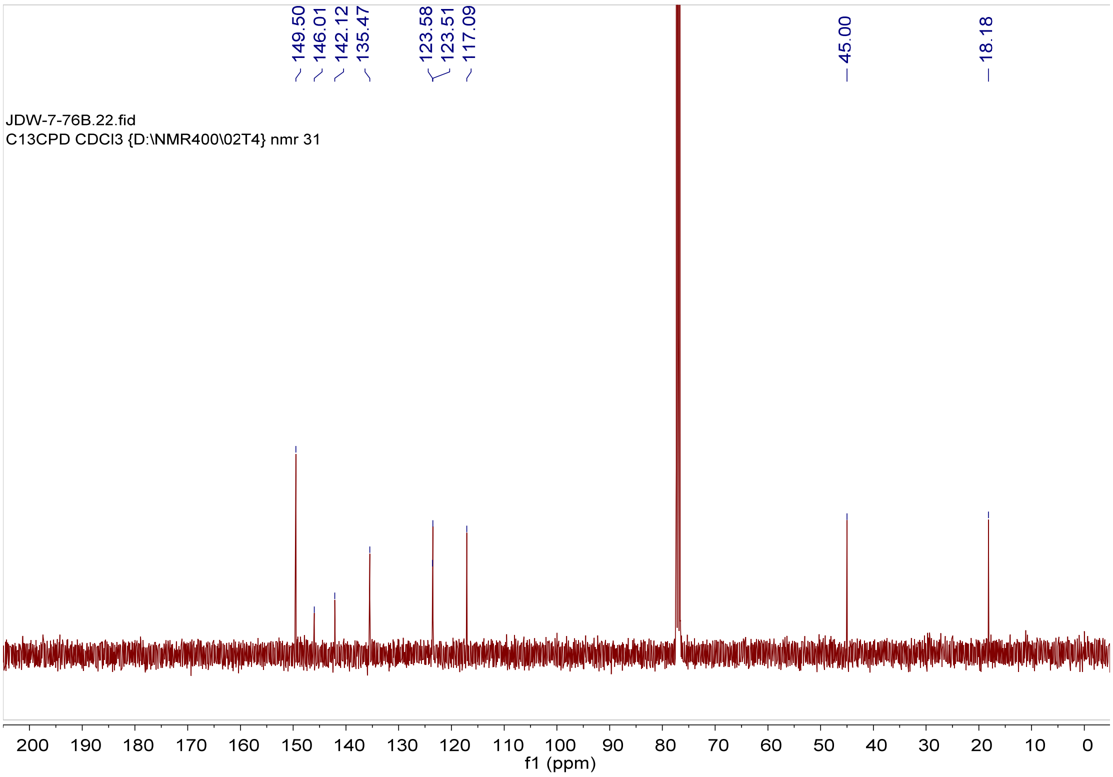

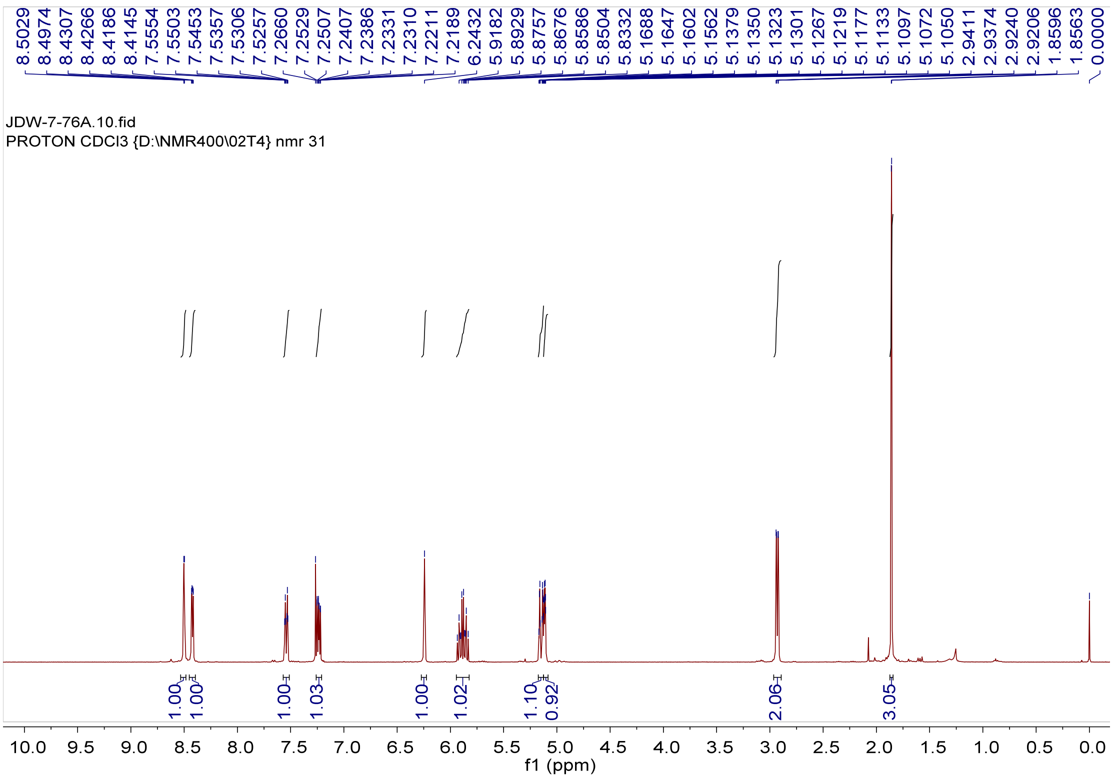

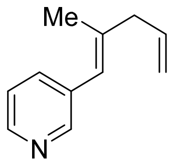

4s ^{1}H NMR (400 MHz, $CDCl_3$)

^{13}C NMR (100 MHz, $CDCl_3$)

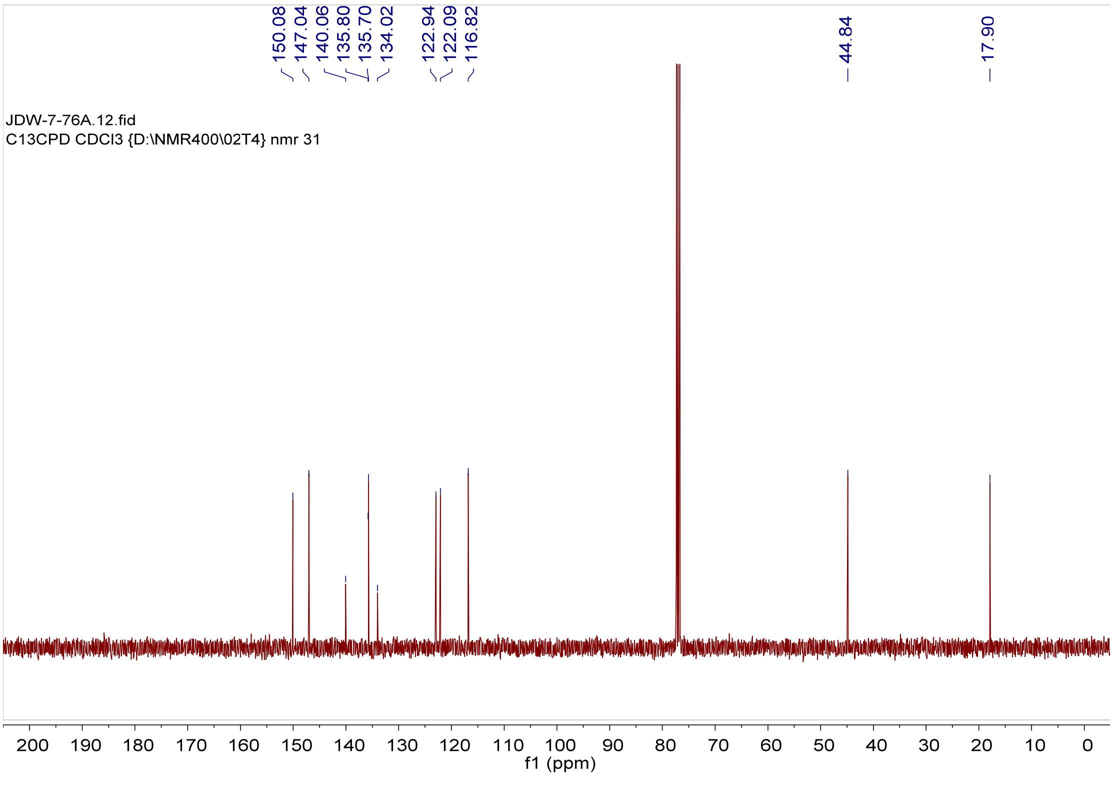

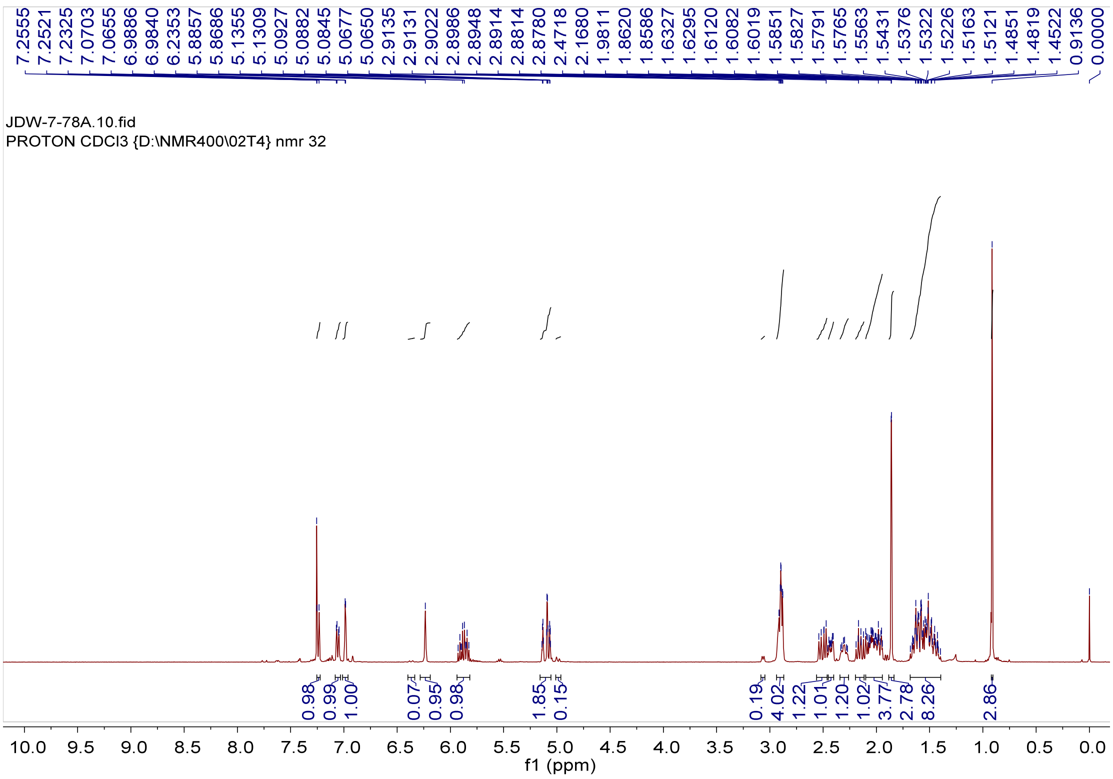

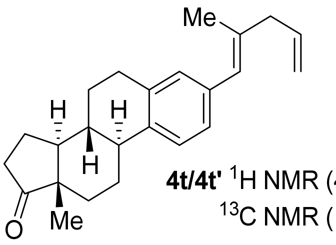

13.6:1

4t/4t' ^{1}H NMR (400 MHz, $CDCl_3$)

^{13}C NMR (100 MHz, $CDCl_3$)

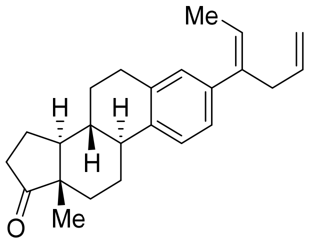

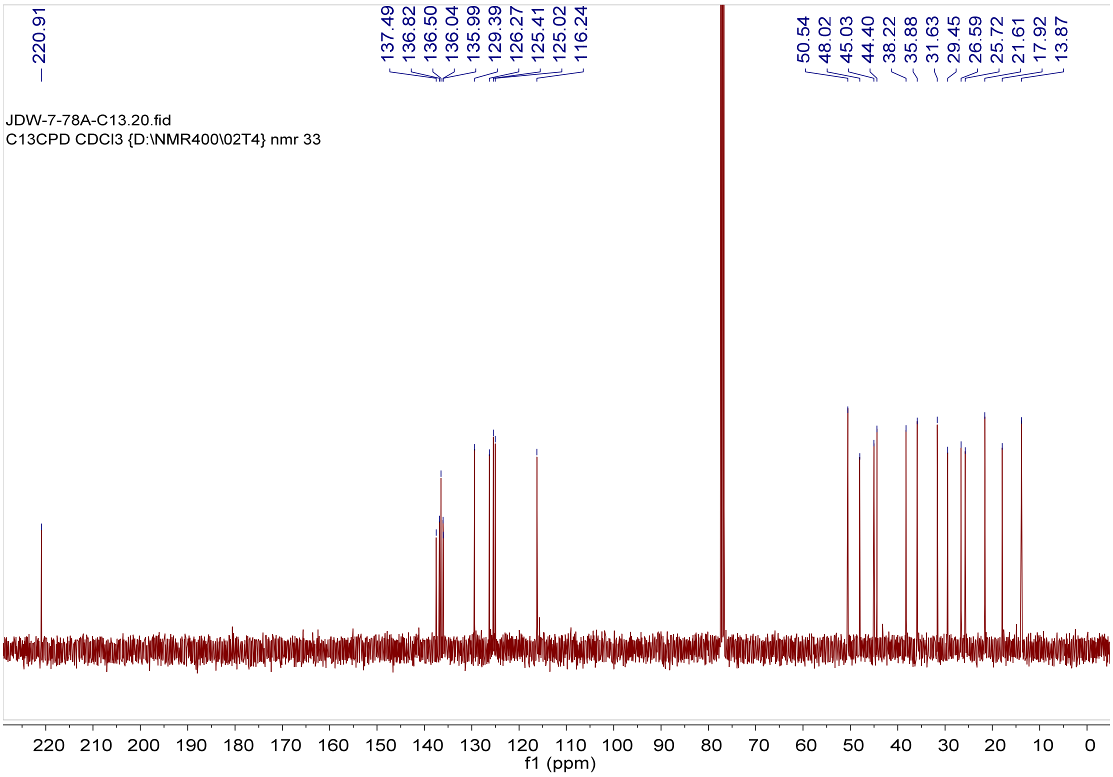

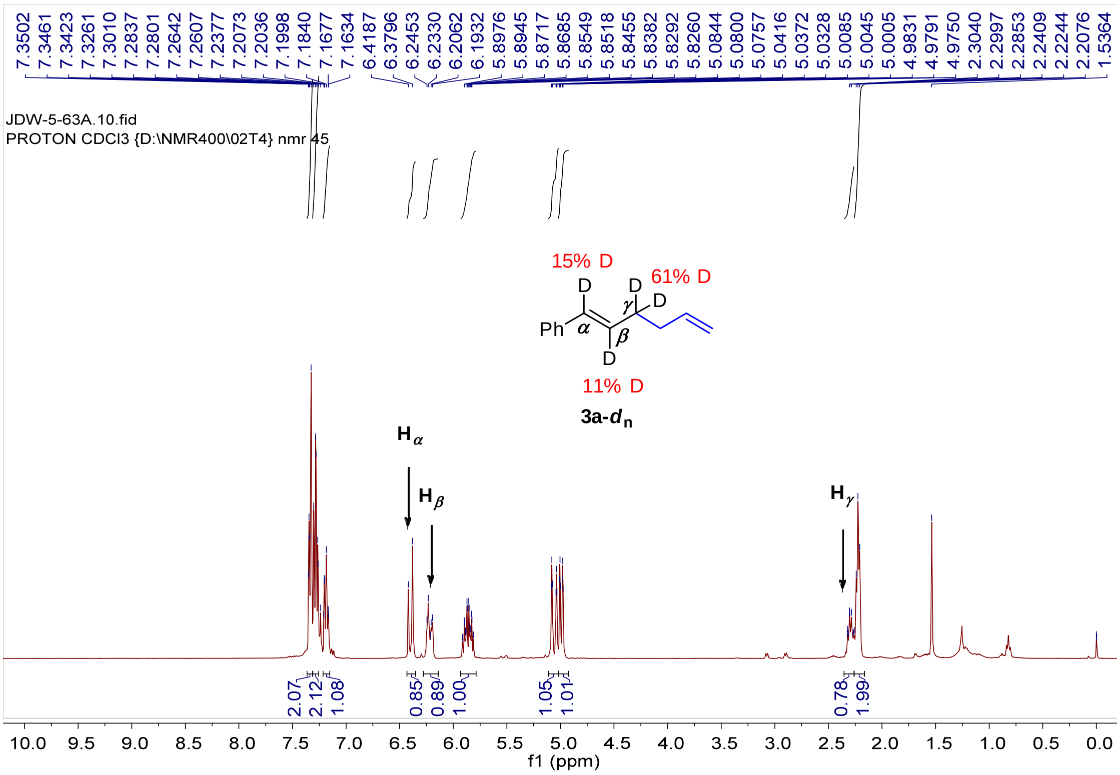
7.3502 7.3461 7.3423 7.3261 7.3010 7.2837 7.2801 7.2642 7.2607 7.2377 7.2073 7.2036 7.1998 7.1840 7.1677 7.1634 6.4187 6.3796 6.2453 6.2330 6.2062 6.1932 5.8976 5.8945 5.8717 5.8685 5.8549 5.8518 5.8455 5.8382 5.8292 5.8260 5.0844 5.0800 5.0757 5.0416 5.0372 5.0328 5.0085 5.0045 5.0005 4.9831 4.9791 4.9750 2.3040 2.2997 2.2853 2.2409 2.2244 2.2076 1.5364
JDW-5-63A.10.fid
PROTON CDCl3 {D:\NMR400\02T4} nmr 45
15% D
61% D
11% D
3a-d_n
H_α
H_β
H_γ
2.07 2.12 1.08 0.85 0.89 1.00 1.05 1.01 0.78 1.99
f1 (ppm)

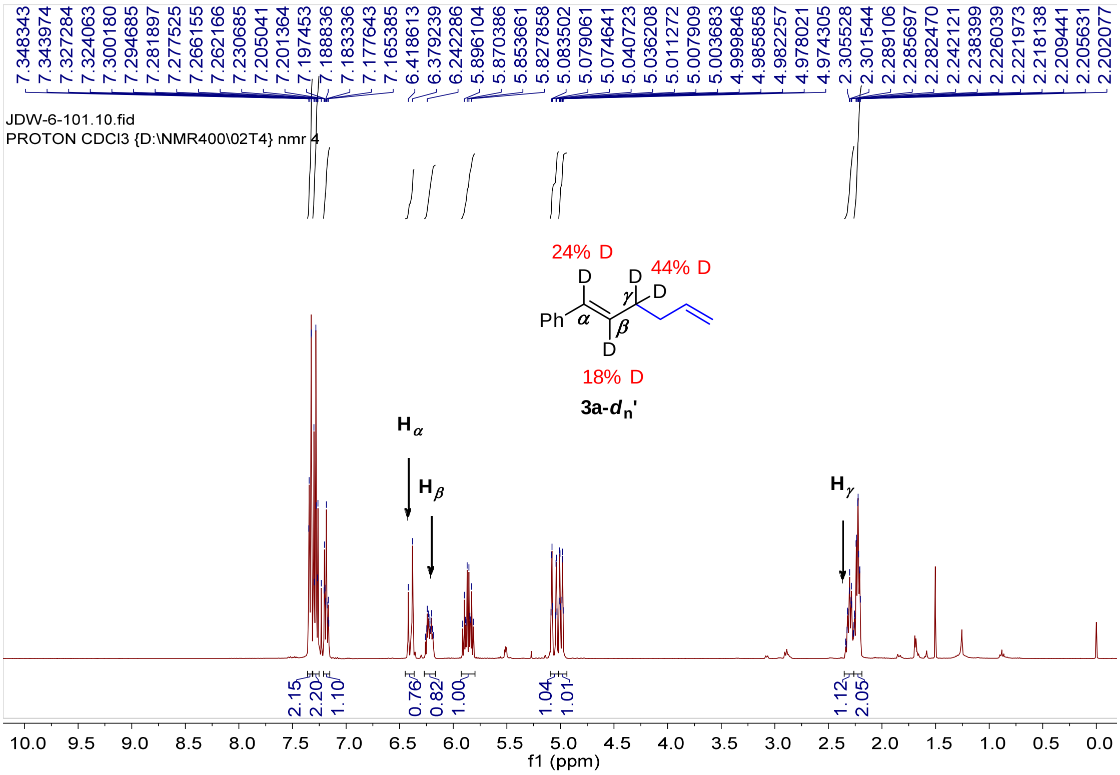
7.348343 7.343974 7.327284 7.324063 7.300180 7.294685 7.281897 7.277525 7.266155 7.262166 7.230685 7.205041 7.201364 7.197453 7.188836 7.183336 7.177643 7.165385 6.418613 6.379239 6.242286 5.896104 5.870386 5.853661 5.827858 5.083502 5.079061 5.074641 5.040723 5.036208 5.011272 5.007909 5.003683 4.999846 4.985858 4.982257 4.978021 4.974305 2.305528 2.301544 2.289106 2.285697 2.282470 2.242121 2.238399 2.226039 2.221973 2.218138 2.209441 2.205631 2.202077
JDW-6-101.10.fid
PROTON CDCl3 {D:\NMR400\02T4} nmr 4
24% D
44% D
18% D
3a-d_n'
H_α
H_β
H_γ
2.15 2.20 1.10 0.76 0.82 1.00 1.04 1.01 1.12 2.05
f1 (ppm)

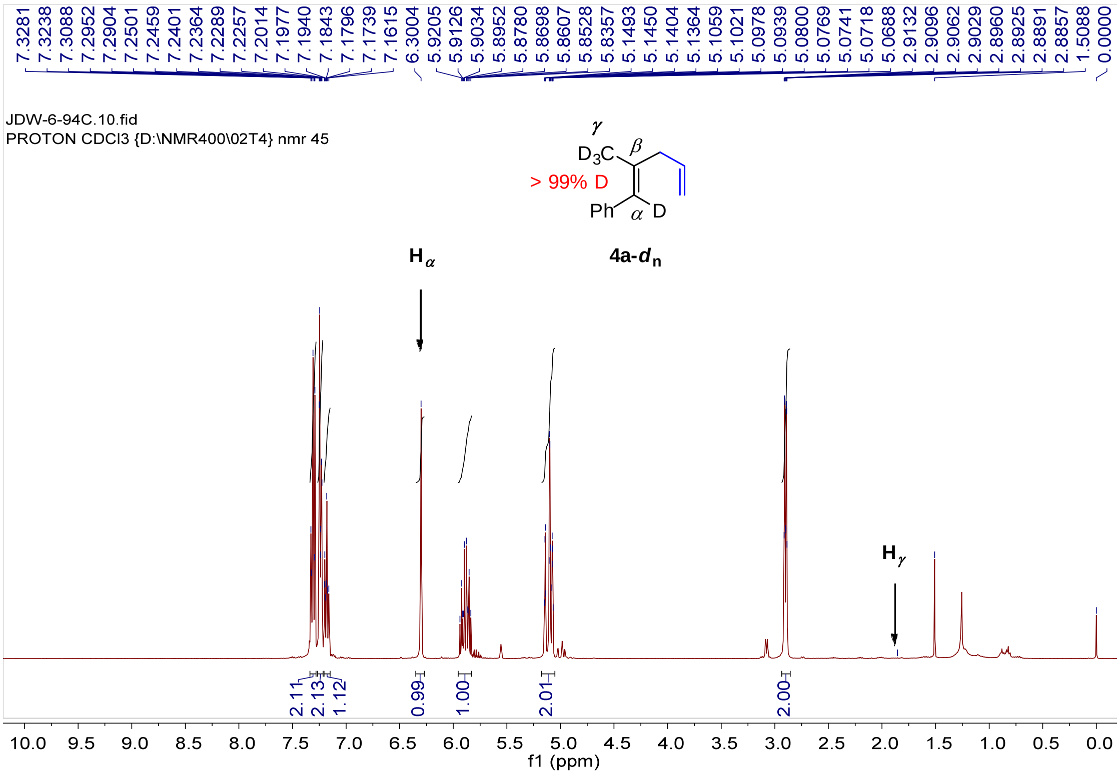
7.3281 7.3238 7.3088 7.2952 7.2904 7.2501 7.2459 7.2401 7.2364 7.2289 7.2257 7.2014 7.1977 7.1940 7.1843 7.1796 7.1739 7.1615 6.3004 5.9205 5.9126 5.9034 5.8952 5.8780 5.8698 5.8607 5.8528 5.8357 5.1493 5.1450 5.1404 5.1364 5.1059 5.1021 5.0978 5.0939 5.0800 5.0769 5.0741 5.0718 5.0688 2.9132 2.9096 2.9062 2.9029 2.8960 2.8925 2.8891 2.8857 1.5088 0.0000
JDW-6-94C.10.fid
PROTON CDCl3 {D:\NMR400\02T4} nmr 45
γ
D3C
β
> 99% D
Ph
α
D
4a-dn
Hα
Hγ
2.11
2.13
1.12
0.99
1.00
2.01
2.00
10.0 9.5 9.0 8.5 8.0 7.5 7.0 6.5 6.0 5.5 5.0 4.5 4.0 3.5 3.0 2.5 2.0 1.5 1.0 0.5 0.0
f1 (ppm)

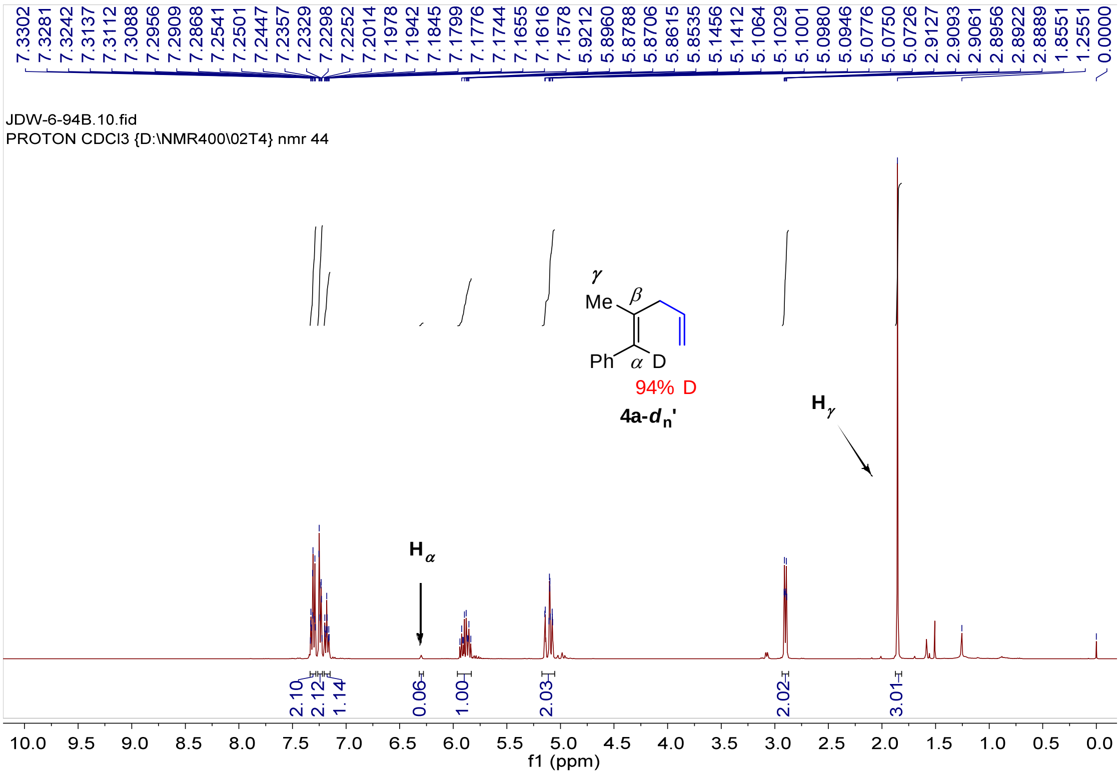
7.3302 7.3281 7.3242 7.3137 7.3112 7.3088 7.2956 7.2909 7.2868 7.2541 7.2501 7.2447 7.2357 7.2329 7.2298 7.2252 7.2014 7.1978 7.1942 7.1845 7.1799 7.1776 7.1744 7.1655 7.1616 7.1578 5.9212 5.8960 5.8788 5.8706 5.8615 5.8535 5.1456 5.1412 5.1064 5.1029 5.1001 5.0980 5.0946 5.0776 5.0750 5.0726 2.9127 2.9093 2.9061 2.8956 2.8922 2.8889 1.8551 1.2551 0.0000
JDW-6-94B.10.fid
PROTON CDCl3 {D:\NMR400\02T4} nmr 44
γ
Me
β
Ph
α
D
94% D
4a-dn'
Hγ
Hα
2.10
2.12
1.14
0.06
1.00
2.03
2.02
3.01
10.0 9.5 9.0 8.5 8.0 7.5 7.0 6.5 6.0 5.5 5.0 4.5 4.0 3.5 3.0 2.5 2.0 1.5 1.0 0.5 0.0
f1 (ppm)
